# Genome of the Avirulent Human-Infective Trypanosome—*Trypanosoma rangeli*

**DOI:** 10.1371/journal.pntd.0003176

**Published:** 2014-09-18

**Authors:** Patrícia Hermes Stoco, Glauber Wagner, Carlos Talavera-Lopez, Alexandra Gerber, Arnaldo Zaha, Claudia Elizabeth Thompson, Daniella Castanheira Bartholomeu, Débora Denardin Lückemeyer, Diana Bahia, Elgion Loreto, Elisa Beatriz Prestes, Fábio Mitsuo Lima, Gabriela Rodrigues-Luiz, Gustavo Adolfo Vallejo, José Franco da Silveira Filho, Sérgio Schenkman, Karina Mariante Monteiro, Kevin Morris Tyler, Luiz Gonzaga Paula de Almeida, Mauro Freitas Ortiz, Miguel Angel Chiurillo, Milene Höehr de Moraes, Oberdan de Lima Cunha, Rondon Mendonça-Neto, Rosane Silva, Santuza Maria Ribeiro Teixeira, Silvane Maria Fonseca Murta, Thais Cristine Marques Sincero, Tiago Antonio de Oliveira Mendes, Turán Peter Urmenyi, Viviane Grazielle Silva, Wanderson Duarte DaRocha, Björn Andersson, Álvaro José Romanha, Mário Steindel, Ana Tereza Ribeiro de Vasconcelos, Edmundo Carlos Grisard

**Affiliations:** 1 Universidade Federal de Santa Catarina, Florianópolis, Santa Catarina, Brazil; 2 Universidade do Oeste de Santa Catarina, Joaçaba, Santa Catarina, Brazil; 3 Department of Cell and Molecular Biology, Science for Life Laboratory, Karolinska Institutet, Stockholm, Sweden; 4 Laboratório Nacional de Computação Científica, Petrópolis, Rio de Janeiro, Brazil; 5 Universidade Federal do Rio Grande do Sul, Porto Alegre, Rio Grande do Sul, Brazil; 6 Universidade Federal de Minas Gerais, Belo Horizonte, Minas Gerais, Brazil; 7 Universidade Federal de São Paulo - Escola Paulista de Medicina, São Paulo, São Paulo, Brazil; 8 Universidade Federal de Santa Maria, Santa Maria, Rio Grande do Sul, Brazil; 9 Universidad del Tolima, Ibagué, Colombia; 10 Biomedical Research Centre, School of Medicine, Health Policy and Practice, University of East Anglia, Norwich, United Kingdom; 11 Universidad Centroccidental Lisandro Alvarado, Barquisimeto, Venezuela; 12 Universidade Federal do Rio de Janeiro, Rio de Janeiro, Rio de Janeiro, Brazil; 13 Centro de Pesquisas René Rachou, Fundação Oswaldo Cruz, Belo Horizonte, Minas Gerais, Brazil; 14 Universidade Federal do Paraná, Curitiba, Paraná, Brazil; University of Georgia, United States of America

## Abstract

**Background:**

*Trypanosoma rangeli* is a hemoflagellate protozoan parasite infecting humans and other wild and domestic mammals across Central and South America. It does not cause human disease, but it can be mistaken for the etiologic agent of Chagas disease, *Trypanosoma cruzi*. We have sequenced the *T. rangeli* genome to provide new tools for elucidating the distinct and intriguing biology of this species and the key pathways related to interaction with its arthropod and mammalian hosts.

**Methodology/Principal Findings:**

The *T. rangeli* haploid genome is ∼24 Mb in length, and is the smallest and least repetitive trypanosomatid genome sequenced thus far. This parasite genome has shorter subtelomeric sequences compared to those of *T. cruzi* and *T. brucei*; displays intraspecific karyotype variability and lacks minichromosomes. Of the predicted 7,613 protein coding sequences, functional annotations could be determined for 2,415, while 5,043 are hypothetical proteins, some with evidence of protein expression. 7,101 genes (93%) are shared with other trypanosomatids that infect humans. An ortholog of the *dcl2* gene involved in the *T. brucei* RNAi pathway was found in *T. rangeli*, but the RNAi machinery is non-functional since the other genes in this pathway are pseudogenized. *T. rangeli* is highly susceptible to oxidative stress, a phenotype that may be explained by a smaller number of anti-oxidant defense enzymes and heat-shock proteins.

**Conclusions/Significance:**

Phylogenetic comparison of nuclear and mitochondrial genes indicates that *T. rangeli* and *T. cruzi* are equidistant from *T. brucei*. In addition to revealing new aspects of trypanosome co-evolution within the vertebrate and invertebrate hosts, comparative genomic analysis with pathogenic trypanosomatids provides valuable new information that can be further explored with the aim of developing better diagnostic tools and/or therapeutic targets.

## Author Summary

Comparative genomics is a powerful tool that affords detailed study of the genetic and evolutionary basis for aspects of lifecycles and pathologies caused by phylogenetically related pathogens. The reference genome sequences of three trypanosomatids, *T. brucei*, *T. cruzi* and *L. major*, and subsequent addition of multiple *Leishmania* and *Trypanosoma* genomes has provided data upon which large-scale investigations delineating the complex systems biology of these human parasites has been built. Here, we compare the annotated genome sequence of *T. rangeli* strain SC-58 to available genomic sequence and annotation data from related species. We provide analysis of gene content, genome architecture and key characteristics associated with the biology of this non-pathogenic trypanosome. Moreover, we report striking new genomic features of *T. rangeli* compared with its closest relative, *T. cruzi*, such as (1) considerably less amplification on the gene copy number within multigene virulence factor families such as MASPs, trans-sialidases and mucins; (2) a reduced repertoire of genes encoding anti-oxidant defense enzymes; and (3) the presence of vestigial orthologs of the RNAi machinery, which are insufficient to constitute a functional pathway. Overall, the genome of *T. rangeli* provides for a much better understanding of the identity, evolution, regulation and function of trypanosome virulence determinants for both mammalian host and insect vector.

## Introduction

Human trypanosomiases result in high morbidity and mortality, affecting millions of people in developing and underdeveloped countries. In Africa, Trypanosomiasis (sleeping sickness) is tsetse-transmitted and is caused by *Trypanosoma brucei gambiense* and *T. b. rhodesiense*; whereas, in the Americas, Trypanosomiasis (Chagas disease) is transmitted by triatomine bugs and is caused by *Trypanosoma cruzi*. *Trypanosoma rangeli* (Tejera, 1920) is a third human infective trypanosome species that occurs in sympatry with *T. cruzi* in Central and South America, infecting a variety of mammalian species, including humans [1]. Natural mixed infections involving *T. rangeli* and *T. cruzi* have been reported in a wide geographical area for both mammals and the triatomine insect vectors [2], [3].

Literature on serological cross-reactivity between *T. rangeli* and *T. cruzi* has documented an ongoing controversy, probably influenced by the parasite form and/or strain, the host infection time and the serological assay used. While several authors have reported serological cross-reactivity between *T. cruzi* and *T. rangeli* in assays of human sera by conventional immunodiagnostic tests [1], [4]–[6], others have reported no cross-reactivity when recombinant antigens or species-specific synthetic peptides are used [7]. Recently, some species-specific proteins were identified in *T. rangeli* trypomastigotes which may provide for an effective differential in serodiagnosis [8].

In contrast to *T. brucei* and *T. cruzi*, *T. rangeli* is considered non-pathogenic to mammalian hosts but harmful to insect vectors, especially those from the genus *Rhodnius*. It causes morphological abnormalities and death of triatomine nymphs during molting [9], [10]. *T. rangeli* is transmitted among mammals through an inoculative route during hematophagy [1]–[3]. The parasite life cycle in the triatomine is initiated by ingestion of trypomastigote forms during a blood meal on an infected mammal. After switching to its epimastigote form, the parasite multiplies and colonizes the insect gut, prior to invading the hemocoel through the intestinal epithelium. Once in the hemolymph, *T. rangeli* replicates freely and invades the salivary glands, wherein it differentiates into infective metacyclic trypomastigotes [1]. *T. rangeli* infection via the contaminative route (feces) may also occur, as observed for *T. cruzi*, given that infective trypomastigotes are also found in the vector gut and rectum.

Although *T. rangeli* has been found to infect more than 20 mammalian species from five different orders, the parasite's life cycle in these hosts is poorly understood. Between 48 to 72 hours after the inoculation of short metacyclic trypomastigotes (10 µm), a small number of large trypomastigotes (35–40 µm) are found in the bloodstream and appear to persist for 2–3 weeks, after which the infection becomes subpatent. Despite the lack of a visible parasites in the blood, the parasite has been isolated from experimentally infected mammals up to three years after infection [1]. However, neither extracellular nor intracellular multiplication of the parasite in the mammalian host has been clearly demonstrated thus far.

High intra-specific variability has been described between *T. rangeli* strains, using multiple molecular genetic markers [2], [11]–[16]. A strong association of *T. rangeli* genetic groups with their local triatomine vector species has been demonstrated, and it has been proposed that the geographic distribution of the parasite' genotypes is associated with a particular evolutionary line of *Rhodnius* spp., indicating diversification may be tightly linked to host-parasite co-evolution [11], [16]–[18].

The gene expression profiles of distinct forms and strains of *T. rangeli* representing the major phylogenetic lineages (KP1+ and KP1−) were assessed via sequencing of EST/ORESTES [19]. Despite the non-pathogenic nature of *T. rangeli* in mammals, comparison of these transcriptomic data with data from *T. cruzi* and other kinetoplastid species revealed the presence of several genes associated with virulence and pathogenicity in other pathogenic kinetoplastids, such as *gp63*, sialidases and oligopeptidases.

Although *T. rangeli* is not particularly pathogenic in mammals, in light of its resemblance, sympatric distribution and serological cross-reactivity with *T. cruzi*, we decided to sequence and analyze the genome of *T. rangeli*. Here, we present the *T. rangeli* genome sequence and a comparative analysis of the predicted protein repertoire to reveal unique biological aspects of this taxon. Our findings may be useful for understanding the virulence and emergence of the human infectivity of *Trypanosoma* species.

## Methods

### Parasites culture and DNA extraction

Epimastigotes from the *T. rangeli* SC-58 (KP1−) and Choachí (KP1+) strains were maintained in liver infusion tryptose (LIT) medium supplemented with 15% FCS at 27°C after cyclic mouse-triatomine-mouse passages. The *T. cruzi* CL Brener and Y strains were maintained in liver infusion tryptose (LIT) medium supplemented with 10% FCS at 27°C. All samples tested negative for the presence of *Mycoplasma* sp. by PCR. For DNA sequencing, exponential growth phase epimastigotes from *T. rangeli* SC-58 strain were washed twice in sterile PBS and genomic DNA was extracted from parasites using the phenol/chloroform method.

### Pulsed-field gel electrophoresis (PFGE) and hybridization

Chromosomal DNA was isolated and fractionated via PFGE as described elsewhere [20], [21]. Briefly, 1.1% agarose gels were prepared in 0.5X TBE (45 mM Tris; 45 mM boric acid; 1 mM EDTA, pH 8.3), and agarose plugs containing the samples were loaded into the gels and electrophoresed using the Gene Navigator System (Amersham Pharmacia Biotech) at 13°C for 132 hours. The gels were then stained with ethidium bromide (EtBr) (0.5 mg/mL). The chromosomal bands of *T. rangeli* (Choachí and SC-58 strains) and *T. cruzi* (CL Brener clone) were fractioned using a protocol optimized to separate small DNA molecules in the CHEF Mapper system to assess the presence of minichromosomes.

### DNA library construction and sequencing

Library generation and sequencing were performed at the Computational Genomics Unit Darcy Fontoura de Almeida (UGCDFA) of the National Laboratory of Scientific Computation (LNCC) (Petrópolis, RJ, Brazil). 454 GS-FLX Titanium sequencing was utilized. Two sequencing libraries were prepared from *T. rangeli* SC-58 gDNA: one shotgun library (SG) and one 3 kb paired-end library (PE). Each library was constructed from 5 µg of genomic DNA (gDNA) following the GS FLX Titanium series protocols. All titrations, emulsions, PCR, and sequencing steps were carried out according to the manufacturer's protocol. One full PicoTiterPlate (PTP) was used to sequence each library.

### Genome assembly and automated functional annotation

In order to estimate the *T. rangeli* genome size, a pipeline developed at the Karolinska Institutet (KI) generated a genome assembly. Briefly, the 454 SFF (Standard Flowgram Format) files were processed using custom Perl scripts to generate paired-end (PE) FASTQ files. Subsequently, the SFF files were assembled without prior treatment using the Newbler assembler. The resulting assembly was scaffolded using SSPACE 2.1.0 with the generated 454 PE reads, and finally, assembly gaps were improved using GapFiller 1.11.

In order to specifically identify conserved protein coding regions, an alternate, protein-centric procedure was also utilized. A reference-guided assembly of *T. rangeli* genic regions was carried out using protein sequences from TriTrypDB as formerly described [22], resulting in an overview of the predicted parasite proteome. For this, 73,808 protein sequences were selected from the TriTrypDB (release 3.3 – http://tritrypdb.org/common/downloads/) and used for comparative analysis. All proteins retrieved from TriTrypDB were clustered by BBH (Bidirectional Best Hit), totaling 8,807 clusters. Parasite proteins that were not clustered were also used, for a total of 16,347 protein sequences. Sequences containing start codons different from ATG or containing stop codons in the middle of the sequence were filtered out. For each cluster, one protein was selected based on the following hierarchical criteria: (1) a *T. cruzi* protein with annotated function, or (2) a protein with annotated function from an organism different than *T. cruzi*, or (3) a *T. cruzi* hypothetical protein, or (4) the largest protein. The selected sequences were compared to reads from *T. rangeli* using *tBLASTn*, applying an E-value cut-off threshold of 1e–5 to define a set of significant reads to reconstruct each protein sequence. Each protein sequence was reconstructed with the counterpart set of reads selected using the software Newbler 2.5.3 according to the default parameters.

Automatic functional annotation of the *T. rangeli* genome was performed using the System for Automated Bacterial Integrated Annotation (SABIA) [23], including the previously generated and annotated EST/ORESTES database [19] and proteomic data obtained from surface of *T. rangeli* trypomastigotes [8].

The assembled nucleotide sequences were translated to aminoacid sequence and annotated according to the following criteria:

- Proteins with *BLASTp* hits in the KEGG database and with a minimum 60% coverage of both the query and the subject sequence: the first ten hits were analyzed, and the product was imported from KEGG ORTHOLOGY (KO) if one was associated with the hit, or from the KEGG GENES definition if no KO was associated with the first ten hits.
- Proteins with *BLASTp* hits in NCBI-nr, UniProtKB/Swiss-Prot or TCDB [24] databases and with a subject and query coverage ≥60% were assigned as annotated or hypothetical, depending on the annotation imported from the database.
- Proteins with no *BLASTp* hits in the databases mentioned above and no InterPro results or CDSs that did not fit the above criteria were designated hypothetical.
- Note – some proteins with hypothetical function have confirmed protein expression by MS/MS [8].

### Mobile genetic elements

Transposable elements were screened in genome assembly (KI) based on similarity using *BLASTn*, *tBLASTn* and *tBLASTx* tools [25]. As queries, the Repbase sequences described for the Euglenozoa group were used [26]. The *BLAST* results were filtered using the following parameters (e-value≤0.01, identity ≥50%, score≥80), *tBLASTx* (e-value≤0.01, identity ≥30%, score≥100) and *tBLASTn* (e-value≤0.01, identity ≥30%, score≥100). The retrieved sequences (protein and nucleotide) were aligned with the reference sequences and were manually curated. For *ab initio* searches, the software RepeatScout, release 1.0.5 was used [27].

### Gene copy number estimation

Peptides sequences from nine selected trypanosomatid multigene families (MASP, GP63, Trans-sialidase, Amastin, DGF, KMP-11, Tuzin, RHS and Mucin) were downloaded from TriTrypDB (tritrypdb.org). *T. rangeli* reads were then aligned against all members of each multigene family using *BLASTx* algorithm [25] and the reads from the best hits were selected. Those reads were assembled using CAP3 [28] and the resulting contigs were re-aligned against the NR (non-redundant) database from GenBank (https://www.ncbi.nlm.nih.gov/genbank/) and manually inspected to verify that they belong to the aforementioned multigene families. These validated contigs were used to construct a database corresponding to a subset of *T. rangeli* coding sequences belonging to the selected multigene families, except for the mucin genes. To determine gene copy number, the entire read dataset from the *T. rangeli* genome and all contigs generated, as described above, were aligned using reciprocal MegaBLAST and all reads corresponding to each contig were selected. After checking, the cut off for minimal identity (with no convergence in reads picking) was set as 95% identity, 10e-15 e-value and at least 80% of read coverage. The best hits were computed and used to calculate the read depth for each nucleotide and the regions covered with the highest rates were selected for the downstream analyses. The selected regions from each contig displaying high coverage values were realigned to NR protein database to verify specific multigene family before the copy numbers for each contig were calculated using the nucleotide by nucleotide coverages obtained with the z-score algorithm. The final coverage for each contig was then calculated after dividing the z-score value by the calculated genome sequencing coverage of 13.78. For all multigene families we added the values obtained as a copy number estimation for each contig to determine the final values displayed as the gene copy number of each family. For mucin genes, because signal peptide sequences are highly conserved in the different members of this family, the read coverage was carried only for the first 75 nucleotide present in the AUPL00006796 gene. To validate our method for copy number estimations and also to verify that the cutoff values were accurate this pipeline was applied to three genes known to be present as single copy genes in most trypanosomatid genomes (*msh2, msh6* and *gpi8*).

### Phylogenomic analyses of the Trypanosomatidae family

A phylogenomic analysis was carried out using all orthologous proteins from distinct species of the Trypanosomatidae family (*T. rangeli* SC-58, *T. cruzi* CL Brener Esmeraldo-like, *T. cruzi* CL Brener non-Esmeraldo-like, *T. cruzi* Sylvio X10, *T. brucei*, *L. braziliensis*, *L. infantum* and *L. major*). The multi-FASTA ortholog files containing the best representative of each trypanosomatidae protein sequence were used as inputs for multiple alignments with the default parameters of the CLUSTAL Omega algorithm [29]. All alignments were visually inspected and manually annotated whenever necessary the removal of low quality alignments. Subsequently, protein concatenation of the 1,557 alignment files obtained was carried out using SCaFos software [30].

Phylogenies from the concatenated deduced amino acid sequences of all species were estimated through both protein distance and probabilistic methods, using the PHYLIP package [31] and TREE-PUZZLE [32], respectively. The Seqboot program of the PHYLIP package was used to generate multiple 100-bootstrapped datasets, which were submitted to ProtDist software to compute a distance matrix under the JTT (Jones-Taylor-Thornton) model of amino acid replacement. The neighbor-joining (NJ) method [33] was applied to the resultant multiple datasets, implemented in Neighbor software, which constructed trees via successive clustering of lineages.

The quartet-puzzling [34] search algorithm implemented by TREE-PUZZLE was used to reconstruct phylogenetic trees based on maximum likelihood (ML). The Jones-Taylor-Thornton (JTT) model of amino acid substitution was applied. The quartet-puzzling tree topology was based on 1,000 puzzling steps. The consensus tree was constructed considering a 50% majority rule consensus. The TreeView program [35] and MEGA 5 [36] were used to visualize and edit the resultant phylogenies.

### Kinases

All protein kinase and phosphatidylinositol kinase sequences were selected and manually curated and re-annotated using the following software: Kinomer v. 1.0 web server [37], Kinbase (http://www.kinase.com/kinbase/), SMART (http://smart.embl-heidelberg.de/), Interproscan (http://www.ebi.ac.uk/Tools/pfa/iprscan/) and Motifscan (http://myhits.isb-sib.ch/cgi-bin/motif_scan). The presence of accessory domains and the domain architecture of some proteins, such as those from the AGC group, were decisive in classifying them into a group. PIK and PIK-related kinases were classified according to previous reports [38]–[41].

### Repetitive sequences

Analyses were performed using Tandem Repeat Finder (TRF) [42] and Tandem Repeat Assembly Program (TRAP) [43] software. The *T. rangeli* genome assembly (KI) and transcriptome [19] (2.45 Mb) sequences were submitted to TRF using the default parameters, except for minimum score of 25, as were 32.5 Mb of *T. cruzi* CL Brener Esmeraldo-like genome sequences from TriTrypDB using the same software parameters. The TRF output files were compiled using TRAP software, and we categorized the repeat sequences into four groups: microsatellites (1 to 6 nucleotides), unclassified (7 to 11 nucleotides), minisatellites (12 to 100 nucleotides) and satellite sequences (up to 100 nucleotides). The abundance, frequency and density of all *T. rangeli* repeat categories were calculated. Microsatellite classes were also analyzed considering all possible combinations; e.g., the repeat locus AGAT also included GATA, ATAG, TAGA and the reverses complements TCTA, CTAT, TATC and ATCT.

### Functional characterization of the RNAi machinery

To identify RNAi-related genes in the *T. rangeli* genome assembly, a set of 39 primers targeting the five genes constituting the RNAi machinery were designed and used to amplify these genes from the parasite genome by PCR. The PCR products were then purified using the Illustra GFX PCR DNA and Gel Band Purification kit (GE Healthcare) and cloned into pGEM-T-Easy vectors (Promega) or directly sequenced. Both strands of the PCR products or inserts were sequenced in a MegaBase automated sequencer, as directed by the manufacturer (GE Healthcare). After quality assessment using the Phred/Phrap/Consed package, sequences showing a Phred>30 were used along with the genome sequences to assemble the RNAi genes. Alignment of the consensus *T. rangeli* sequences with the *T. brucei* RNAi genes (TriTrypDB accession numbers Tb927.10.10850, Tb927.8.2370, Tb927.3.1230, Tb10.6k15.1610 and Tb927.10.10730) was carried out using MultiAlin [44].

Functional characterization of the *T. rangeli* RNAi machinery was performed using parasites transfected with the pTEXeGFP plasmid, kindly donated by Dr. John Kelly (LSHTM, UK). Silencing of eGFP was conducted using the TriFECTa exogenous reporter gene EGFP-S1 DS Positive Control (IDT) or the eGFP antisense siRNA EGFP-AS (5′-UGC AGA UGA ACU UCA GGG UCA-3′). Vero cells transfected with the pEGFP e1 plasmid (Clontech) were used as a positive control. All transfections were carried out in biological triplicates using a Nucleofector II device and the Human T Cell Nucleofector kit (Lonza). eGFP expression and silencing was assessed in both parasites and cells by Western blotting, flow cytometry analysis (FACS), direct fluorescence (FA) and qPCR. In the Western blot assays, an anti-GFP antibody (Santa Cruz Biotechnology) diluted 1∶2,000 was employed, according to standard protocols, and flow cytometry was carried out in a FACSCanto II (BD) apparatus.

Additionally, the functionality of the *T. rangeli* RNAi machinery was assessed through the transfection of epimastigote forms with the TUBdsRNA-RFP plasmid [45]. The evaluation of cell morphology and detection of RFP fluorescence were carried out at 6, 12, 24, 48 and 72 hours post-transfection using a BX FL 40 microscope (Olympus).

## Results and Discussion

### General features of the *T. rangeli* genome

**Figure 1:**
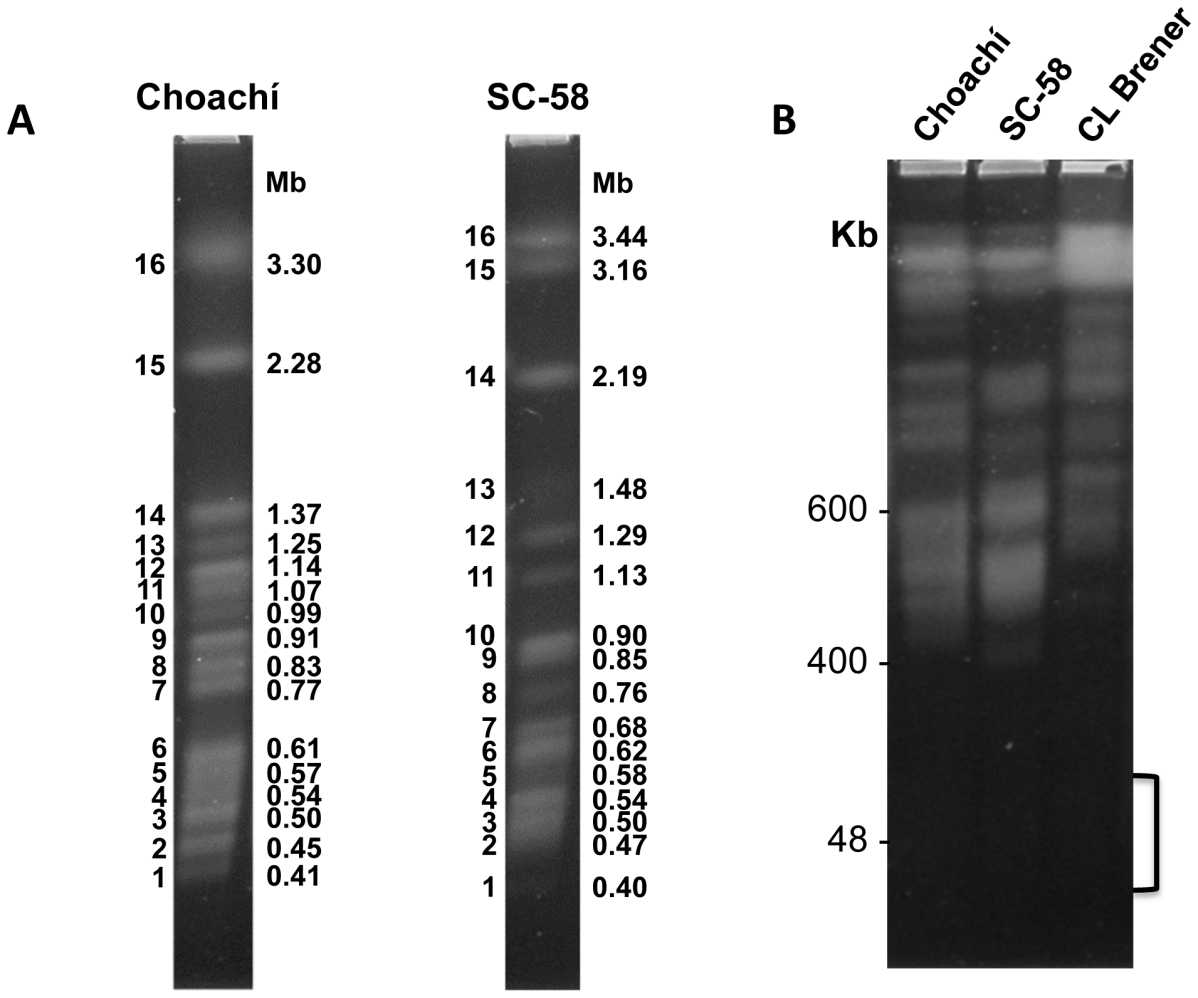
Molecular karyotype of *Trypanosoma rangeli*. **A**. Chromosomal bands of Choachí and SC-58 isolates were separated via PFGE and stained with ethidium bromide. The bands were numbered using Arabic numerals, starting from the smallest band. **B**. Chromosomal bands from *T. rangeli* (Choachí and SC-58 strains) and *T. cruzi* (clone CL Brener) were fractioned using a protocol optimized to separate small DNA molecules, revealing the absence of minichromosomes. The brackets represent the size range of *T. brucei* minichromosomes (30 and 150 kbp).

The karyotypes of representative strains from two major *T. rangeli* lineages [Choachí (KP1+) and SC-58 (KP1−)] were obtained via pulsed-field gel electrophoresis (PFGE). Two chromosomal-band size classes were defined: 1) megabase bands (those ranging from 2.19 to 3.5 Mb) 2) smaller bands, (ranging from 0.40 and 1.48 Mb). This analysis revealed at least 16 chromosomal-bands, whose sizes varied from 0.40 to 3.44 Mb; two megabase bands and 13–14 smaller bands (Figure 1A). We used specific PFGE separation conditions to confirm the absence of minichromosomes (Figure 1B), which are present in *T. brucei*, [46], but not in *T. cruzi*. The fluorescence intensity varied between these chromosomal bands, suggesting that co-migrating chromosomes are not necessarily homologous and that ploidy differences exist. The occurrence of aneuploidy has been demonstrated in different *T. cruzi* strains [21], [47] and in various species and isolates of *Leishmania* spp. [48], [49]. Of the 16 chromosomal bands identified, only seven were of a similar molecular size in the two *T. rangeli* isolates, confirming the existence of chromosomal size polymorphism, as demonstrated previously [50]–[52]. Therefore, analogously to *T. cruzi*, these 16 chromosomal bands may not reflect the actual number of chromosomes. Rather, this number is most likely higher than 16, as a single band may contain co-migrating heterologous chromosomes of similar sizes. Further studies will be needed to define the exact number of chromosomes and ploidy in *T. rangeli*.

Based on *ssu rDNA* and *gapdh* gene sequences, *T. rangeli* was phylogenetically positioned relatively closer to *T. cruzi* than to *T. brucei* [12]. This evolutionary proximity may also be reflected in the chromosomal organization of these species. It has been suggested that the common ancestor of trypanosomes exhibited smaller and more fragmented chromosomes and that fusion events occurred in the *T. brucei* lineage, leading to the smaller number of chromosomes currently observed [53]. Consistent with this idea, the chromosomal organization of *T. rangeli* also shows smaller and possibly more fragmented chromosomes, similar to those of *T. cruzi* [21].

**Figure 2:**
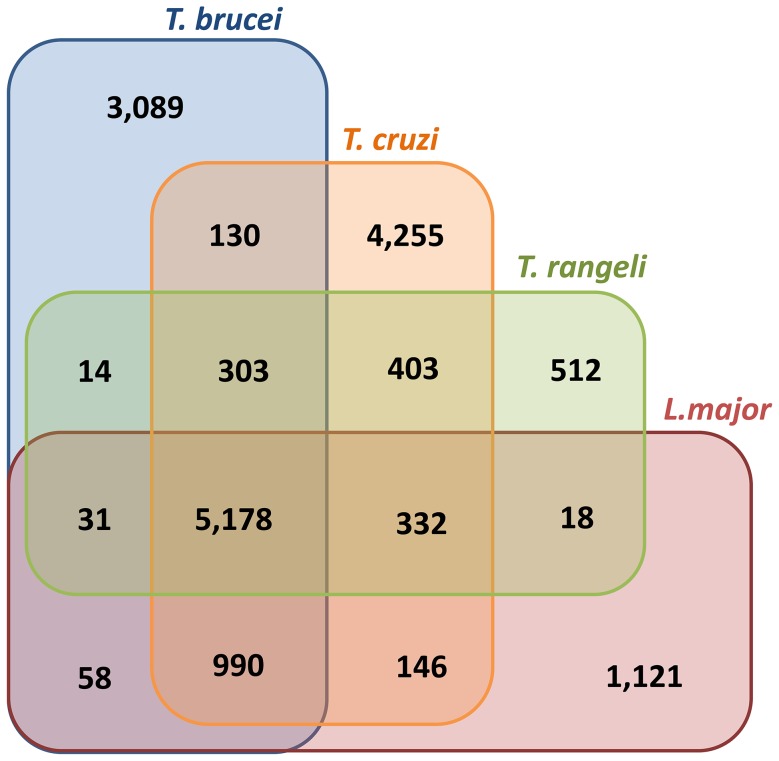
Number of gene clusters shared by the *T. rangeli*, *T. cruzi*, *T. brucei* and *L. major* genomes. Analyzes were performed using the following genome versions and gene numbers retrieved from the TriTrypDB: *Leishmania major* Friedlin (V. 7.0/8,400 genes), *Trypanosoma brucei* TREU927 (V. 5.0/10,574 genes), *Trypanosoma cruzi* CL Brener Esmeraldo (V. 7.0/10,342 genes) and Non-Esmeraldo (V. 7.0/10,834 genes). A total of 7,613 *T. rangeli* genes were used. BBH analysis used a cut-off value of 1e-05, positive similarity type and similarity value of 40% following manual trimming for comparison with COG analysis in [55] generating the numbers in the rectangles.

The general characteristics of the *T. rangeli* genome sequence are shown in Table 1 (GenBank accession AUPL00000000). The applied 454-based approach allowed the generation of 2,206,288 reads, which after reference-guided assembly to representative kinetoplastid gene sequences available at TriTrypDB, resulted in identification of a total of 7,613 coding sequences (CDS) from the *T. rangeli* reads. These CDSs include tRNAs encoding all 20 amino acids. In addition, we identify 33 genes corresponding to the typical trypanosomatid rRNAs (5.8S, 18S and 28S) (GenBank accession KJ742907). As has been observed for numerous other pathogenic and non-pathogenic trypanosomatids [54], a high percentage of *T. rangeli* genes (∼65.6%) encode hypothetical proteins. Among these genes, 44 show evidence of expression as revealed by *BLASTx* similarity to proteins detected via mass spectrometry on the surface of *T. rangeli* trypomastigotes [8]. Comparative sequence analysis revealed that 7,101 CDS (93%) of the identified *T. rangeli* genes are shared with other human pathogenic trypanosomes (Figure 2). *T. rangeli* shares 403 gene clusters exclusively with *T. cruzi*, thus reinforcing the phylogenetic relationship of these species. The conserved genome core of the 5,178 gene clusters present in all species (*T. rangeli*, *T. cruzi*, *T. brucei* and *L. major*) are mainly involved in fundamental biological processes and to host-parasite interactions (Figure 2), representing ∼84% of the TriTryp (*T. cruzi*, *T. brucei* and *L. major*) genome core [55].

**Table 1:** General characteristics of the *T. rangeli* genome.

Genome size (Mbp)	24
Coverage: Sequencing	13.78 X
G+C content (%): Genome	49.91
G+C content (%): CDS	54.27
Coding region (% of genome size)	37.77
Number of known protein CDSs*	2,415
Number of hypothetical CDSs	5,043
Number of partial/truncated CDSs	155
Average CDSs length (bp)	1,374
tRNA	55
Total number of CDSs	7,613

* Excluding proteins of unknown function.

In addition to reference-based gene assembly, a relatively high-quality *de novo* genome assembly was generated from paired-end reads utilizing the Karolinska Institutet pipeline. The final genome assembly contains 259 scaffolds with 4.42% gaps. Given the NG50 (statistic of scaffold lengths) of (202,734 bp) and the low repeat content of this genome, it is clear that most of the genome has been reconstructed. The assembly obtained by using the pipeline corroborates our draft reference-guided assembly data, suggesting a size of the *T. rangeli* genome of ∼24 Mb. Thus, the *T. rangeli* genome is the smallest and least repetitive trypanosomatid genome obtained to date including *T. cruzi* CL Brener and Sylvio X-10, *T. cruzi marinkellei*, *T. brucei* and *Leishmania* sp. [56]–[61].

### Phylogenomics of trypanosomatidae

**Figure 3:**
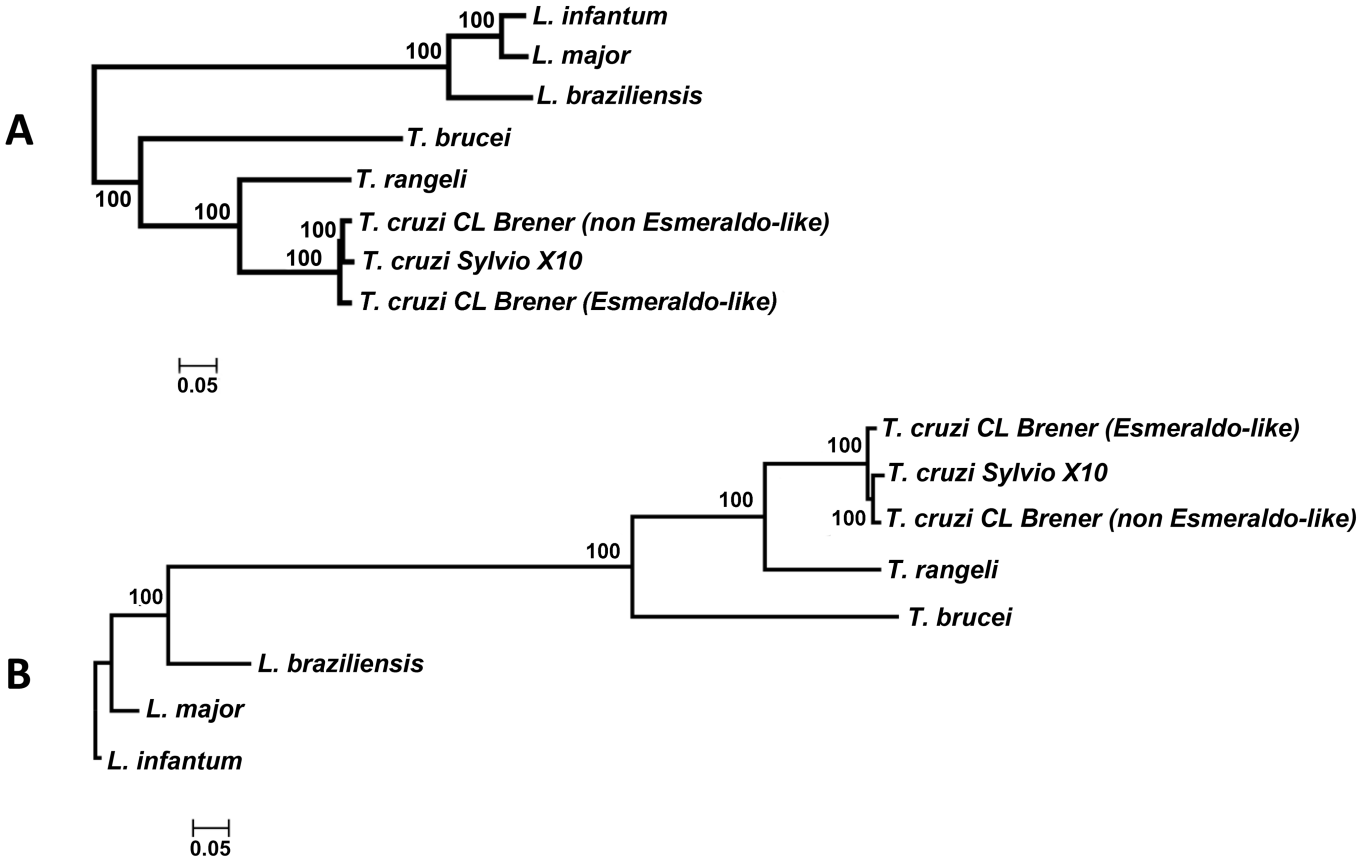
Evolutionary history of the Trypanosomatidae family obtained through a phylogenomic approach using (**A**) the neighbor joining (NJ) or (**B**) the maximum likelihood (ML) methods. In the NJ results, the percentage of replicate trees in which the associated taxa clustered together in the bootstrap test (100 replicates) is shown next to the branches. In the ML results, each internal branch indicates, as a percentage, how often the corresponding cluster was found among the 1,000 intermediate trees. The scale bar represents the number of amino acid substitutions per site.

Based on a total of 1,557 orthologous sequences representing different CDSs encoded by 8 different trypanosomatid genomes, an alignment of 964,591 concatenated amino acid residues was obtained and used to create NJ and ML tree topologies that were robust and revealed that South American trypanosomes (*T. rangeli* and *T. cruzi*) are equidistant from the African trypanosome (*T. brucei*) (Figures 3A and 3B). Despite the well-established genomic variability among *T. cruzi* strains, sequences derived from all strains CL Brener - Esmeraldo and non-Esmeraldo-like haplotypes - and Sylvio X10, clustered closer to *T. rangeli* than to *T. brucei* with high bootstrap values. The use of a phylogenomic approach to assess the evolutionary history of trypanosomatids clearly positioned *T. rangeli* closer to *T. cruzi* than *T. brucei* at the genomic level, corroborating former studies using single or a few genes [2], [3], [11], . *T. rangeli* and *T. cruzi* share conserved gene sequences with remarkably few genes or paralog groups that are unique to each one of the two species. Nevertheless, the divergence between *T. rangeli* and any *T. cruzi* strain is much greater than the differences among *T. cruzi* strains. As expected, all *Leishmania* species (*L. braziliensis*, *L. infantum*, and *L. major*) were clustered to a distinct branch.

### Simple repeats

The abundance, frequency and density of non-coding tandem repeat sequences found in the *T. rangeli* genome and transcriptome sequences; as well as a comparison of satellite DNA sequences to the *T. cruzi* haploid genome; are presented in Table S1. Approximately 1.27 Mb (6%) of the current *T. rangeli* genome assembly (∼24 Mb) is composed of tandem repeat sequences. Microsatellites are the most abundant repeats in both the *T. rangeli* (0.78 Mb, or 3.9%) and *T. cruzi* CL Brener (1.01 Mb, or 2.8%) genomes. We were able to identify 42,279 microsatellite loci, distributed in 400 non-redundant classes, in the *T. rangeli* genome sequence (Table S2). Approximately 4.7% (1,997) of these loci were found in the *T. rangeli* transcriptome [19] (Table S2). The microsatellite density and relative abundance in the *T. rangeli* genome assembly were estimated to be 38,678 bp/Mb and 3.87%, respectively. Interestingly, despite the relative abundance and the variation in the copy number of the 125 bp of satellite DNA observed in *T. cruzi* strains [62], these repeats were not found in the *T. rangeli* genome.

### Mobile genetic elements

Transposable elements (TEs) represent a significant source of genetic diversity, and the fraction of particular genomes that correspond to TEs is highly variable [63]. Furthermore, TEs have been widely used as tools for genome manipulation as transgenic vectors or for gene tagging in organisms ranging from different microbes to mammals [64], [65], including the protozoan parasites *Leishmania* sp., *Trypanosoma* sp. and *Plasmodium* sp. [66]–[68]. In the genomes of the kinetoplastid protozoa analyzed thus far, only retrotransposon elements have been found. Trypanosomes retain long autonomous non-LTR retrotransposons ∼ *ingi (T. brucei)* and *L1Tc* (*T. cruzi*); site-specific retroposons *SLACS (T. brucei)* and *CZAR* (*T. cruzi*); and short nonautonomous truncated versions (RIME, NARTc), in addition to degenerate *ingi*-related retroposons with no coding capacity (DIREs) as also observed for *L. major* [60], *L. infantum* and *L. braziliensis* [61]. A long autonomous LTR retrotransposon, designated *VIPER*, has also been described in *T. cruzi* [56], [57]. *L. braziliensis* contains *SLACS/CZAR*-related elements and the Telomeric Associated Transposable Elements (TATEs) [61].

Intact copies and putative autonomous TEs were not found in the *T. rangeli* genome. However, we identified 96 remnants of retrotransposons, which are most closely related to those of *T. cruzi*. The LTR retrotransposon *VIPER* was present as 39 copies, the non-LTR retroposons *ingi/RHS* as 51 copies; *L1TC*, five copies; and a single copy of *CZAR*. In contrast to *T. cruzi* and *T. brucei*, which maintain autonomous elements, and *L. braziliensis* with intact TATE elements at chromosome ends, *T. rangeli*, *L. major* and *L. infantum* harbors only degenerate elements, suggesting that TEs have been selectively lost during the course of recent evolution.

### Multigene families encoding surface proteins

Typically, a significant proportion of a trypanosomatid genome contains large families that encode surface proteins. Many of these proteins function as host cell adhesion molecules involved in cell invasion, as components of immune evasion mechanisms or as signaling proteins. We selected nine gene families that encode surface proteins present in *T. cruzi*, *T. brucei* and *Leishmania* spp. to search for orthologous sequences in the *T. rangeli* genome. Because the draft assemblies of the *T. cruzi* and *T. rangeli* genomes are still fragmented, we applied a read-based analysis to estimate the copy numbers of members of these families. Three single-copy genes that are known to have two distinct alleles in the *T. cruzi* CL Brener genome were also included in this analysis to validate our estimations. We found that the *T. rangeli* genome contains a smaller number of copies of three gene families, the MASPs, Mucins and Trans-sialidases, which are known to be present in far greater numbers in *T. cruzi*. Conversely, high copy numbers of amastin and *kmp-11* are present in the *T. rangeli* genome compared to *T. cruzi* (Table 2).

**Table 2:** Comparative number of genes per multicopy gene family in *T. rangeli* and *T. cruzi*.

Gene Family	*T. rangeli*	*T. cruzi*
	SC-58	CL Brener
**MASP**	50	1465
**GP63**	444	449
**Trans-sialidases**	120	1481
**Amastins**	72	27
**DGF**	422	569
**KMP-11**	148	40
**Tuzin**	34	83
**RHS**	689	777
**Mucin**	15	992
***msh6***	2	2
***msh2***	2	2
***gpi8***	2	2

*T. cruzi* amastins are small surface glycoproteins containing approximately 180 amino acids encoded by a gene family that has been subdivided into α-, β-, γ-, and δ-amastins and which are differentially expressed during the parasite life cycle [69], [70]. δ-amastins are mainly expressed by *T. cruzi* and *Leishmania* sp. intracellular amastigotes, a developmental stage that has not been observed during *T. rangeli* life cycle. Surprisingly, whereas *T. cruzi* has 27 copies of amastin genes, we estimate that 72 copies belonging to α-, β- and δ- amastin subfamilies are present in *T. rangeli*. Since the function of these proteins are still unknown, the study of their expression pattern and the significance of the expansion of this gene family in *T. rangeli* may shed new light into the role of these trypanosomatid specific surface glycoproteins.

Also in contrast to *T. cruzi* CL Brener strain, where forty alleles of genes encoding KPM-11 are present, there are 148 members in the KMP-11 in the *T. rangeli* genome. KMP-11 is a 92-amino acid antigen present in a wide range of trypanosomatids and is a target of the host humoral immune response against *Leishmania* spp. and *T. cruzi* infections, which, in the *T. cruzi* infection, induces an immunoprotective response [71]. The *T. rangeli* KMP-11 antigen shares 97% amino acid identity with its *T. cruzi* homologue [72]. These proteins are distributed in the cytoplasm, membrane, flagellum and flagellar pocket, most likely associated with the cytoskeleton of this protozoan [73]. The expansion of this family could have provided a selective growth advantage to *T. rangeli* in its insect vector. However, as a target for the immune response in mammals, it might have contributed to the poor pathogenicity of this organism.

The copy numbers of mucin glycoprotein-encoding genes, which are one of the largest and most heterogeneous gene families found in *T. cruzi* (TcMUC), are considerably reduced in *T. rangeli*. In *T. cruzi*, these surface glycoproteins cover the cell surface of several parasite stages and form a glycocalyx barrier [74]. Read coverage analysis of the region encoding the N-terminal conserved domain of the TcMUC family suggests the presence of only 15 copies in *T. rangeli* compared to 992 copies in *T. cruzi*. This finding is in agreement with the fact that only a few mucins were identified in the *T. rangeli* transcriptome [19], and only one TrMUC peptide was found through proteomic analysis [8]. In contrast to *T. cruzi*, *T. rangeli* lacks trans-sialidase activity, retaining only sialidase activity [75]. *T. cruzi* trans-sialidases (TS) are encoded by the largest gene family present in its genome. This enzyme catalyzes the transfer of sialic acid from sialylated donors present in host cells to the terminal galactose of mucin-glycoconjugates present at the parasite cell surface [76]. As a consequence of TS activity, in *T. cruzi*, large quanitities of multiple sialylated mucins form a protective coat when the parasite is exposed to the blood and tissues of the mammalian host. The relative paucity of the TrMUC repertoire correlates with the lower parasite load of *T. rangeli* in mammalian hosts and may in turn reflect the increased susceptibility to host immune mediators of *T. rangeli* compared with *T. cruzi*.

*T. cruzi* TS (TcTS) is a virulence factor integral to *T. cruzi* infection of the mammalian host [76], [77]. TcTS contains 12-amino acid repeats at the C-terminus, corresponding to the shed acute antigen (SAPA) [78], which is unnecessary for its activity but required for enzyme oligomerization and stability in the host [79]. This repeat is not present in *T. rangeli* sialidase sequences, and no *T. rangeli* proteins were detected in western blot assays using an anti-SAPA monoclonal antibody (unpublished results). In *T. cruzi*, TSs containing SAPA repeats are present only in infective trypomastigotes [80], while the TSs purified from epimastigotes lack the SAPA domain [81]. In addition to genes encoding the catalytic TS (subgroup Tc I), the trans-sialidase/sialidase superfamily in *T. cruzi* comprises eight subgroups, designated TcS I to VIII [82]. TcS group II encompasses proteins involved in host cell adhesion and invasion, and members of TcS group III display complement regulatory properties. The functions of the other groups are unknown, but all exhibit the conserved VTVxNVxLYNR motif, which is shared by all known TcS members [82], [83]. Sialidases/sialidase-like proteins similar to TcS groups I, II and III have been reported in *T. rangeli* [19], [84]–[86]. Here, we confirmed the presence of all TS subgroups in *T. rangeli* (Figure S1), although this parasite exhibits fewer members of the trans-sialidase/sialidase superfamily compared with *T. cruzi* (Table 2). It is therefore likely that all TS subgroups originated prior to the last common ancestor of the two species and that there was selective pressure in favor of the expansion and diversification of copies in *T. cruzi*. These observations also imply that the acquisition of SAPA repeats might have occurred after the appearance of the multiple gene family, when the *T. cruzi* ancestor gained mammalian infectivity, as proposed previously [81]. It has been suggested that the extensive sequence copy number expansion of the *T. cruzi* TS family could represent an immune evasion strategy driving the immune system to a series of spurious and non-neutralizing antibody responses [87]. It is tempting to speculate that the smaller number of copies of this large gene family found in *T. rangeli* could be related to the reduced virulence of this parasite in vertebrate hosts. Although, the expression of TS by both *T. rangeli* and *T. brucei* suggests a role for this enzyme during infections of the insect vector.

We identified 50 sequences in the *T. rangeli* genome encoding conserved domains of mucin-associated surface proteins (MASPs), which is fewer than that found in *T. cruzi*, in which the MASPs constitute the second largest gene family [57], [88]. Because MASPs are expressed at the surface of trypomastigotes and are highly polymorphic, the vast repertoire of MASP sequences present in the genome may contribute to the ability of *T. cruzi* to infect several host cell types and/or participate in host immune evasion mechanisms [89]. Changes in *T. cruzi* MASP family antigenic profiles during acute experimental infection have been established [89] and recent data has proposed a direct role for *T. cruzi* MASPs in host cell invasion (Najib El-Sayed, personal communication). Since *T. rangeli* lacks discernable ability to invade and multiply within the mammalian cells, the reduced repertoires of MASPs and of trans-sialidases in *T. rangeli* correlates may imply concerted action between these two groups of surface proteins during cell invasion and intracellular parasitism in *T. cruzi*.

### Immune response evasion

African trypanosomes (*T. brucei*, *T. congolense* and *T. vivax*) are blood-living, extracellular parasites, having variable surface glycoproteins (VSG) as key elements required for immune evasion in these species [90]. As with *T. cruzi*, sequences related to the (VSG) could not be discerned through rigorous searches of the *T. rangeli* genome.

In some strains of *T. rangeli*, the epimastigotes are highly resistant to complement-mediated lysis [91]. In this context, genes showing similarity to *gp160*, a member of the large super-family of trans-sialidases identified as complement regulatory protein (CRP, or GP160) in *T. cruzi* [92], are found in the *T. rangeli* genome. However, their sizes are smaller than the corresponding *T. cruzi* genes, and considering the domain conservation observed in this family, their function as complement regulatory proteins remains unproven. Other *T. cruzi* molecules have been shown to confer resistance to complement-mediated lysis, such as calreticulin, GP58/68 and the complement C2 receptor inhibitor trispanning (CRIT) [93]. Our data showed that CRIT protein is absent in *T. rangeli*.

### The *T. rangeli* kinetoplast

The mitochondrial genome of trypanosomes is a structure composed of concatenated large (maxi-) and small (mini-) circular DNAs. Minicircles are more abundant, comprising several thousand copies per genome, and are 1.6 to 1.8 kb long in *T. rangeli*. Minicircles encode gRNAs that are utilized in the editing of mitochondrial transcripts derived from maxicircle DNA, which are present at about 20 copies per genome. Minicircles exhibit heterogeneous and highly conserved regions [94]. Probes generated against conserved regions have been previously used as sensitive tools for discriminating *T. rangeli* and *T. cruzi* lineages [15].

We assembled the maxicircle of *T. rangeli* as a single contig of 25,288 bp. The length of this sequence is >10 kb longer than those sequenced from *T. cruzi* (Sylvio 15,185 bp, CL Brener 15,167 bp, Esmeraldo 14,935 bp). The maxicircle of *T. cruzi marinkellei* was found to be slightly longer (20,037 bp) than those of other *T. cruzi* strains. These length differences were attributed to variability of the repetitive region [59], [95]. Similarly, the *T. rangeli* maxicircle exhibits repetitive regions of ∼6 Kb that, along with non-coding regions, have increased the overall size by ∼15 Kb. The coding region of the *T. rangeli* maxicircle has maintained a high degree of synteny with that of *T. cruzi* (Figure S2). We found no *in silico* evidence of additional coding sequences outside this region. Transcripts from *rRNA*, *cyb*, *coII* and *nadh* were identified in the *T. rangeli* EST database [19].

### Telomeres

**Figure 4:**
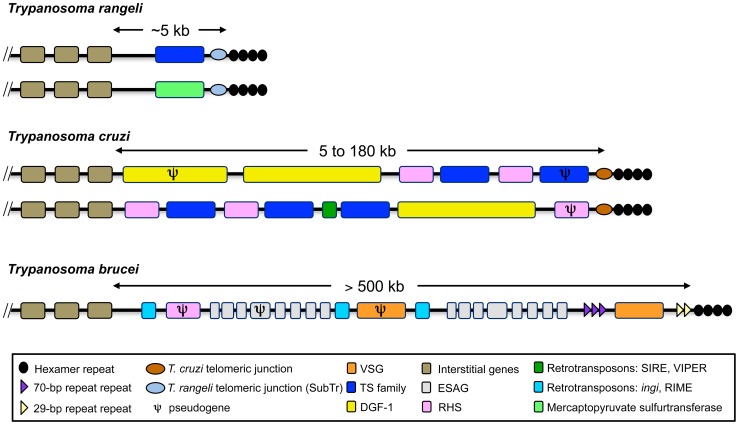
Representation of the telomeric and subtelomeric regions of *Trypanosoma rangeli*, *T. cruzi* and *T. brucei*. The two types of telomeres identified in *T. rangeli* and two others representing the heterogeneity of *T. cruzi* chromosome ends are shown. The size of the subtelomeric region, which extends between the telomeric hexamer repeats and the first internal core genes of the trypanosomes, is indicated below each map. Boxes indicate genes and/or gene arrays. The maps are not to scale. The *T. brucei* and *T. cruzi* maps were adapted from [55], [98].

**Figure 5:**
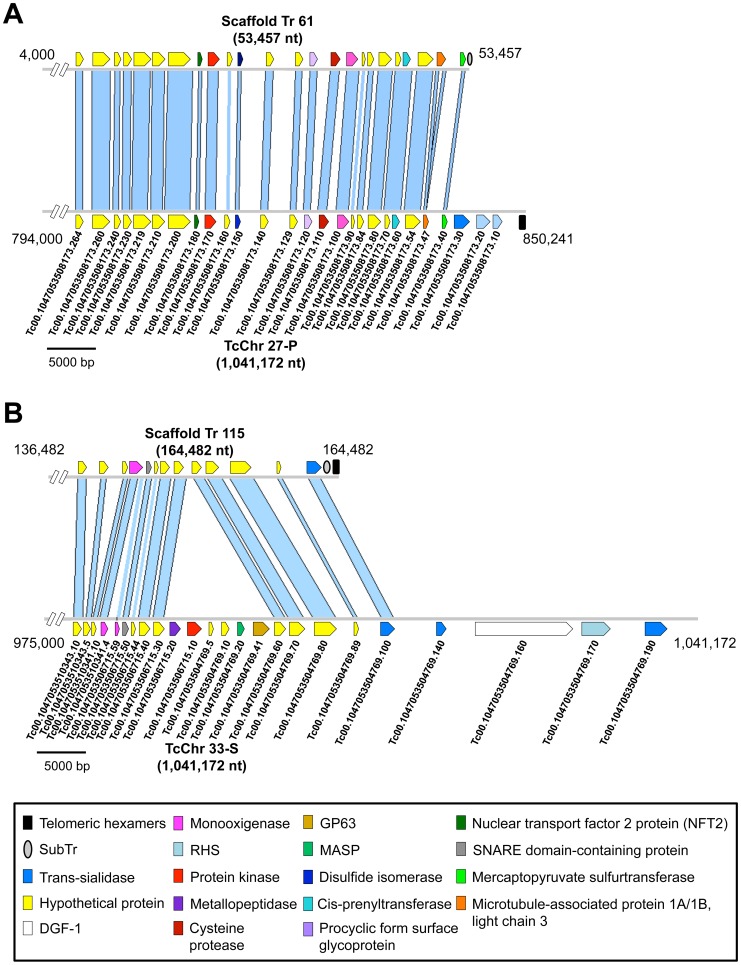
Synteny analysis between *Trypanosoma rangeli* scaffolds and organized contig ends of *T. cruzi*. The blue lines represent regions of homology between the contigs. Annotated genes and other sequence characteristics are indicated by colored boxes. Arrows indicate sense transcription. **A**. Comparison between Scaffold Tr 61 (4,000–53,457 nt) and TcChr27-P (794,000–850,241 nt). **B**. Comparison between Scaffold Tr 115 (136,482–164,482 nt) and TcChr33-S (975,000–1,041,172 nt). Contig ends were oriented in the 5′ to 3′ direction according to the TriTrypDB assemblies of *T. cruzi* scaffolds. The accession numbers of the annotated sequences in the *T. cruzi* scaffolds (TriTrypDB) are displayed below the sequences.

Three chromosome ends were identified in the genome assembled in this study (Figure S3) corresponding to telomere ends. These sequences contain previously described structures found in the terminal region of *T. rangeli* telomeres, which is characterized by a specific telomeric junction sequence in *T. rangeli* (SubTr) separating the hexameric repeats from interstitial gene sequences [96], [97]. Although *T. rangeli* (SubTr) and *T. cruzi* (Tc189) telomeric junctions share very low sequence identity, related sequences have been identified in several intergenic regions in both protozoa (mainly between *gp85* genes of the trans-sialidase superfamily), suggesting that the two structures could have a common origin. According to our analysis of the sequence immediately upstream of SubTr, two types of chromosome ends could be identified (Figure 4). In the first type, SubTr is preceded by a *gp85*/trans-sialidase gene/pseudogene, while the second exhibits a copy of the mercaptopyruvate sulfurtransferase gene. The presence of this single copy gene so close to the telomeric end of a chromosome in *T. rangeli* is interesting because it is absent at this location in *T. cruzi* telomeres where only pseudogenes belonging to multigene families have been found. Notwithstanding, the chromosome ends of *T. rangeli* differ from those of *T. brucei* and *T. cruzi* in that they exhibit a simpler homogeneous organization, with short subtelomeric regions [57]. The subtelomeric region extending between SubTr and the first internal (interstitial) chromosome-specific gene in the scaffolds analyzed here is quite short (∼5 kb) (Figure 4). Two of the analyzed scaffolds exhibit a high level of gene synteny with *T. cruzi* chromosome ends (CL Brener). However, this synteny is lost in subtelomeric regions due to the absence of interspersed “islands” of trans-sialidase, *dgf-1* and *rhs* genes/pseudogenes in the chromosomes of *T. rangeli* (Figure 5) [55], [98]. Therefore, the differences in subtelomeric structure observed between *T. rangeli* and *T. cruzi* are consistent with the reduced number of repeated sequences found in the genome of the former and with the expansion of these sequences in the latter.

Although telomerase activity has not been reported in *T. rangeli*, a putative telomerase reverse transcriptase (*tert*) gene, along with an ortholog of a telomerase-associated protein (TEP1) gene were identified in the genome of this parasite. Taken together, the presence of the *tert* and *tep1* genes and the lack of transposable elements or blocks of non-hexameric tandem repeat sequences at chromosome ends suggest that the maintenance of telomere length in *T. rangeli* is primarily due to telomerase activity.

Among the telomere-binding proteins, a putative TTAGGG binding factor (TRF2) homolog was identified in the *T. rangeli* genome. In *T. brucei*, TRF2 interacts with double-stranded telomeric DNA as a homodimer and is essential for maintaining the telomeric G-rich overhang [99]. Moreover, homologs of the RBP38/Tc38 and RPA-1 proteins, which are single-stranded DNA-binding factors involved in telomere maintenance mechanisms, and two other putative proteins (JBP1 and JBP2) participating in base J biosynthesis [100]–[102] were also detected in *T. rangeli*. Base J is a hypermodified DNA base localized primarily at telomeric regions of the genome of *T. brucei*, *T. cruzi* and *Leishmania* with elusive function. However, J in chromosome-internal positions has been associated with regulation of Pol II transcription initiation in *T. cruzi* [103], whereas in *Leishmania* sp. when present at the ends of long polycistronic transcripts, it was shown to be involved in transcription termination [104].

### Translation components

Most of the major components of the translation machinery found in other trypanosome and leishmania genomes are also found in *T. rangeli* (Table S3). In general, one copy of the genes encoding the aminoacyl-tRNA synthetases is present, except for glutaminyl-tRNA synthetase and aspartyl-tRNA synthetase, which display three copies each, and leucyl-tRNA synthetase, lysyl-tRNA synthetase, valyl-tRNA synthetase, tryptophanyl-tRNA synthetase, and seryl-tRNA synthetase, which exhibit two copies each. N-terminal mitochondrial targeting signals were also predicted in some of the deduced amino acid sequences of tRNA-synthetases from *T. rangeli*.

Compared to the other trypanosome genomes, similar numbers of genes encoding ribosomal proteins and other factors involved in translation were found in *T. rangeli* with some minor variation. For example, three copies of genes encoding eukaryotic initiation factor 5A were detected in *T. rangeli*, compared to two in *T. cruzi* and one in *T. brucei*. Only one copy of elongation factor 1-beta was identified in *T. rangeli*, compared to three in *T. cruzi* and *T. brucei* and there are eight paralogs of Elongation factor 1-alpha in *T. rangeli* that are similar to the paralogous expansion observed in *T. cruzi*, with eleven copies.

### RNA interference in *T. rangeli*: Is the RNAi machinery being dismantled?

In many eukaryotes, RNA interference (RNAi) is a cellular mechanism for controlling gene expression in a sequence-specific fashion. This phenomenon has been described in a large number of organisms, including *T. brucei*, *T. congolense*, *L. braziliensis* and *Giardia lamblia*. It is, however, absent in many other trypanosomes, such as *T. cruzi*, *L. major* and *L. donovani*, and other protozoa, such as *Plasmodium falciparum* [45], [105]–[107]. Since the discovery of RNAi in *T. brucei* [108], a total of five major components of the RNAi machinery have been identified, including cytosolic (TbDCL1) and nuclear (TbDCL2) dicers, the Argonaute 1 (TbAGO1) protein, and two additional RNA Interference Factors, designated TbRIF4 and TbRIF5. It has been proposed that TbRIF4 acts in the conversion of double-stranded siRNAs into single-stranded form, and TbRIF5 functions as an essential co-factor for the TbDCL1 protein [109]–[112].

By searching for orthologs of components of the RNAi machinery in the *T. rangeli* genome using the *T. brucei* protein sequences as queries in *tBLASTn* analyses, we found that four of the five components of the *T. brucei* RNAi machinery are present in the *T. rangeli* genome as pseudogenes, as they exhibit one or more stop codons or frame shifts. To further evaluate whether these defective genes were a strain-specific phenomena restricted to the SC-58 strain, another strain representative of the northernmost distribution of the parasite was also assayed via PCR amplification and sequenced using Sanger sequencing chemistry. In addition to punctual differences among the strains, large deletions in *T. rangeli ago1* and *dcl1* were found (Figure S4). Among these five RNAi components, only Dicer-like 2 can be functional, since it contains insertions and deletions that do not cause frame-shifts or a premature translational stop. The *T. rangeli* Dicer-like 2 protein is 54 amino acids shorter in its N-terminal portion, exhibiting approximately 30% identity with *T. congolense* and *T. brucei* DCL2, with higher conservation in the RNaseIII domain (C-terminus) (Figure S5). The explanation for why only *dcl2* was retained in the *T. rangeli* genome is unclear. However, it has been shown in *T. brucei*, that the *dcl2* knockout cell line shows reduced levels of CIR147 (Chromosomal Internal Repeats – 147 bp long) and SLACS siRNAs (Spliced Leader Associated Conserved Sequence) and accumulation of long transcripts derived from retrotransposons (*ingi* and SLACS) [110]. This TbDCL2 knockout cell line also showed an increasing in the RNAi response to exogenous dsRNA. It is, however, difficult to speculate whether TrDCL2 plays a similar role in *T. rangeli* because the TbAGO1 ortholog is defective in this organism, and TbAGO1 knockout cells shows phenotype overlap compared to TbDCL2 -/- parasites [110].

Furthermore, a gene encoding a member of the AGO/PIWI family without the PAZ domain (conferring small RNA binding activity) was found in the *T. rangeli* genome (AUPL00000858). It encodes a protein of 1,083 amino acids that shares highest identity with *T. cruzi* (71% identical), followed by *T. brucei* (58%) and *T. congolense* (52%) throughout its entire sequence. This gene is present in the genome of all trypanosomatids, including RNAi-negative parasites, but its function is still unknown [113]. It may be that the protein encoded can work together with the TrDCL2 as part of an RNA metabolism pathway, but further work is needed to test this hypothesis.

**Figure 6:**
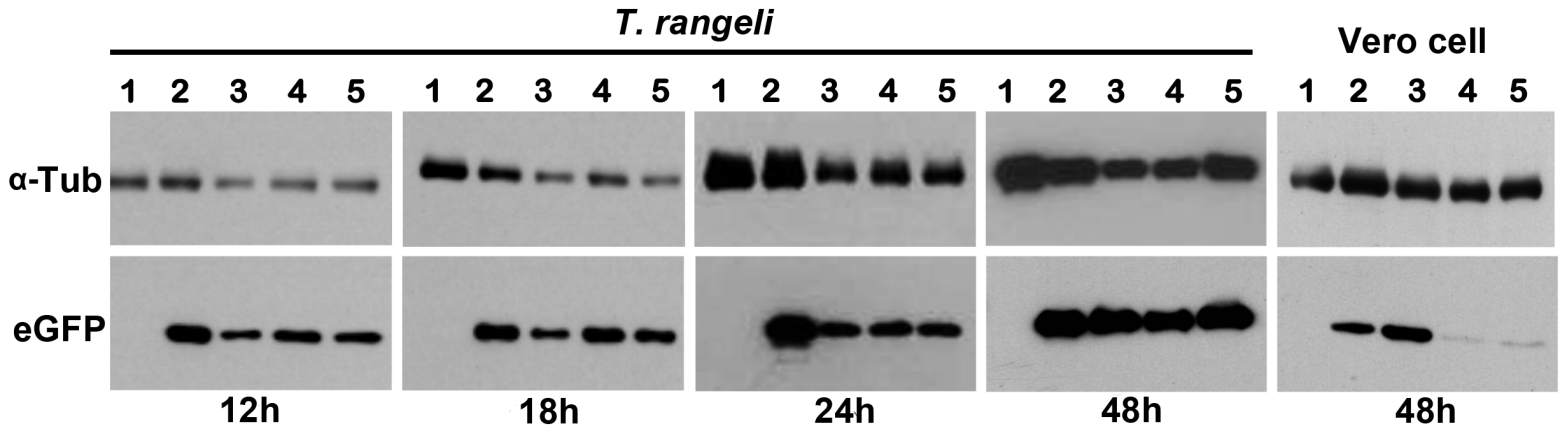
The RNAi machinery is not active in *Trypanosoma rangeli*. Western blot analysis of eGFP silencing via siRNA in *T. rangeli* and Vero cells expressing eGFP. For the Western blot assays, anti-GFP and anti-alpha tubulin antibodies were used. In each blot, wild-type cells (1), eGFP cells (2), eGFP cells transfected with Mock siRNA (3), eGFP cells transfected with EGFP-S1 DS Positive Control (IDT)(4) and eGFP cells transfected with eGFP antisense siRNA (5) are shown sequentially. The experiments were performed in biological triplicates.

In addition to re-sequencing PCR products corresponding to RNAi factors, the presence of a functional RNAi mechanism was investigated through transient transfections of a siRNA targeting eGFP, or a plasmid that can drive the expression of a long dsRNA targeting endogenous β-tubulin and a fluorescent marker (red fluorescent protein). In agreement with the *in silico* analysis, the transfection of eGFP-expressing cells or wild type parasites with the siRNA (Figure 6) or a plasmid encoding tubulin dsRNA, respectively, failed to inhibit eGFP expression or alter the parasite's morphology, which suggests an absence of a functional RNAi machinery in *T. rangeli*.

### Protein kinases and phosphatidylinositol kinases

The *T. rangeli* genome encodes 151 eukaryotic protein kinases (ePKs), which corresponds to 1.94% of the total coding sequences in the genome. Like other trypanosomatids, *T. rangeli* lacks members of the protein tyrosine kinase (PTK), tyrosine kinase-like (TKL) and receptor guanylate cyclases (RGC) groups. *T. rangeli* displays some ePKs with predicted transmembrane domains, including nine genes, in addition to five with a signal peptide (Table S4).

The protein kinases of eukaryotes are subdivided into 8 groups according to the nomenclature of Miranda-Saavedra and Barton (2007) [114] and KinBase (http://www.kinase.com/kinbase/). In the *T. rangeli* genome, the largest group is “Other” (kinases that could not be assigned to a specific group), with 40 members, followed by the CMGC (cyclin-dependent kinases, mitogen-activated protein kinases, glycogen synthase kinase 3 and CK2-related kinases) group, with 30 members, two of which are catalytically inactive. The least represented group is the casein kinases (CK1), with only two members. The other groups display 26 members in AGC (Protein kinase A, G and C families), 22 members in CAMK (Calcium and Calmodulin-regulated kinases) and 31 members in STE (Kinases related to MAPKs activation).

The phosphatidylinositol kinases (PIK) and PIK-related proteins of *T. rangeli* are described in Table S5. These are lipid kinases that play a key role in a wide range of cellular processes, such as cell growth and survival, vesicle trafficking, cytoskeletal reorganization and chemotaxis, cell adhesion, superoxide production and glucose transport [115]. Like *T. cruzi* [38], *T. rangeli* lacks a *tor-like 2* gene, although a truncated version of this gene without the catalytic domain has been identified. The accessory domains of the PIK-related families of both *T. cruzi* and *T. rangeli* can be seen in Table S6.

In addition, *T. rangeli* possesses four phosphatidylinositol phosphate kinases (PIPK), which have not been evaluated in other trypanosomatids as yet, including in *T. cruzi*. These kinases phosphorylate already-phosphorylated phosphatidyl inositols to form phosphatidylinositol bisphosphates. The PIPK functions have been mainly established for mice and humans, which include vesicular trafficking, membrane translocation, cell adhesion, chemotaxis, the cell cycle and DNA synthesis [116].

### DNA repair and recombination in *T. rangeli*

Genes that encode most of the proteins responsible for DNA repair and recombination mechanisms in other trypanosomatids were also found in *T. rangeli*, suggesting that this protozoan displays all of the known functional DNA repair pathways. In other organisms it has been demonstrated that errors generated during DNA replication can be corrected via DNA mismatch repair, involving the recruitment of heterodimers of MSH2 and MSH3 or MSH6, which signalize MLH1 and PMS1 binding [117]. Homologs of these proteins are present in *T. rangeli*, but in common with other trypanosomatids, no homolog of PMS2 was found [56], [57], [60]. Different DNA base modifications can be corrected via base excision repair [118]. Sequences encoding the OGG1, UNG and MUTY DNA glycosylases were identified. However, whether the long and short pathways are functional is a question that remains to be answered because important homologs, such as LIG3, XRCC1 and PARP, are missing. Lesions that alter DNA conformation can be repaired through nucleotide excision repair (NER) [119], and as with other trypanosomatids, *T. rangeli* contains sequences encoding most of components of the NER pathway, including proteins constituting the TFIIH complex. It has been shown that in *T. brucei*, two trypanosomatid-specific subunits of TFIIH (TSP1 and TSP2) are important for parasite viability because they participate in the transcription of the splice-leader gene [120]. Both proteins are also present in *T. rangeli*, as well in *T. cruzi* and *L. major*.

DNA recombination is an essential process involved in DNA repair and in the generation of genetic variability in these parasites. No major differences in genes encoding components of DNA recombination machinery were observed between *T. rangeli* and other trypanosomatids [121]. They all exhibit genes encoding MRE11, RAD50, KU70 and 80, BRCA2 and RAD51, which play important roles in homologous recombination (HR) and non-homologous end joining (NHEJ). However, *T. rangeli* lacks homologs of DNA Ligase IV and XRCC4, like other trypanosomatids, indicating that it does not exhibit a functional NHEJ [122].

### Antioxidant defense and stress responses in *T. rangeli*

**Figure 7:**
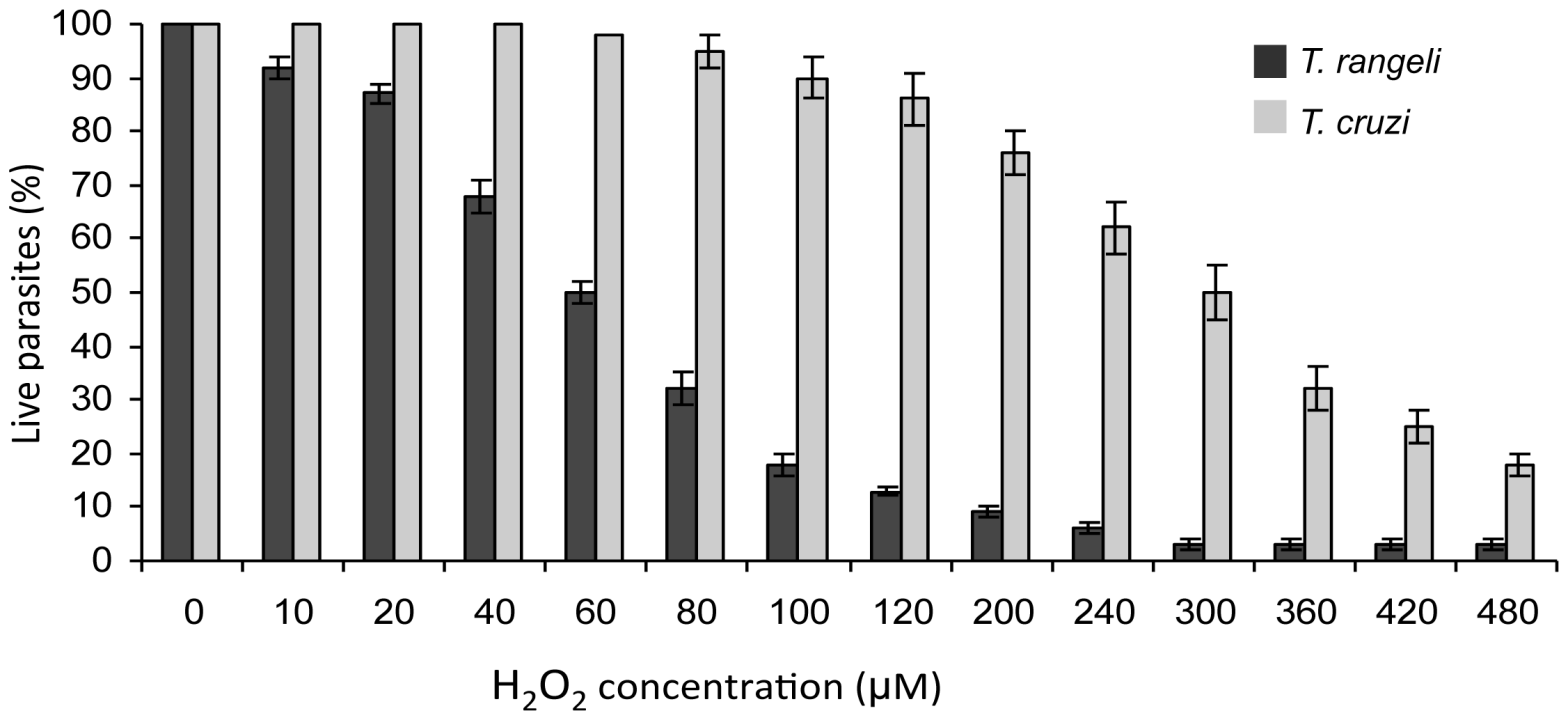
*In vitro* tolerance to hydrogen peroxide is significantly lower in *Trypanosoma rangeli* than *T. cruzi*. Epimatigote forms were cultured for 3 days in the presence of different concentrations of hydrogen peroxide, and the percentages of live parasites were determined using a model Z1 Coulter Counter. Mean values ± standard deviations from three independent experiments conducted in triplicate are indicated.

Several antioxidant enzymes work sequentially in different sub-cellular compartments to promote hydroperoxide detoxification (Table S7) [123]. During its life cycle, *T. rangeli* is exposed to reactive oxygen species (ROS) in its triatomine vectors and possibly in its mammalian host. ROS are generated through oxidative metabolism and oxidative bursts in the host immune system [124]. Interestingly, epimastigotes of *T. rangeli* (SC-58 strain) are 5-fold more sensitive to hydrogen peroxide (H_2_O_2_) than *T. cruzi* (Y strain) forms, with IC50 values of 60 µM±2 and 300 µM±5, respectively (Figure 7). It has been reported that the membrane-bound phosphatases of *T. rangeli* are more sensitive to the addition of sublethal doses of H_2_O_2_ than *T. cruzi* phosphatases [125].

In trypanosomatids, the major antioxidant molecule is a low molecular weight thiol trypanothione, which maintains the intracellular environment in a reduced state, essentially through the action of trypanothione reductase [126]. Trypanothione is a conjugate formed in two-steps via the bifunctional enzyme trypanothione synthetase (TRS) using two glutathione molecules and one spermidine. Two genes coding to trypanothione synthetase, and one to trypanothione synthetase-like are present in *T. rangeli*. Considering the substrates, glutathione synthesis is observed in *T. rangeli*, as in all trypanosomatids, despite the absence of *de novo* cysteine biosynthesis [127]. However, while in *T. brucei*, *Angomonas fasciculata* and *Leishmania* spp., the spermidine is synthesized from ornithine and methionine; in *T. cruzi*, the key enzyme ornithine decarboxylase (ODC) is absent, and the parasite solely depends on polyamine uptake by transporters to synthesize trypanothione. The *odc* gene is not present in *T. rangeli*, suggesting that this parasite also requires exogenous polyamines [128].

Trypanothione reductase (TR), a key enzyme involved in antioxidant defense in trypanosomatids, is present in *T. rangeli* and shares 84% identity with the *T. cruzi* enzyme at the amino acid level. Trypanothione is maintained in its reduced form (T-SH_2_) by the action of trypanothione reductase and the cofactor NADPH [126]. The reactions of the trypanothione cycle are catalyzed by tryparedoxin peroxidase (TXNPx) and ascorbate peroxidase (APX), which are responsible for the subsequent detoxification of H_2_O_2_ to water [126]. These enzymes use tryparedoxin and ascorbate as electron donors, respectively, which are in turn, reduced by dihydrotrypanothione.

As with other trypanosomatids, *T. rangeli* produces superoxide dismutase (SOD), an enzyme that removes excess superoxide radicals by converting them to oxygen and H_2_O_2_ [129]. Three *Fe-sod* genes were found in *T. rangeli*: *Fe-sod-a*, *Fe-sod-b* and a putative *Fe-sod*, sharing 90%, 88% and 84% identity with *T. cruzi* Fe-*sod* genes, respectively. Additionally, as with to *T. cruzi*, *T. rangeli* exhibits genes encoding distinct TXNPx proteins, including one cytosolic, one mitochondrial and one putative TXNPx sequence. Both enzymes possess two domains that are common to subgroup 2-Cys, and is present in antioxidant enzymes from the peroxiredoxin family [130]. The *T. rangeli* genome also contains two glutathione peroxidases (*gpx*), which act as antioxidants by reducing H_2_O_2_ or hydroperoxides with a high catalytic efficiency in different cellular locations [128]. In addition, enzymes related to sensitivity of nifurtimox or benzonidazol were identified in *T. rangeli*, including nitroreductase and prostaglandin F2 synthetase.

An ortholog of the ascorbate peroxidase gene from *T. cruzi* (*apx*) is present as a pseudogene in *T. rangeli*, as it exhibits a premature stop codon or frame shifts. Interestingly, this enzyme, which is a class I heme-containing enzyme, is present in photosynthetic microorganisms, plants and some trypanosomatids, such as *Leishmania* spp. and *T. cruzi*, but is absent in *T. brucei* [131]–[133]. In *T. cruzi*, ascorbate peroxidase and glutathione-dependent peroxidase II metabolize H_2_O_2_ and lipid hydroperoxides in the endoplasmic reticulum. It can be speculated that the higher sensibility of *T. rangeli* to H_2_O_2_ compared to *T. cruzi* could be related to the absence of ascorbate peroxidase activity. Proteomic analyses conducted in *T. cruzi* have demonstrated upregulation of components of the parasite antioxidant network during metacyclogenesis, including TcAPX, reinforcing the importance of the antioxidant enzymes for successful infection [134], [135]. Wilkinson et al. [136] suggested that *T. brucei* may not require ascorbate-based antioxidant protection because, as an extracellular parasite, it is not exposed to the oxidative challenge from host immune cells produced in response to intracellular infection of *T. cruzi* or *Leishmania* spp. Thus, the limited capability of *T. rangeli* to respond to oxidative stress could be related to the inability of this parasite to infect and multiply inside vertebrate host cells. This observation may suggest a distinct replication site for this parasite in the mammalian host, similar to the extracellular cycle of *T. brucei*.

In Table S8, the genes encoding the stress response proteins of *T. rangeli* are presented. A large set of heat shock protein genes is found in the genome of this parasite, occasionally displaying a reduced copy number compared with *T. cruzi*. Similarly to *T. cruzi*, the *T. rangeli* genome contains 17 *hsp70* genes, 13 of which are cytosolic, while 3 are mitochondrial, and one localized to the endoplasmic reticulum. On the other hand, only one *hsp85* and *hsp20* genes were found in the *T. rangeli* genome, compared to 6 and 11 copies in *T. cruzi*, respectively. The large number of *hsp40* genes observed in kinetoplastids (68 copies in *T. cruzi*) [137] is also reduced in *T. rangeli* (24 copies).

Thus, where the reduced repertoire of transialidases and MASPs may correlate with diminished ability to enter mammalian cells, it can be speculated that the reduced number of genes related to different cellular stress responses provides for a more limited capability of *T. rangeli* to respond to oxidative stress and that this in turn corresponds with an apparent inability to survive and multiply within mammalian cells.

### Conclusions

At 24 Mb (haploid), the *T. rangeli* genome is the shortest and least variable genome from the mammalian-infective trypanosomatids to date. Our elucidation of its sequence both answers and poses a variety of intriguing questions about the biology of a trypanosome which is infectious but non-pathogenic to humans and which is carried by triatomine bugs and sympatrically distributed with *T. cruzi*, but which shows a salivarian rather than a stercorian route for infection. Based on phylogenomic analysis, *T. rangeli* is undoubtedly positioned as a stercorarian parasite, chromosome structure and progressive loss of RNAi machinery in this lineage lend support to this interpretation and the results presented here corroborate previous results based on distinct nuclear and mitochondrial markers. The different evolutionary path of this trypanosome species is, though, writ large on its genome by a differential in the preponderance of gene duplication and divergence, particularly at the telomeres, with reduced diversity in genes known to be associated with infection of the mammalian host such as transsialidases, MASPs and oxidative stress and rather more diversity in other non-telomeric gene families such as KMP-11s and amastins which may imply roles for these families in vector interactions. It is interesting to consider to what extent the *T. rangeli*-*Rhodnius* vector species co-evolution of salivary gland colonization (and anterior transmission) is an example of parallel or convergent evolution with the colonization of the tsetse salivary gland by African trypanosomes, and to what extent the apparatus for this phenotype was already present in a progenitor. Our release of the *T. rangeli* genome casts further light on the evolutionary origins and relationships of trypanosomes, and provides a resource for better understanding the function of genes and factors related to the virulence and pathogenesis of trypanosomiasis and with which to address unknown aspects of the *T. rangeli* life cycle in mammalian hosts.

## Supporting Information

Figure S1 **Mapping of** ***T. rangeli*** **sialidase sequences on a multidimensional scaling (MDS) plot of** ***T. cruzi*** **TcS protein sequences.** The MDS shows the pattern of dispersion of the *T. cruzi* TcS sequences, as proposed by [82]. All individual *T. rangeli* reads were searched against the *T. cruzi* predicted proteome using the *BLASTx* algorithm, and all reads whose best hits were against *T. cruzi* TcS genes were retained. TcS genes showing at least 50% coverage with *T. rangeli* sialidase genes are displayed as black dots. TcS groupI - blue; TcS groupII - dark green; TcS groupIII - light blue; TcS groupIV - magenta; TcS groupV - red; TcS groupVI - gray; TcS groupVII - orange and TcS groupVIII - purple.
(TIF)

Figure S2 **Schematic representation of the** ***T. rangeli*** **maxicircle.** Colored arrows represent the orientation of each maxicircle gene. ND indicates NADH dehydrogenase genes; Cyb indicates cytochrome B; COI/COII indicates cytochrome c oxidase. Numbers are in base pairs.
(TIF)

Figure S3 **Schematic representation of the comparative analysis of the ends of the assembled scaffolds from the** ***T. rangeli*** **genome** and previously reported telomere sequences [97].
(TIF)

Figure S4 **Alignment of** ***ago1*** **,** ***dcl1*** **,** ***rif4*** **, and** ***rif5*** **pseudogenes from** ***T. rangeli*** **Choachí and SC-58.**
(PDF)

Figure S5 **Conservation of DCL-2 in RNAi-positive trypanosomes and** ***T. rangeli*** **.** Panel **A** shows a multiple alignment of potential DCL2 proteins from *T. b. gambiense*, *T. b. brucei*, *T. congolense* and *T. rangeli* generated by MultiAlin. Amino acids in red are conserved in all sequences. Panel **B** summarizes the identity shared by the potential DCL2 proteins. The lysine and glutamic acid residues highlighted in green are part of the RNaseIII domain of DICERs, which have been shown to be important for the catalytic activity of TbDCL2 [110].
(PDF)

Table S1 **Comparison of satellite DNA found in** ***T. rangeli*** **strain SC-58 genomic and transcriptomic libraries with the** ***T. cruzi*** **haploid genome (CL Brener strain).**
(DOCX)

Table S2 **Comparative distribution of microsatellites found in** ***T. rangeli*** **genomic (G) and transcriptomic (T) datasets.**
(XLSX)

Table S3 **Comparative number of translation process-related proteins from distinct kinetoplastid species.**
(DOC)

Table S4 ***Trypanosoma rangeli*** **ePKs with predicted transmembrane domains.**
(DOCX)

Table S5 **Phosphatidylinositol and related kinase proteins identified from the predicted proteomes of** ***T. rangeli*** **and** ***T. cruzi*** **.**
(DOCX)

Table S6 **Accessory domains present in PIK-related proteins in** ***T. rangeli*** **and** ***T. cruzi*** **(Model 5).**
(DOCX)

Table S7 **Antioxidant enzymes of trypanosomatids.**
(DOCX)

Table S8 **The stress response proteins of** ***T. rangeli*** **.**
(DOC)
