# Supplementary figures and images for: Genome of the Avirulent Human-Infective Trypanosome—Trypanosoma rangeli
Source: PLoS Negl Trop Dis. 2014 Sep 18;8(9):e3176. doi: 10.1371/journal.pntd.0003176 (PMC4169256; doi:10.1371/journal.pntd.0003176)

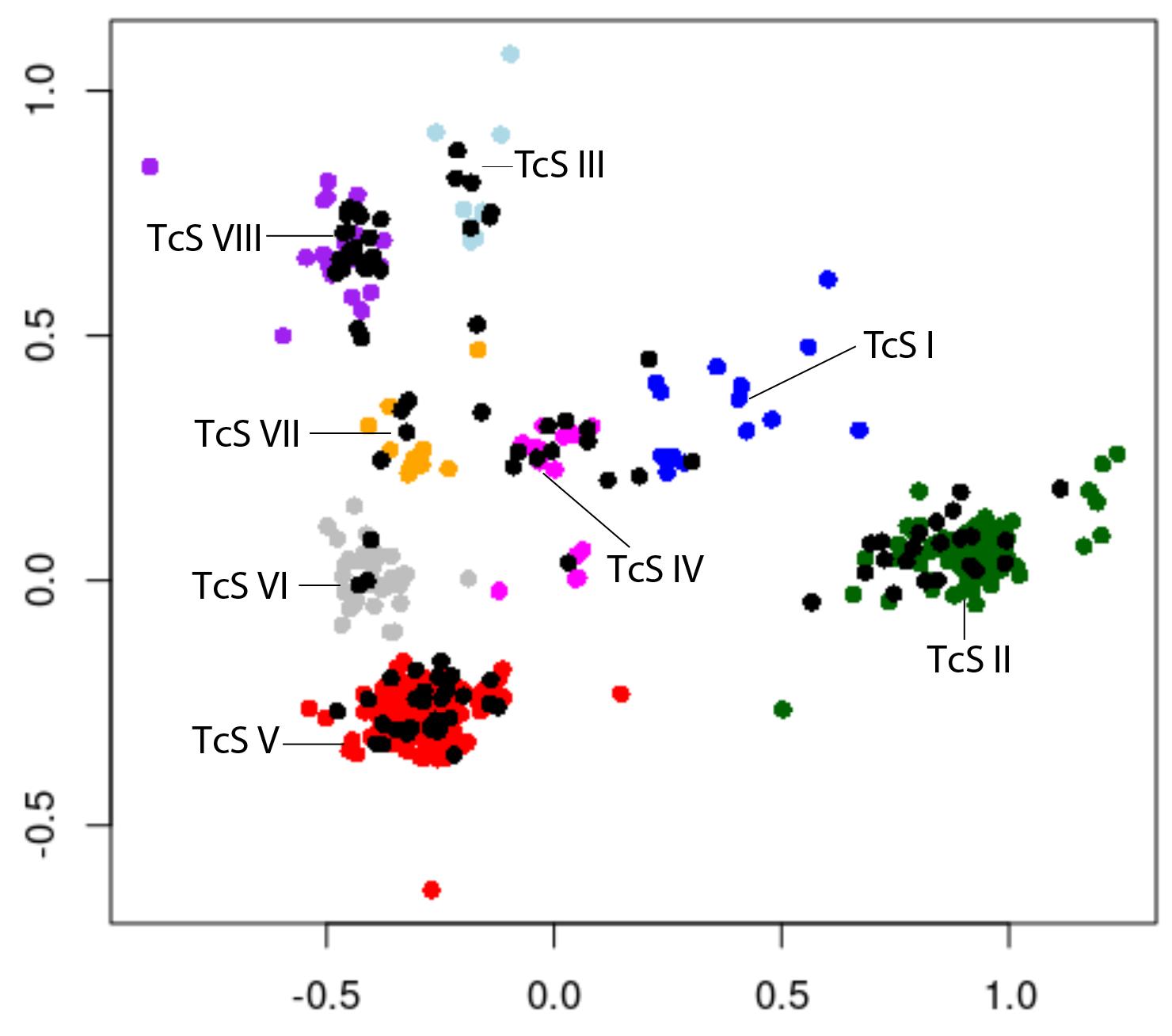

Supplement: Figure S1 — Mapping of T. rangeli sialidase sequences on a multidimensional scaling (MDS) plot of T. cruzi TcS protein sequences. The MDS shows the pattern of dispersion of the T. cruzi TcS sequences, as proposed by [82]. All individual T. rangeli reads were searched against the T. cruzi predicted proteome using the BLASTx algorithm, and all reads whose best hits were against T. cruzi TcS genes were retained. TcS genes showing at least 50% coverage with T. rangeli sialidase genes are displayed as black dots. TcS groupI - blue; TcS groupII - dark green; TcS groupIII - light blue; TcS groupIV - magenta; TcS groupV - red; TcS groupVI - gray; TcS groupVII - orange and TcS groupVIII - purple. (TIF) [file pntd.0003176.s001.tif]

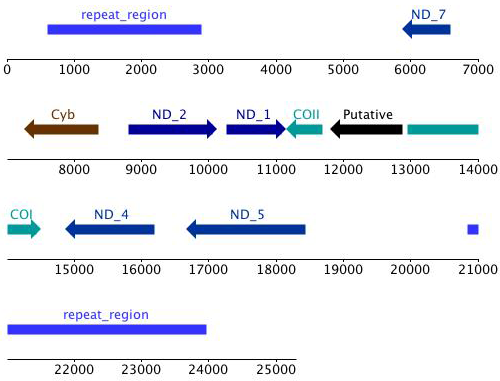

Supplement: Figure S2 — Schematic representation of the T. rangeli maxicircle. Colored arrows represent the orientation of each maxicircle gene. ND indicates NADH dehydrogenase genes; Cyb indicates cytochrome B; COI/COII indicates cytochrome c oxidase. Numbers are in base pairs. (TIF) [file pntd.0003176.s002.tif]

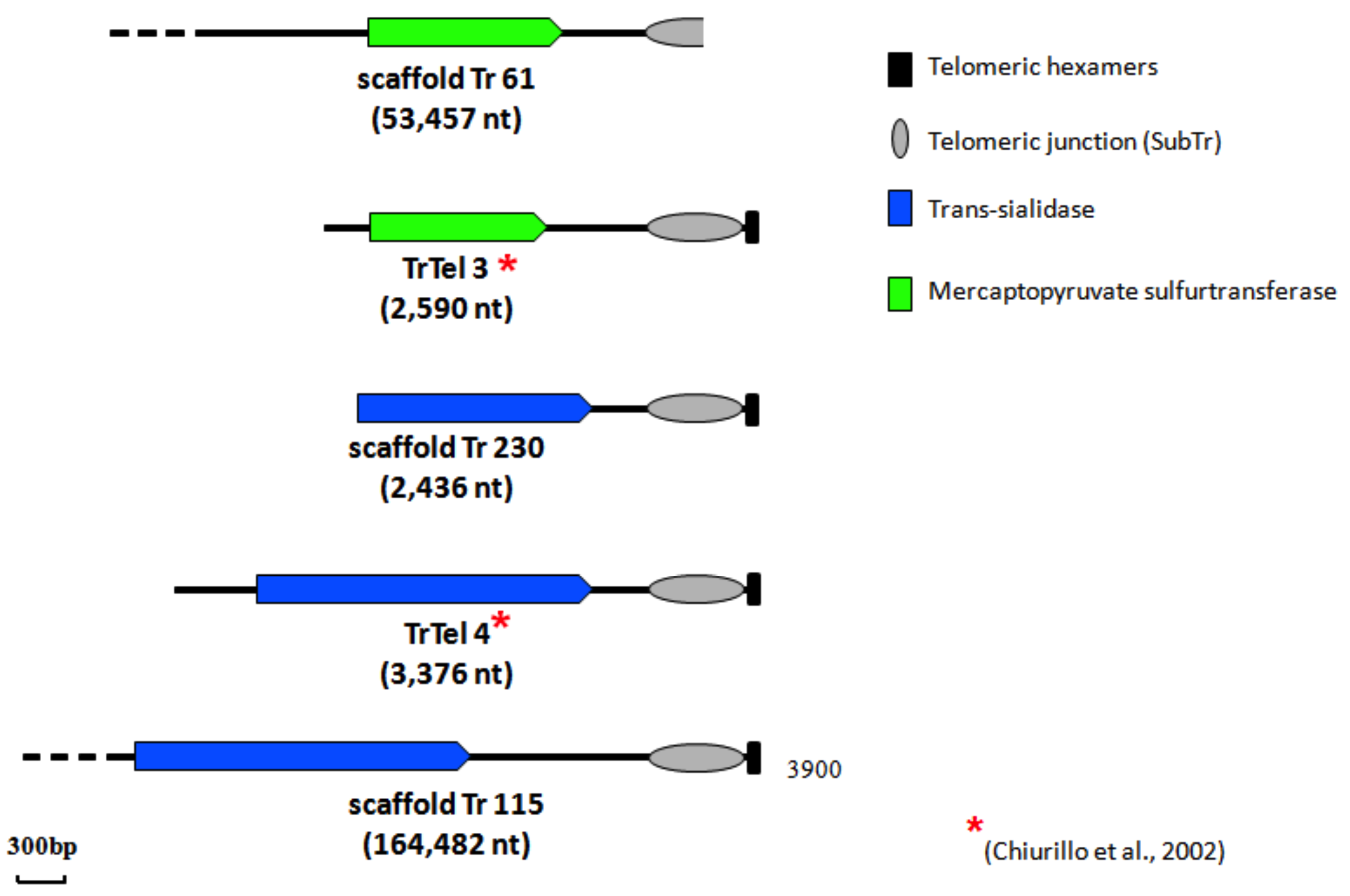

Supplement: Figure S3 — Schematic representation of the comparative analysis of the ends of the assembled scaffolds from the T. rangeli genome and previously reported telomere sequences [97]. (TIF) [file pntd.0003176.s003.tif]
