# Supplementary material for: Genome of the Avirulent Human-Infective Trypanosome—Trypanosoma rangeli
Source: PLoS Negl Trop Dis. 2014 Sep 18;8(9):e3176. doi: 10.1371/journal.pntd.0003176 (PMC4169256; doi:10.1371/journal.pntd.0003176)
Supplement: Figure S4 — Alignment of ago1 , dcl1 , rif4 , and rif5 pseudogenes from T. rangeli Choachí and SC-58. (PDF) [file pntd.0003176.s004.pdf]

```
TrAgo1_Choachí      TCAGGAAGAGGGAGAGGTTGGCGAGGAAGAGGCGGTGGCGAATCCCATGAATATCGTGGT 60
TrAgo1_SC58         TCAGGAAGAGGGAGAGGTTGGCGAGGAGGAGGCGGTGGCGAATCCCATAAATATCGTGGT 60
                    *************************** ******************** ***********

TrAgo1_Choachí      AGAGGCGGACGTGGTGGCGGAAGAGGTTATGGCGATTTTGGCGGTCGTGGTCGTGG---- 116
TrAgo1_SC58         AGAGGCGGACGTGGTGGCGGAAGAGGTTATGGCGATTTTGGCGGTCGTGGTCGTGGTCGT 120
                    ********************************************************

TrAgo1_Choachí      --CGGTGGCGTCCGCTTCCAGAATCAAGAGCGAGAGAAGACAGTCATCGTTGACTTCGCC 174
TrAgo1_SC58         GGCGGTGGCGTCCGCTTCCAGAATCAAGAGCGAGAGAAGACGGCCATCGTTGACTTCGCC 180
                      *************************************** * ****************

TrAgo1_Choachí      AGGGATAAAAGTACAACTGCCCCCTGGAGGCCATCTGTAACATGATACCGCTCAAGTTT 234
TrAgo1_SC58         AGGGAGAAAAGTACAACTGCCCCCTGGAGGCCATCTGTAACATGGTACCGCTCAAGTTT 240
                    ***** ************************************** ***************

TrAgo1_Choachí      GACCCAAAGAAAAAGGTGTTTGCCTATATTTTGTTGTATGAACTGAATGATGGCGCTCCG 294
TrAgo1_SC58         GACCCAAAGAAAAAGGTGTTTGCCTATATTTTGTCGTATGAACTGAGTGATGGCGCTCCG 300
                    ********************************** *********** *************

TrAgo1_Choachí      AATTTGCAGGATGGCCTGAAAGAACGGGCTTCACAGGAAATGCTACGAAAGATGCAAAAA 354
TrAgo1_SC58         AATTTGCAGGATGGCCTGAAAGAACGGGCTTCACAGGAAATGCTACGAAAGATGCAAAAA 360
                    ************************************************************

TrAgo1_Choachí      GAAAGCCCGGAAGGCAAAAGTTTTGACGTTGATTTGTGCGTTTGCACGGGCCAAAGTATT 414
TrAgo1_SC58         GAAAGTCCGGAGGTCAAAAGTTTTGACGTTGATTTGTGCGTTTGCACGGGCCAAAGTATT 420
                    ***** ***** * **********************************************

TrAgo1_Choachí      TTGTCCCCACAACGACTCCCCATCGAGGAGTTTAGTCATGAGTTCACCGTGAAATCAAGA 474
TrAgo1_SC58         TTGTCCCCACAACGACTCCCCATCGAGGAGTTTAGTCATGAGTTCACCGTGAAATCAAGA 480
                    ************************************************************

TrAgo1_Choachí      AAGGGTAACTGTATCTACTCCTATGTCCTCACCCTTGCGAGACCAGAGATCACCGCACTC 534
TrAgo1_SC58         AAGGGTAACCGTATCTGCTCCTATGTCCTCACCCTTGCGAGACCAGAGATCATCGCACTC 540
                    ********* ****** *********************************** *******

TrAgo1_Choachí      AACGGCGAAGAGTACCGAATGGAGGTGAACCAGCATAGTTGGGAAGGCTGTACAAGTCTG 594
TrAgo1_SC58         AACGGCGAAGAGTTCCGAATGGAGGTGAAC-AGCATAGTTGGGAAGGCTGTACAGGTCTG 599
                    ************* **************** *********************** *****

TrAgo1_Choachí      CTATGGAGAAAAGATGGGTGGAAAGTACGTCGATATTCAGGCGGGCACAGCGGCTCCTGG 654
TrAgo1_SC58         CTATGGAGAAAAGATGGGTGGAAAGTACGTCGATATTCAGGCGGGCACAGCGGCTCCTGG 659
                    ************************************************************

TrAgo1_Choachí      AGGACACATTGCCACCTTTGACTGCGTTGTGCCAAAGGTGCTGACTACCACAGTGAATGG 714
TrAgo1_SC58         AGGACACATTGCCACCTTTGACTGCGTTGTGCCAAAGGTGCTGACTACCACAGTGAATGG 719
                    ************************************************************

TrAgo1_Choachí      GGTGCAGATGGCACTGTTGCAGATCGACGCGTTGGTCTCTGTTGCGTCAGCGCAAAATTG 774
TrAgo1_SC58         GGTGCAGATGGCACTGTTGCAGATCGACGCGTTGGTCTCTGTTGCGTCGGCGCAAAATTG 779
                    ************************************************ ***********

TrAgo1_Choachí      TCTCGAAATTCTCGAAGGTACAAGGCGACATGAACGCCGAAACGTCAACAGGGTACTTAT 834
TrAgo1_SC58         TCTCGAAATTCTCGAAGATATAAGGCGACGTGAACGCCGAAACGTCAACAGGGTACTTAT 839
                    ***************** ** ******** ******************************

TrAgo1_Choachí      GGAGCGCTTTTCAAAAAAGAAAAAA-----AAGTGTGCACCATCCTTCATGGTCACCGCG 889
TrAgo1_SC58         GGAGCGCTTTTCAAAAAAAGAAAAAGAAAAAAGTGTGCACCATCCTTCATGGTCACCGCG 899
                    ******************  *****     ******************************

TrAgo1_Choachí      GAGCGGTTTACACCGTTCTTGATATCTCGGACAAAAAAGCTAAAGATCCTGCCGAATTGA 949
TrAgo1_SC58         GAGCGGTTTACACCGTTCTTGATATCTCGGACAAAGAAGCTAAAGATCCTGCCGAATTGA 959
                    *********************************** ************************

TrAgo1_Choachí      GGAAAATCCTGAGCTGACATTTGTGCAGTACTTTGCGCAAAAATACGGTATACATCTCAT 1009
TrAgo1_SC58         GGAAAA------------------------------------ATACGGTATACATCTCAT 983
                    ******                                    ******************

TrAgo1_Choachí      TCCTGAACAGATACTGTTCAGGTGCCGTACCAGTTCGGGGAAGAAGGTACTTGTGCCGCC 1069
TrAgo1_SC58         GCCTGAGCAGATATTGTTCAGGTGCCGTACCAGTTCGGGGAAGAAGGTACTTGTGCCGCC 1043
                     ***** ****** **********************************************

TrAgo1_Choachí      GGAAGTCTTGTATGAGATGTCCCTCAGTGAACGAGACCGGTGGCAGCCGCCGCAGCTGTG 1129
TrAgo1_SC58         AGAAGTCTTGAATGGGATGTCCCTCAGTGAACGAGACCGGCGGCAGCTGCCGCAGCTCTG 1103
                     ********* *** ************************* ****** ********* **

TrAgo1_Choachí      TTTGCTGTACCCAAATGAACGCATGGAACGGGTCAAAAAAGCGATTGAACGCCTTAAACA 1189
TrAgo1_SC58         TTCGCTGTACCCAAATGAACGCATGGAACGGGTCAAAAAAGCGATTGAACGCCTTAAACA 1163
                    ** *********************************************************

TrAgo1_Choachí      TGAGAATGACGCCAAAGCCATGAAAATTCTAAATTCATTTGGTATTATTATTGGAAACAA 1249
TrAgo1_SC58         TGAGAATGACGCCAAAGCCATGAAAATTCTAAATTCATTTGGTATTATTATTGGAAACAA 1223
                    ************************************************************

TrAgo1_Choachí      CTTTTTGAAAGTTTCTGGAAATGTGCTGCCACCCATTCAGGTGCTTGTTCCACAAGCAAA 1309
TrAgo1_SC58         CTTTTTGAAAGTTTCTGGAAATGTGCTGCCACCCATTCAGGTGCTTGTTCCACAAGCAAA 1283
                    ************************************************************

TrAgo1_Choachí      CGGGTACAAAACTGTCGATATTCTTGAGGAAAACACCCAGCAAGGCTTTGCTCGGGAGCT 1369
TrAgo1_SC58         CGGGTACAAAACTGTCGATACTCTTGGGGAAAACACCCAGCAAGGCTTTGTTCGGGAGCT 1343
                    ******************** ***** *********************** *********

TrAgo1_Choachí      CGGGGGATTACGGCATTCGGGCAACCGAAAAGCCATTGGGGTTGACTTGTACGACGAATC 1429
TrAgo1_SC58         CGGGGGATTACGGCATTCGGGCAACCAAAAAGCCATTGGTGTTGTCTTGTACGACGCATC 1403
                    ************************** ************ **** *********** ***

TrAgo1_Choachí      GCAACAGGGGAAGAAGGCACTGGATGCGATTAAGAATCATTTGGCAAAGATGAACGCCGC 1489
TrAgo1_SC58         GCAACAGGGGGAGAAGGCACTGGATGCGATTAAGAATCATTTGGCAAAGATGAACGCCGT 1463
                    ********** ************************************************

TrAgo1_Choachí      CGTGACGCCAGCATGGGTGCAGCATGCGGGAAGCCTAGATGACGTCAAAAAACTTGCGTC 1549
TrAgo1_SC58         TGTGATGCCAGCATGGGTGCAGCATGCAGGAAGCCTGGACGACGTCAAAAAACATGCGTC 1523
                     **** ********************* ******** ** ************* ******

TrAgo1_Choachí      CAAGAACGCGTTTGCTTTTGCTTTTCTGCGCCACTTTGATAAAGAGTTGTACAGAAGTTT 1609
TrAgo1_SC58         CGAGAACGCGTTTGCTTTTGCTTTTCTGCGCCACTTTGATAAAGAGTCGTACAGAAGTTT 1583
                    * ********************************************* ************
```

```
TrAgo1_Choachí      GAAGACAACGTGGACAAATAAGGACATCTTAAGTCAAGTGGTGGTAAAGGATTTGACTGG 1669
TrAgo1_SC58         GAGGACAACGTGGACAAATAAGGACATCTTAAGTCAAGTGGTGGTAAAGGATTTGACTGG 1643
                    ** *********************************************************

TrAgo1_Choachí      GTCCCGTGAAACACCAACTGTCATGGCAGTGGCGCAGCAGGTGTGCGCCAAAGGTGGCCA 1729
TrAgo1_SC58         GCCCCGTGAAACACCAATTGTCATGGCAGTGGCGCAGCAGGTGTGCGCCAAAGGTGGCCA 1703
                    * *************** ******************************************

TrAgo1_Choachí      GCTGAATTGGGTGCTGGACATCAATAAATTGTGCCCGTCACTTGCCTCCAAAATTGGATC 1789
TrAgo1_SC58         GCTGAATTGGGTGCTGGACATCAATAAATTGTGCCCGTCATTTGCCCCCAAAATTGGATC 1763
                    **************************************** ***** *************

TrAgo1_Choachí      CGCAGGAGTATTGATCATTGGAGCCGATATAGGGAGAGATCAGAGGGAGCTGCGTACCGA 1849
TrAgo1_SC58         CGCAGGAGTATTGACCATTGGAGCCGATATAGGGAGAAATCAGAGGGAGCTGCGTACCGA 1823
                    ************** ********************** **********************

TrAgo1_Choachí      CGAATCCACGGTCGAACAAGAAATTTATACGGTGGCCTTTGTGGCCTTCCATGCTTGTGG 1909
TrAgo1_SC58         CGAATCCACGGTCGAACAAGAAATTTATACGGTGGCCTTTGTGGCCTTCCATGCTTGTGG 1883
                    ************************************************************

TrAgo1_Choachí      AACACGATGGGAAACTTACTGCGACCACTACCACGTGGATGGCCGCAAGCGAAGTTTATT 1969
TrAgo1_SC58         AACACGATGGGAAACTTACTGCGACCATTACCACGTGGATGGCCGCAAGCGAAGTTTATT 1943
                    *************************** ********************************

TrAgo1_Choachí      CGCTCAACGCCCGCAAGCAGAGGGCTCAAGTGTCTCGGAAAAAGCCTTCATGCCCTCTGA 2029
TrAgo1_SC58         CGCTCAAAGCCCGCAAGCAGAGGGCTCAAGTGTCTCGGAAAAAGCCTTCATGCCCTCTGA 2003
                    ******* ****************************************************

TrAgo1_Choachí      GGTGCTCTCAGGAAAGCTGAGAGGCTTCTTGGACGAGGCGAGGCGCCACTTTTTGGACAA 2089
TrAgo1_SC58         GGTGCTCTCATGAAAGCTGAGAGGCTTCTTGGACGAGGCGAGGCGCCACTTTTTGGACAA 2063
                    ********** *************************************************

TrAgo1_Choachí      AGGTTTGCAAACTTCTGCGGTGCTGCTGCTTCGTGGCTGTGCGTCCGAGGGTGAGCTGTT 2149
TrAgo1_SC58         AGGTTTGCAAACTTCTGCGGTGCTGCTGCTTCGTGGCTGTGCATCCGAGGGTGAGCTGTT 2123
                    ****************************************** *****************

TrAgo1_Choachí      GGCCGCGAAAGAGCACGACGTGGACCTGCTAAGTGGGTCCTTAAAA-----CGTCCTGTG 2204
TrAgo1_SC58         GGCCGCGAAAGAGCACGACGTGGACCTGCTAAGTGGGTCCTTAAAAGAAAACGTCCTGTG 2183
                    **********************************************     *********

TrAgo1_Choachí      GGGAGTGGTGGCGTCGCAGCGCTACCAGCATACGCGCTTTGCCGCCGCCACACCTAAGGA 2264
TrAgo1_SC58         GGGAGTGGTGGCGTCGCAGCGCTACCAACATACACGCTTTGCCCCCGCCACACCTAAGGA 2243
                    *************************** ***** ********* ****************

TrAgo1_Choachí      GCCCGACACGCTATGCAATGTTCCACGCGGCTTTGTGACGCGTGAGGGGACCGACTCGAA 2324
TrAgo1_SC58         GCCCGACACGCCATGCAATGTTCCACGCGGCTTTGTGACGCGTGAGGGGACAGACTCGAA 2303
                    *********** *************************************** ********

TrAgo1_Choachí      ATTTGGCGAGTTATTTTTTTTTAACGGTGCGAACTGCACGCTTGGCCACGCGCGCGCCAC 2384
TrAgo1_SC58         ATTTGGCGAGTCATTTTTTCTTAACGGTGCGAACTGCACGCTTGGCCACGCGCGCGCCGC 2363
                    *********** ******* ************************************** *

TrAgo1_Choachí      GCTGTACGTCGTGTTGGGGAGGAGAAAAAGCTTTTTGCTGGAGGAGATTCAGGTACTGCT 2444
TrAgo1_SC58         GCTGTACGTCGTGTTGGGGAGGAAAAAAAGCTTTTTGCTGGAGGAGATTCAGGTACTGCT 2423
                    *********************** ************************************

TrAgo1_Choachí      GCATGGGATGTGTTTCCTGTACCCGAACAAGGCGGATGGCCTCCCGTTGCCACTGCCGCT 2504
TrAgo1_SC58         GTATGGGATGTGTTTCCTGTACCCGAACAAGGCGGATGGCCTTCCGTTGCCACTGCCGCT 2483
                    * **************************************** *****************

TrAgo1_Choachí      GAAGTGCGCGTCGGAGTACTCACGGAAGTTTGTGTCTTTGCGCGGTGTCAAAAAGCTTTC 2564
TrAgo1_SC58         GAAGTGCGCGTCGGAGTACTCACGGAAGCTTGTGTCTTTGCGCGGTGTCAAAAAGCTTTC 2543
                    **************************** *******************************

TrAgo1_Choachí      CAAGGAGCTGCGCGGCAGGCTGCACTACCTGTAGTTGGCCGCCTCTCTGCTGGCACGAAC 2624
TrAgo1_SC58         CAAGGAGCTGCGCGGCAGGCTGCACTACCTGTAGTTGGCCGCCTCTCTGCTGGCACGAAC 2603
                    ************************************************************

TrAgo1_Choachí      GGCCAAGTGCATCTGCCACAAACGGCGTTGTCCAGGAGTGCGTGACACGGGGCGGACGGC 2684
TrAgo1_SC58         GGCCAAATGCATCTGCCACGAACGGCGTTGTCCGGGAGTGCGTGACACGGGGCGGACGGC 2663
                    ****** ************ ************* **************************

TrAgo1_Choachí      GCCAAGGTGTGTGCGTGTGTAATG 2708
TrAgo1_SC58         GCCAAGGTGTGTGCGTGTGTA--- 2684
                    *********************
```

```
TrDcl1_Choachí      ----TATTTCAGTTTGGGTGGGACATTTTTTTATTTTATTTTTTGCTCTTCCAAGGACTT 56
TrDcl1_SC58         CGATTATTTCAGTTTGGGTGGGACATTTTTTTGTTTTATTTTTTGCTCTTCCAAGGACTT 60
                        **************************** ***************************

TrDcl1_Choachí      TTTCAACATGCATGGGGGCGGTGATGGCGGGGTCACTGTGGGGGAGTTGCTGCGGTTGTG 116
TrDcl1_SC58         TTTCAACGTGCATGGGGGCGGTGATGGCGGGGTCACTGTGGGGGAGTTGCTGCGGTTGTG 120
                    ******* ****************************************************

TrDcl1_Choachí      GATGTCGCCGACGACGAGGCTGATGGACGCCCTTGAGAATGTTGGGACGCTGATAGGGAT 176
TrDcl1_SC58         GATGTCGCCGACGACGAGGCTGGTGGACGCCCTTGAGAATGTTGGGACGCTGATAGGGAT 180
                    ********************** *************************************

TrDcl1_Choachí      GAAGGAGCTGCGGGAAATGCTGGGGTCTGTTCCGCCGCATGCTGAGGCACTCAAAAGGGA 236
TrDcl1_SC58         GGAGGAGCTGCGGGGAATGCTGGGGTCTGTTCCGCCGCATGCTGAAGCACTCAAAAGGGA 240
                    * ************ ****************************** **************

TrDcl1_Choachí      GGGATCCGGCGTCAGTGCGGCCTCACTGGCGGGGGAGAAGCTCTTGAAGACGCTTGCGTC 296
TrDcl1_SC58         GGGATCCGGCGTCAGTGCGGCCTCACTGGCGGGGGAGAAGCTCTTGAAGGCGCTTGCGTC 300
                    ************************************************* **********

TrDcl1_Choachí      CGAATACGCCTTCCTTTTATTTCCCAAGTGAGGCACCGCTAAGCTTACCAACGTAACGCG 356
TrDcl1_SC58         CGAATACGCCTTCCTTTTATTTCCCAAGGGAGACACCGCTAAGCTTACCAACGTAACGCG 360
                    **************************** *** ***************************

TrDcl1_Choachí      TGTACTCCTCTCCTCTGACACGCTTGACCCGGAGTTGGAAAAGTTCCTCGGCACCAAGTT 416
TrDcl1_SC58         TGTACTCCTCTCCTCTGACACGCTTGACCCGGAGTTGGAGAAGTTCCTCGGCACCAAGTT 420
                    *************************************** ********************

TrDcl1_Choachí      GGCGGCACGCAAGGTGTTCCTGCCTTTTTTGGCGCTGCTGGTGTGGGCGCGGAGCATTCC 476
TrDcl1_SC58         GGCGGCACGCAAGGTGTTCCTGCCTTTTTTGGCGCTGCTGGTGTGGGCGCGGGGCATTCC 480
                    **************************************************** *******

TrDcl1_Choachí      TGCAGCGCGTTCCCTCCAACTTCCCGTGCTGCGCAGTGTGATGAGCACCGGGGGCTACGC 536
TrDcl1_SC58         TGCAGCGCGTTCCCTCCTACTTCCCGTGCTGCGCAGTGTGATGAGCACCAGGGGCTACGC 540
                    ***************** ******************************* **********

TrDcl1_Choachí      GGTGCCATTGCCAGCCATTTTGTCCTCGCTGTGCCCCAGGGAGTCGTTTGGTTGGCTTTC 596
TrDcl1_SC58         GGTGTCATTGCCAGCCATTTTGTCCTCGCTGTGTCCCAGGGAGTCGTTTGGTTGGCTTTC 600
                    **** **************************** **************************

TrDcl1_Choachí      ACATGAGCAGCAGATTGAGTTGATGGTGAAGGAACTGGAACAGCGCGGAGTATGTGTGTC 656
TrDcl1_SC58         ACATGAGCAGCAGATTGAGTTGATGGTGAAGGAACTGGAACAGCGCGGAGTGTGTGTGTC 660
                    *************************************************** ********

TrDcl1_Choachí      GCGGGAGGCGGAAGAGTCGAATCCAAATGAGGATGACGGGGTGCGTTACGCCGTGCGGGT 716
TrDcl1_SC58         GCGGGAGGCGGAAGAGTCGAATTCAAATGAGGATGACGGGGTGCGTTACACCGTGCGGGT 720
                    ********************** ************************** **********

TrDcl1_Choachí      GAGGTGCGGGGAGGAAGCAGAGTCGTGCCAGAAGGGGGTGACGCTTGCTGGATTCTACGC 776
TrDcl1_SC58         GAAGTGTGGGGAGGAAGCAGAGTCATGCCAGAAGGGG-CGACGCTTGCTGGATTCTACGC 779
                    ** *** ***************** ************  *********************

TrDcl1_Choachí      CATTGTCATGGGGGAGGGGAAGAAGATTGTTTCGCGTGCAAAAGTGGTCAAAAAGATGCA 836
TrDcl1_SC58         CATTGTCATGGGG-AGGGGAAGAGGATTGTTTCGCGTGCAAAAGTGGTCAAAAAGATGCA 838
                    ************* ********* ************************************

TrDcl1_Choachí      GAAAGAGCAGGTTACATTCTCCACGGCTAATATTAAAATGTGGATGCATAAGGTTCGTCA 896
TrDcl1_SC58         GAAAGAGCAGGTTAAATTCTCCATGGCTAATATTAAAATGTGGATGCATAGGGTTCGTCA 898
                    ************** ******** ************************** *********

TrDcl1_Choachí      CTTGCTGCGCTTTCATCCTTCATACAAGGACGCCCTGCCACAGGACTTTATGGCAAAGGT 956
TrDcl1_SC58         CTTGCTGCGCTTTCATCCTTCATACAAGGACGCCCTGCCACAGGACTTTATGGCAAAGGT 958
                    ************************************************************

TrDcl1_Choachí      GATTCACTGCGAGGGATGGATGCCGCTGAGCGAGATGTGTCGCATCCTGGCAGCGCAGTC 1016
TrDcl1_SC58         GATTCACTGCGAGGGATGGATGCCGCTGAGCGAGATGTGTCGCATCCTGGCAGCGCAGTC 1018
                    ************************************************************

TrDcl1_Choachí      GGCGGACGCGTGCGGGCTTGTGCCATCGCTGGATATGCTGCAGCAGCTGCTTGAGAACCA 1076
TrDcl1_SC58         GGCGGAAGCGTGCGGGCTTGTGCCATCGCTGGATATGCTGCAGCAGCTGCTTGAGAACCA 1078
                    ****** *****************************************************

TrDcl1_Choachí      CGACCACTTTCAACGGTATGAGGTGGGCGTTTGCGGGGTTGCGGGAGGCGCATTTAATGG 1136
TrDcl1_SC58         CGACCACTTTCAACGGTATGAGGTGGGCGTTTGCGGGGTTGCGGGAGGCGCATTTAATGG 1138
                    ************************************************************

TrDcl1_Choachí      TCAGTTGTGCGCCCGTGCGCGGTACGCCCACCAGGGCTTTGCATCACTATCCACACGCAT 1196
TrDcl1_SC58         TCAGTTGTGCGCCCGTGCGCGGTACGCCCACCAGGGCTTTGCATCACTGTCCACACGCAT 1198
                    ************************************************ ***********

TrDcl1_Choachí      CTTTGAGAAGTTTGTGACTGTCACAAACCCGAACGCGTGCCCCACCTACGCGTGGGTCTC 1256
TrDcl1_SC58         CTTTGAGAAGTTTGCGACTGTCACAAACCCGAACGCGTGCCCCACCTACGCGTGGGTCTC 1258
                    ************** *********************************************

TrDcl1_Choachí      CTTGGGTGAAAAGAGGCTGCTCGACCCCGGGCTTCGCAAGGGGTTGTACATACTGCACCA 1316
TrDcl1_SC58         CTTGGGTGAAAAGAGGCTGCTCGACCCCGGGCTTCGCAAGGGGTTGTACGTACTGCACCA 1318
                    ************************************************* **********

TrDcl1_Choachí      GGAAATGCGATTTGCTTTGGTGTTTCCCTCAGAATTGCTTATAGAATTTAGCCGCTACCA 1376
TrDcl1_SC58         GGAAATGCGACTTGCTTTGGTGTTTCCCTCGGAATTGCTTATAGAATTTAGCCGCTACCA 1378
                    ********** ******************* *****************************

TrDcl1_Choachí      ATTGCAAACACCACTGGAGGAACAGTACGACGAGAAGCTTGGGGTGGACAGGCCATTGCA 1436
TrDcl1_SC58         ACTGCAAACACCACTGGAGGAACAGTACGACGAGAAGCTTGGGGTGGACAGGCCATTGCA 1438
                    * **********************************************************

TrDcl1_Choachí      CTTTTTGGAGCTTTGTCTTTGTGCGGCAGTGCGGGATGCCGAGATGACAGTGCTCCAGGC 1496
TrDcl1_SC58         CTTTTTGGAGCTTTGTCTTTGTGCGGCAGTGCGGGATGCCGAGATGACAGTGCTCCAGGC 1498
                    ************************************************************

TrDcl1_Choachí      CCCACCGCGATGGGGGGCCGCGACGCACTGGTTCCTCTTCTCGAATCGCAAGGAGGATCC 1556
TrDcl1_SC58         CCCACCGCGATGGGGGGCCGCGACGCACTGGTTCCTCTTCTCGAATCGCAAGGAGGATCC 1558
                    ************************************************************
```

```
TrDcl1_Choachí      TGCGGTGGAGCATTCGCATATCCCACGGTATTATTTTACGGGCCGTGTGGCAACGCTGTG 1616
TrDcl1_SC58         TGCGGTGGAGCATTCGCATATCCCACGGTATTATTTTACGGGCCGTGTGGCAACGCTGTG 1618
                    ************************************************************

TrDcl1_Choachí      CCGCGGCGCCCGCGACAAGGTTGAGCAGGGAAACGAGACTGCCACGACGGCAGAAGAAGC 1676
TrDcl1_SC58         CCGCGGCGCCCGCGACAAGGTTGAGCAGGGAAACGAGACTGCCACGACGGCAGAAGAAGC 1678
                    ************************************************************

TrDcl1_Choachí      GGATCCCCCCGGCGTGGTTTTCAGCCGGTGCGAGTTTTTGAAGAACACCGAGCCCGCTGG 1736
TrDcl1_SC58         AGATCCCCCCGGCGTGGTCTTCAGCCGGTGCGAGTTTTTGAAGAACACTGAGCCCGCTGG 1738
                     ***************** ***************************** ***********

TrDcl1_Choachí      TTTCTCGGATGAGGAGGTGACTTGCGTCTTTGGCTCCGACCGGACAGCTGCGAACAAAGA 1796
TrDcl1_SC58         TTTCTCGGATGAGGAGGTCACTCGCGTCTTTGGCTCCGACCGGCCAGCTGCGAACAAAGA 1798
                    ****************** *** ******************** ****************

TrDcl1_Choachí      CGAAGGCGGTGAGGGGGCGGCGGCGCAGGAAGAGGAGCTTGCAATTTTCCGTGGTGCTAC 1856
TrDcl1_SC58         CGAAGGCGGTGAGGGGGCGGCGGCGCAGGAAGAGGAGCTTGCGATTTTCCGTGGTGCTAC 1858
                    ****************************************** *****************

TrDcl1_Choachí      CGCTACGATCTCGCTGGATATCGAAGGTGCGCAGGCGACGGTGCGTCACTTTGTGCAGAG 1916
TrDcl1_SC58         CGCTACGATCTCGCTGGATATCGTAGGTGGGCAGGCGACGGTGCGTCACTTTGTGCAGAG 1918
                    *********************** ***** ******************************

TrDcl1_Choachí      TCCAGAGCTGCAAGTTACGCGTGTCTCGTTTCCAAAGTATCCGCCCGGCGGAGGCTCTTT 1976
TrDcl1_SC58         TCCAGAGCTGCTAGTTACGCGTGTCTCGTTTCCAAAGTATCCGCCCGGCGGAGGCTCTTT 1978
                    *********** ************************************************

TrDcl1_Choachí      TTTTAACCGTCAAAAAACTATTGCCGAGTTTAAGAAGTGGGTGCTGGGCCAGGGGCTTGT 2036
TrDcl1_SC58         TTT-AACCGTCAAAAAACCATTGCCGAGTTTAAGAAGTGGGTGCTTGGCCAGGGGCTTGT 2037
                    *** ************** ************************** **************

TrDcl1_Choachí      GTACAGGAGCCTCCCTGCGGCGGATGACACCCGGGAGGAGCTG----------------- 2079
TrDcl1_SC58         GTCCAGGAGCCTCCCTGCGGCGGATGACACCCGGGAGGAGCTGTACGAGATCCGTGGGCG 2097
                    ** ****************************************

TrDcl1_Choachí      ----------------------------------------------------GAGGACAT 2087
TrDcl1_SC58         TGGTCTCTCGAACCAGGACTTTATCAACAGCGTTGCGAAGGAATTGGAGCTGGAGGACAT 2157
                                                                        ********

TrDcl1_Choachí      TCCCGACCGCAAGGCGCTTTCGCAGGCGTTGACCCGCGAGTGTGAAAGCAGGGAAATGAA 2147
TrDcl1_SC58         TCCCGACCCCAAGGCGCTTTCGCAGGCGTTGACCCGCGAGTGTGAAAGCAGGGAAATGAA 2217
                    ******** ***************************************************

TrDcl1_Choachí      CTACGAAATCTTGGATTTTGTTGGCGACGCTGTGATGGACTTTTTGGTGGCGTTTGACAG 2207
TrDcl1_SC58         CTACGAAATCTTGGAGTTTGTTGGCGACGCTGTGATGGACATTTTGGTGGCGTTTGACAG 2277
                    *************** ************************ *******************

TrDcl1_Choachí      TTTCCTGCTGGGGGAACCCTGGAATACACACGTCATCGCAGAGCTATGCTCCAATAAGCT 2267
TrDcl1_SC58         TTTCCTGCTGAGGGAACCCTGGAATACACACGTCATCGCAGAGCTATGCTCCAATAAGCC 2337
                    ********** ************************************************

TrDcl1_Choachí      GCTTGCCCATCTCATCCCTGTCTCGCTGAGTGAAAGACTCTCTCAGATTTATGTGGACCT 2327
TrDcl1_SC58         GCTTGCCCATCTCACCCCTGTCTCGCTGAGTGAAAGACTCTCTCAGATTTATGTGGACCT 2397
                    ************** *********************************************

TrDcl1_Choachí      GCCCACGAAGGTGAAGGCAGACATTATGGAGGCCATTCTGGGTGCTGTGTACTGCAGTCA 2387
TrDcl1_SC58         GCCCACGAAGGTGAAGGCAGACATTATGGAGGCCATTCTGGGTGCTATGTACTGCAGTCA 2457
                    *********************************************** ************

TrDcl1_Choachí      CATGGGGCTTGACAGGGTGCGTCAACAGCTACGGCACTTCTTCGGCCGCATCCCCGCCGC 2447
TrDcl1_SC58         CATGGGGCTTGACAGGGTGCGTCAACAGCTACGGCACTTCTTCGGCCGCATCCCCGCCGC 2517
                    ************************************************************

TrDcl1_Choachí      CATGGCGAGGGGGGGGGGGC-CATGCTCCATCCACACTGAAGTTGCTGGAG--------- 2497
TrDcl1_SC58         CATGGCGAGGGGGGGGGGGGGCATGATCCATCCACACTGAAGTTGCTGGAGCAGGCGGCA 2577
                    *******************  **** *************************

TrDcl1_Choachí      ------------------------------------------GAGCGATACAAGTACGCC 2515
TrDcl1_SC58         GCAGCCTGTCCATACCTTTGCAGCAACACAGAGCTGCTGGAGGAGCGATACAAGTACGCC 2637
                                                              ******************

TrDcl1_Choachí      TGCACCACATTAATTGATAAACGGAGCGCTGAGATATTCGTGACCCCGGCAACCTCTTCC 2575
TrDcl1_SC58         TGCACCACATTAATTGATAAACGGAGCGCTGAGATATTCGTGACCCCGGCAACCTCTTCT 2697
                    ***********************************************************

TrDcl1_Choachí      CTTGGAGGTCCCCACATGAGCCACTACGCCTCAGTACCGTTCAAGCGTATTCAGGACAAG 2635
TrDcl1_SC58         CTTGGAGGTCCCCACATGAGCCACTACGCCTCAGTACCGTTCAAGCGTATTCAGGACAAG 2757
                    ************************************************************

TrDcl1_Choachí      GCATACGCGACGCACTTCACGACAGGCAACGTGTACTCCTACCGGCGAATTTCTTCCATC 2695
TrDcl1_SC58         GCATACGCGACGCACTTCACGACAGGCAACGTGTACTCCTACCGGCGAATTTCTTCCATC 2817
                    ************************************************************

TrDcl1_Choachí      GATACCCCCGTGCTGTTCAACCGGATTCTGGACGCCTTTGCCAATGGTGCAACTGCCTTT 2755
TrDcl1_SC58         GACACCCCCGCGCTGTTCAACCGGATTCTGGACGCCTTTGCCAATGGTGCAACTGCCTTC 2877
                    ** ******* ************************************************

TrDcl1_Choachí      ACCAACGAAATCATTACAAGAGAAACGCACCTTGCGATTGATGTGGATGGACTAAGCGTC 2815
TrDcl1_SC58         ACCAACGAAATCATTACAAGAGAAACGCACCTTGCGATTGATATGGATGGACTAAGCGTC 2937
                    ******************************************* ****************

TrDcl1_Choachí      ACTTCCTGCGGCATTGCAAAGATGATTTGGGAGTGGTTTTGCAACACATTCGCATCCCGC 2875
TrDcl1_SC58         ACTTCCTGCGGCATTGCAAAGATGATTTGGGAGTGGTTTTGCAACACACTCGCATCCCGC 2997
                    ************************************************ ***********

TrDcl1_Choachí      TCGGCGATGCTTCTCCTGGACTGCTCGGGCATGTCCGTTATGTCGAATAAGATGAAGCGC 2935
TrDcl1_SC58         TCGGCGATGCTTCTCCCGGACTGCTCGGGCATGTCCGTTATGTCGAAAAAGATGAAGCGC 3057
                    **************** ****************************** ************

TrDcl1_Choachí      CCATGCCACATCCACTTTCCACAGGCGGCTACAAGTCTACAACGGATGCTGCCGCTTATA 2995
TrDcl1_SC58         TCATGCCACATCCACTTTCCACAGGCGGCTACAAGTCTACAACGGATGCTGCCGCTTATA 3117
                     ***********************************************************

TrDcl1_Choachí      ACGGATCTTCGACAGCACATTTTGGGGCGGGTGTCTCATACGACTCGGCTTGGTGATGTC 3055
TrDcl1_SC58         ACTGATCTTCGACAGCACATTTTGGGGCGGCTGTCTCATGCGACTCGGCTTGGTGATGTC 3177
                    ** *************************** ******** ********************
```

```
TrDcl1_Choachí      CTTGGTTTCCATGGGACTGAGGGAAGCGGGGAACCCGCAGCTGTCATTGGGAAAATTGTT 3115
TrDcl1_SC58         CTTGGTTTCCATGGGACGGAGGGAAGCGGGGAACCCGCAGCTGTCATTGGTAAAATTGTT 3237
                    ***************** ******************************** *********

TrDcl1_Choachí      TTTGCGACGGCGTACGCGATGCGTTGTGGCCCCCACAAAAAAGACATATGTGAAGATCCC 3175
TrDcl1_SC58         TTTGCGACGGCGTACGCGATGCGTTGTGGCCCCCACAAAAAAGACATATGTGAGGATCCC 3297
                    ***************************************************** ******

TrDcl1_Choachí      GTTTTGGACTGTCTGGATCTGCGCAGTATGCTGTGTCTTGGGGAGATCTGTGCTGCGATG 3235
TrDcl1_SC58         GTTTTGGACTGTCTGGATCTGCGCAGTATGCTGTGTCTTGGGGAGACCTGTGCTGCGATG 3357
                    *********************************************** ************

TrDcl1_Choachí      CGGCGGTATGTCTATTCCCACCTCACTAGCGTGGCGAGGCGGCCTTGCAAAGAGGCATAT 3295
TrDcl1_SC58         CGGCGGTATGTCTATTCCTACCTCACTAGCGTGGCGAGGCGGCCTTGCAAGGAGGCATAT 3417
                    ****************** ******************************* *********

TrDcl1_Choachí      TTTGTCAGGTTGGTCCCCAACTCTTGTTGTTTTGGATAACGCGGCCTCGCCAGCGACCAT 3355
TrDcl1_SC58         TTTGTCAGGTTGGTCCCCAACTCTTGTTGTTTTGGACAACGCGGCCTCGCCAGTGACCAT 3477
                    ************************************ **************** ******

TrDcl1_Choachí      GACGTTCTCATGAAGGTGCACTGGGCCTCAAAGGATGCAGCCGATGTAGTTGGTAAGTTT 3415
TrDcl1_SC58         GACGTTCTCATGAAAGTGCACTGGGCCTCAAAGGATGCAGCCGATGTAGTTGGTAAGTTT 3537
                    ************** *********************************************

TrDcl1_Choachí      GTTTGGTGTCCCGTGAGCAGGCTGGTGGAAGTCAGCGAGGAGTCGCTGGGCGTGGTGGAG 3475
TrDcl1_SC58         GTTTGGTGTCCCGTGAGCAGGCTGGTGGAAGTCAGCGAGGAGTCGCTGGGCGTGGTGAAG 3597
                    ********************************************************* **

TrDcl1_Choachí      TCTGTGGAGCGTGTAGTGGGTGAAGAGAGAATGAAGCCAAAGCAGTTCTTTATTGAGCGA 3535
TrDcl1_SC58         TCTGTGGAGCGTGTAGTGGGTGAAGAGAGAATGAAGCCAAAGCAGTTCTTTATTGAGCGA 3657
                    ************************************************************

TrDcl1_Choachí      GTCACGCCGTTGCGGGAGAAGAGTTTCTTTGCACCGGGGTTTTGGAATCGCGTCATTGAT 3595
TrDcl1_SC58         GTCACGCCGTTGCGGGAGAAGAGTTTCTTTGCACCGGGGTTTTGGAACCGCGTCATTGAT 3717
                    *********************************************** ************

TrDcl1_Choachí      ACCTCACTGGTGGAGAGCCGAAAGCTGCGGATGTACCTGAACGACAAGTACGACATGGTG 3655
TrDcl1_SC58         ACCTCACTGGTGGAGAGCCGAAAGCTGCGGATGTACCTGAACGACAAGTACGACATGGTG 3777
                    ************************************************************

TrDcl1_Choachí      TATGGTAGGGAGTACCGCCCACTCTTCCTGGACACCGTGCTTTGCTCTCAGGGCATAATA 3715
TrDcl1_SC58         TATGGTAGGGAGTACCGCCCACTCTTCCTGGACACCGTGCTTTGCTCTCAGGACATAATA 3837
                    **************************************************** *******

TrDcl1_Choachí      CGAGCCGTGCAGCCTTACGACAGCGCCTCTGGCGGCGTCATGCGAACCCCCACAAGTGCC 3775
TrDcl1_SC58         CGAGCCGTGCAGCTTTACGACAGCGCCTCTGGCAGCGTCATGCGAACCCCCACAAGTGCC 3897
                    ************* ******************* **************************

TrDcl1_Choachí      TCGAATGAAGAAGAGATGGACCAGTACCCTGTCGAGATTGCACACGCATCACTGCTGCGC 3835
TrDcl1_SC58         TCGAATGAAGAAGAGATGGACCAGTACCCTGTCGAGATTGCACACGCATCACTGCTGCGC 3957
                    ************************************************************

TrDcl1_Choachí      CTCACCTCCCTCCGCTGCCCTGAATACCGCGATGTGCAAGGGTTGAGCCGGCATGCCTGG 3895
TrDcl1_SC58         CTCACCTCCCTCCGCTGCCCTGAATACCGCGATGTGCAAGGGTTGAGCCGACATGCCTGG 4017
                    ************************************************** *********

TrDcl1_Choachí      TGGGACGGCTGCGACGACAACCCCAGTTTTGGAAACTTTGACGCCATTGAGGCCATCGAT 3955
TrDcl1_SC58         TGGGACGGCTGCGACGACGACGCCAGTTTTGGAAACTTTGACGCCATTGAGACCATCGAT 4077
                    ****************** ** ***************************** ********

TrDcl1_Choachí      GACCGACTGAAGTCCTTTGAGGATTATCAGGCGTGCGACAGCATCCCTGGCGCTGAGTGG 4015
TrDcl1_SC58         GACCGACTGAAGTCCTTTGAGGATTATCAGGCGTGCGACAGCATCCCTGGCGCTGAGTGG 4137
                    ************************************************************

TrDcl1_Choachí      GTCGTTTGGCTTGACGCCATCTTTGGAATAGTGGCCGGCGATTCCGAAAACTCGTTGGAG 4075
TrDcl1_SC58         GTCGTTTGGCTTGACGCCATCTTTGGAATAGTGGCCGGCGATTCCGAAAACTCGTTGGAG 4197
                    ************************************************************

TrDcl1_Choachí      AATGAAAAGGTGAAGACCGCGGCGCTGCAGCCCGCGTACTGGAAGTTTGTAAAGGAGACC 4135
TrDcl1_SC58         AATGAAAAGGTGAAGACCGCGGCGCTGCAGCCCGCGTACTGGAAGTTTGTAAAGGAGACC 4257
                    ************************************************************

TrDcl1_Choachí      CGTCAGGCACATCTTTTGGTGGCGGGGGAGGTGCTGCTTGCCGTTGGCTCCTGTGAGTCC 4195
TrDcl1_SC58         CGTCAGGCACATCTTTTGGTGGCGGGGGAGGTGCTGCTTGCCGTTGGCTCCTGTGAGTCC 4317
                    ************************************************************

TrDcl1_Choachí      CTGCAACAGGCGCTGGAGCAGAGGCGCGGCATGGAGACAATGGCGCGATGCCTAGTCCCC 4255
TrDcl1_SC58         CTGCAACAGGCGCTGGAGCAGAGGCGCGGCATGGAGACAATGGCGCGATGCCTAGTCCCC 4377
                    ************************************************************

TrDcl1_Choachí      AACATCACGATAAAAGATGTGCCGATTGACCCAAGGTGGCTGACGGCGTACGCAAAGGAC 4315
TrDcl1_SC58         AACATCACGATAAAAGATGCACCGATTGACCCAAGGTGGCTGACGGCGTACGCAAAGGAC 4437
                    ******************  ****************************************

TrDcl1_Choachí      GTCTTCCCGTTGTTTCTTCCCCTGCGCAAGAGGACCGTCGCCCCCGCTGCAGCCCCCAAA 4375
TrDcl1_SC58         GTCTTCCCGTTGTTTCTTCCCCTGCGCAAGAAGCCCGTCGCCCCCGCTGCAGCCCCCAAA 4497
                    ****************************** * *************************

TrDcl1_Choachí      GAACAGGCTGGGTTTGGGATGAACACTGCCTCCCTAATCGCCGTGACGGGCACGAGCATC 4435
TrDcl1_SC58         GAACAGGCTGGGTTTGGGATGAACACTGCCTCCCTAATCGCCGCGACGGGCACGAGCATC 4557
                    ******************************************* ****************

TrDcl1_Choachí      GACGGTGGCTTGACCGAGATGGAGCAGCCAGCAACAGCAGCACTCAACGGTGGTCTGCCA 4495
TrDcl1_SC58         GACGGTGGCTTGACCGAGATGGAGCAGCCAGCAGCAGCAGCACTCAACGGTGGTCTGCCA 4617
                    ********************************* **************************

TrDcl1_Choachí      AACAGGTTGAGGCAGACCCCTGGAAGGACGGTGCGGCTGCTTCTAGACAACTGGGATGGA 4555
TrDcl1_SC58         AACAGGTTGAGGCAGACTCCTGGAAGGACGGTGCGGCTGCTTCTAGACAACTGGGATGGA 4677
                    ***************** ******************************************

TrDcl1_Choachí      AGTGACCAGTCTATTGGCCAGGTCTATGCGGCGATTGTGTCGGCGTTTCGCGGATCGTAC 4615
TrDcl1_SC58         AGTGACCAGTCTATTGGCCAGGTCTATGCGGCGATTGTGTCGGCGTTTCGCGGATCGTAC 4737
                    ************************************************************

TrDcl1_Choachí      CGTCCCTCGCTATTTGTCACTTCCGAAGAAGGGTTGTTTTTGTTCCTTTCAAAGCAATTT 4675
TrDcl1_SC58         CGTCCCTCGCTATTTGTCACTTCCGAAGAAGGGTTGTTTTTGTTCCTTTCAAAGCAATTT 4797
                    ************************************************************
```

```
TrDcl1_Choachí       CCTTGCGTTGTGTCCCCCAATATTTTACAGCGCACGCCGTGAAAGGAGTCCATCGCGGG 4735
TrDcl1_SC58          CCTTGCGTTGTGTCCCCTCAATATTTTACAGCGCACGCCGTGAAAGGAGTTCATCGCGGG 4857
                     ***************** ******************************* **********

TrDcl1_Choachí       GCACAGCATGTTTTTGCCTACATCTTTGTTGTTGATGGTACGGCCAATCTGTACTGTAAT 4795
TrDcl1_SC58          GCACAGCATGTTTTTGCCTACATCTTTGTTGTTGATGGTACGGCCAATCCGCACCGTAAT 4917
                     ************************************************* * ** *****

TrDcl1_Choachí       GAGAGCCTCATCGCGGCAATATGGGAAGCTCTCGTAGTGAAAATCGGACGGATGTATGCC 4855
TrDcl1_SC58          GAGAGCCTCGTCGCGGCAATATGGGAAGCTCTCGTGGTGAAAACCGGACGGATGTATGCC 4977
                     ********* ************************** ******* ***************

TrDcl1_Choachí       TCCAGCGAGGCCATCTGAAAGTGTCTTCTTTTGTAGCCTCACCGCCGTCGACAACGATAA 4915
TrDcl1_SC58          TCCAGCGAGGCCATCCGAAAGTGTCTTCTTTTGTAGCCTCACCGCCGTCGACAACGATAA 5037
                     *************** ********************************************

TrDcl1_Choachí       CAAAAAGCGTA-- 4926
TrDcl1_SC58          CAAAAAGCGTACA 5050
                     ***********
```

```
TrRif4_Choachí      TGAGAAACTACTGGCTGTGGCGGCAATGCTGGAGGGCGGGAAGGAAAAGAACCATGAAGT 60
TrRif4_SC58         TGAGAAACTACTGGCTGTGGCGGCAATGCTGGAGGGCGGGAAGGAAAAGAACCATGAAGT 60
                    ************************************************************

TrRif4_Choachí      GGACGAAAAACATTAGACCGTTTGGGTTTTGTAATTTTTTTTTTTTTTTGCATGATCAA 120
TrRif4_SC58         GGACGAAAAACATTAGACCGTTTGGGTTTATG------TCTTTTTTTTTTGCATGATCAA 114
                    ***************************** **      * ********************

TrRif4_Choachí      CAGGCCTGTAGTGTCTACTTAGTGAGACTGGCGTC-TTTTTAAAACCTTTTCTGGTGGCG 179
TrRif4_SC58         CAGGTCTGTAGTGTCTACGTAGTGAGACTGACGTCCTTTTTAAAACCTTTTCTGGTAGCG 174
                    **** ************* *********** **** ******************** ***

TrRif4_Choachí      GTGAATGGTGGGAATGAGAGCGGGTTTTATTTGCATACTCTTTCAAACAAAATCAGGCGC 239
TrRif4_SC58         GGGAATGGTGGGAATGAGAGCGGGTTTTATTTGCATACTCTTTCAAACAAAATCAAGCTC 234
                    * ***************************************************** ** *

TrRif4_Choachí      AAAAACAAGAGCGTGTTTGCGTGTGTGTGTGTGTGTGTACTTATTGCAGCTCCTTTCT 299
TrRif4_SC58         AAAAACAAGAGCGTGTTTGCGTGTGTGTGTGTGTG--TACTTATTGCAGCTCCTTTCT 292
                    *************************************  *********************

TrRif4_Choachí      TGACGACCTTAGGAACAAGGAAAGCAGACAGTGATGTTGTACTCTGAAAGCGACCCCGAT 359
TrRif4_SC58         TGACGACCTCAGGAACAAGGAAGGCAGACAGTGATGTTGTACTCTGAAATCGACCCCGAT 352
                    ********* ************ ************************** **********

TrRif4_Choachí      ATTAGTGCGCAGAGTGTACTGGAGACCATCTCTGATGATAACAGCGCTGATATGCAGGGG 419
TrRif4_SC58         ATTAGTGCGCAGAGTGTACTGGAGACCATCTCTGATGATAACAGCGCTGATATGCAGGAG 412
                    ********************************************************** *

TrRif4_Choachí      CTTCTTATCGAAGCTGTCGCAGAGACGTACGAGCAGGAGCTGTACAATCATGCGAGAGCA 479
TrRif4_SC58         CTTCTCATCGAAGCTGTCGCAGAGACGTACGAGCAGGAGCTGTACAATCATGCGAGAGCA 472
                    ***** ******************************************************

TrRif4_Choachí      TTCTTTTACATCTTTGAGGACCCTGAGGGTCCTTTTCGACGGGCCTTTCCAGATCTCTAC 539
TrRif4_SC58         TTCTTTTACATCTTTGAGGACCCTGAGGGTCCTTTTCGACGGGCCTTTCCAGATCTCTAC 532
                    ************************************************************

TrRif4_Choachí      ACGCGGGATTCCTGTGAGGAGCCGGATTTAACTGATGTGCTCGAGGGGGCGTGTGATGAA 599
TrRif4_SC58         ACGCGGAATTCCTGTGAGGAGCCGGATTTAACTGATGTGCTCGAGGGCGCGTGTGATGAG 592
                    ****** **************************************** *********** 

TrRif4_Choachí      AGAAATACTGTGATAGTGGACGAGAGTTACGTTGGGCAACACGCCCCATCGCCGTTGTCA 659
TrRif4_SC58         GAAAATACTGTGATGGTGGACGAGAGTTACGTTGGGCAACACGCCCCATCGCCGTTGTCA 652
                      ************ *********************************************

TrRif4_Choachí      CCACCGCCACCAGCATTGATGTTCTCATGCAAAGAGGATATTGTCACGGAAGAGGGCGTG 719
TrRif4_SC58         CCACCGCCACCAGCATTGATGTTCTCATGCAAAGGGGATATTGTCACGGAAGAGGGTGTG 712
                    ********************************** ********************* ***

TrRif4_Choachí      ACAATAAATCCGTCACCCGCTACGAGATTTATTGTCACACCCCGGCAAGTGCCTGCTTAT 779
TrRif4_SC58         ACAATAAATCCGTCACCCGCTACGAGATTTATTGTCACACCCCGGCAAGTGCCTGCTTAT 772
                    ************************************************************

TrRif4_Choachí      TGCTATTGAACCCACTATGAATCGCGCGGAACTGCGTAAGAAACACGTCGAGGAGTATCC 839
TrRif4_SC58         TGCTATTGAACCCACTATGAATCGCGCGGAACTGCGTAAGAAACACGTCGAGGAGTATCC 832
                    ************************************************************

TrRif4_Choachí      TGGAATATATTTTTATGTGGGATATATTAAAGGTGAGCCTACGGGTTGGGCATTACTTCT 899
TrRif4_SC58         TGGAATAGATTTTTATTTGGGATATATTAAAGGTGAGCCTATGGGTTGGGCATTACTTCT 892
                    ******* ******** ************************ ******************

TrRif4_Choachí      CTCAGTGGATTCAGCCATTGTCGAGAAGTTTCAAAGTATGTATCCGGTTAACCGTTTTGC 959
TrRif4_SC58         CTCAGTGGATTCAGCCATTGTCGAGAAGTTTTAAAGTATGTATCCGGTTAACCGTTTTGC 952
                    ******************************* ****************************

TrRif4_Choachí      GACGGACGATGATCGGATGATGTTCTTCCGGGAGCGGCGTACGGGGAGAAGGGGTGGCTT 1019
TrRif4_SC58         GACGGACGATGATCGGATGATGTTCTTCCGGGAGCGGCGTACGGGGAGAAGGGGTGGCTT 1012
                    ************************************************************

TrRif4_Choachí      GACGCCGCCTGTCAAGGCTCCGAGTCAGCGCCCTAACCGTGCGCTTACCAAACGCATGCG 1079
TrRif4_SC58         GACGCCGCCTGTCAAGGCTCCGACTCAGCGCCCTAACCGTGCGCTTACCAAACGCATGCG 1072
                    *********************** ************************************

TrRif4_Choachí      GGATGTTGCGGAGCTTGCACGTCTTATTGGGACGGTTGGAGGCCCACTAAGGGCCATCCT 1139
TrRif4_SC58         GGATGTTGAGGAGCTTGCACGTCTTATTGAGACGGTTGGAGGCCCACTAAGGGCCATCTT 1132
                    ******** ******************** **************************** *

TrRif4_Choachí      TGTCGCCCTCGATGTGGAAGCCGCCGTTGTGCTTCCAGGTGGGGTTCTGCTTCCGTTGGA 1199
TrRif4_SC58         TGTCGCCCTCGATGTGGAAGCCGCCGTTGTGCTTCCAGGTGGAGTTCCGCTTCCGTCGGA 1192
                    ****************************************** **** ******** ***

TrRif4_Choachí      GCTGGCGCTGATTCCTCTTGGCACCGAACATGAGTTGCAAAGTTTTCACTGTTTTCTTCA 1259
TrRif4_SC58         GCTGGCGCTGATTCCTCTTGGCACCGAACATGAGTGGCAAAGTTTTCATTGTTTTCTTCA 1252
                    ********************************** ************* ***********

TrRif4_Choachí      CCCTGGTAGGGTTACAGATGAAGCTACTGCCTGTGCTTTGAGCTGTGGACACCTGAAGGG 1319
TrRif4_SC58         CCCTGGTAGGGTTACAGATGAAGCTACTGCCTGTGCTTTGAGCTGTGGACACCTGAAGGG 1312
                    ************************************************************

TrRif4_Choachí      AAGCCATTTTATACCCTTTCGTAATGCAAGCTTTCTCCGTCACGATTACACGGAAGTGGT 1379
TrRif4_SC58         AAGCCATTTTATACCCCTTCGTAATGCGAGCTTTCTCCGTCACGATTACACGGAAGTGGT 1372
                    **************** ********** ********************************

TrRif4_Choachí      GAGGACGCTTATTCCTTTTGTAACCAATGAGTGTGTCTTTTTTGTAAACAAGGGTTCCAT 1439
TrRif4_SC58         GAGGGCGCTTATTCCTTTTGTAGCCAATGAGCGTGTCTTTTTTGTAAACAAGGGTTCCAT 1432
                    **** ***************** ******** ****************************

TrRif4_Choachí      CATGGACGTTCACGCGCTGCGCTGGGTGTTTGGAGCAGCTTCTGCGTTGGAGGGGAGTGA 1499
TrRif4_SC58         CATGGACGTTCACGCGCTGCGCTGGGTGTTTGGAGCAGCTTCTGCGTTGGAGGGGAGTGA 1492
                    ************************************************************

TrRif4_Choachí      GTTTCCCATACCGTCTCTTCAAGAAATCTATTGCTTTGACATTGAAACCGTAACCAAAGT 1559
TrRif4_SC58         GTTTCCCATACCGTCTCTTCAAGAAATCTATTGCTTTGACATTGAAACCGTAACCAAAGT 1552
                    ************************************************************

TrRif4_Choachí      TTTCTCTCGGGGTGATGAAGACGGTGAGACGAATGCTGGGGATGGTACGGCGTGTTGGTA 1619
TrRif4_SC58         TTTCTCTCGGGGTGATGAAGACGGTGAGACGAATCCTGGGGATGGTACGGCGTGTTGGTA 1612
                    ********************************** *************************
```

```
TrRif4_Choachí       CCATAGCAAGATGGAACGGATGATTTCTCAAGATGTAATTCCAGATTCCCACCCACACTG 1679
TrRif4_SC58          CCATAGCAAGATGGAACGGATGATTTCTCAAGATGTAATTTCAGATTCCCACCCACACTG 1672
                     **************************************** *******************

TrRif4_Choachí       CGCGTTACGGGATGCCGGGCGTCTCTATAACGAATTACGTCTTTTGGTTGGGAATTTCCG 1739
TrRif4_SC58          CGCGTTACGGGATGCCGGGCGTCTCTATAACGAATTACGTCTTCTGGTTGGGAATTTCCG 1732
                     ******************************************* ****************

TrRif4_Choachí       TTCCATGGAAGCTAACGGACACTGAAGTCATGGAGAAGAACCGAAGAGTGGGA----- 1792
TrRif4_SC58          TTCCATGGAAGCTAACGGACACTGAAGACATGGAGAAGAACCGAAGAGTGGGAATCGA 1790
                     *************************** *************************
```

```
TrRif5_Choachí      TGGACACGCGTGCAGAAAGGCCTGGACGCAAACACAATTGAACGAGACAATGTTATTGTT 60
TrRif5_SC58         TGGACACGCGTGCAGAAAGGCCTGGACGCAAACACAATTGAACGAGACAATGTTATTCTT 60
                    ********************************************************* **

TrRif5_Choachí      TCCATCAATGACATTGCCTGGATTGGGAAAAATGTATTTGTTGTATTTGTGCCAGCGTAC 120
TrRif5_SC58         TCCATCAATGCCATTGCCTGGATTGGGAAAAATGTATTTGTTGTATTTGTGCCAGCGTAC 120
                    ********** *************************************************

TrRif5_Choachí      AAGCGAAGGATTAGGTGCGAGCTGACGGCCGTGGATGTGCCACGCGGCACGGCCTCGGCC 180
TrRif5_SC58         AGGCGAAGGATTAGCTGCGAGCTGACGGCCGTGGATGTGCCACGCGGCACGGCCTCGGCC 180
                    * ************ *********************************************

TrRif5_Choachí      CTTGAGGAAAAAGTAATGAGAACACTGAATGAAATTCGCTGTGCGCCGCACAAGTATCGT 240
TrRif5_SC58         CTTGAGGAAAAAGTAATGAGAACACTGAATGAAATTCGCTGTGCGCCGCACAGGTATCTT 240
                    **************************************************** ***** *

TrRif5_Choachí      CGACAGGACCACTATGCCTTCTTTCCAAATCAAGGCTACGGAGATATTATATTTAGTATG 300
TrRif5_SC58         CGACAGGACCACTATGCCTCCTTTCCAAATCAAGGCTACGGAGATATTATATTTAGTATG 300
                    ******************* ****************************************

TrRif5_Choachí      AGTGACAACGCTACACAGTAGTTTTCGTTGCAGGAGTCTTTGCTGCTGCGTGGGGTTGGC 360
TrRif5_SC58         AGTGACAACGCTACACAGTGGTTTTCGTTGCAGGAGTCTTTGCTGCTGCGTGGGGTTGGC 360
                    ******************* ****************************************

TrRif5_Choachí      AGAATTGTGCGTCCCATAGTTACACGACTGGAGGAACGTATACACGCGGAGTTTGAAGGC 420
TrRif5_SC58         AGAATTGTGCGTCCCATAGTTACACGACTGGAGGAACTTATACACGCGGAGTTTGAAGGC 420
                    ************************************* **********************

TrRif5_Choachí      TCAAGGATCCTGGCGCATCCCCCAGACGATCGTGAGCACAGCCGAGTGTGTGGGCAAACC 480
TrRif5_SC58         TCAAGGATCCTGGCGCATCCCCCAGACGATCGTGAGCACAGCCGTGTGTGTGGGCAAACC 480
                    ******************************************** ***************

TrRif5_Choachí      ATCATTTAAAATTTGAAAGCGGACCACCGTTTCGCAAGCAATTGATAGGCCAACTAATAG 540
TrRif5_SC58         ATCATTTAAAATTTGAAAGCGGACCACTGTTTCGCAAGCAATTGACAGGCCAAATAATAG 540
                    *************************** ***************** ******* ******

TrRif5_Choachí      ATGTTTTGCGGGGTGATGTGTATGTGGTTCGCTGGGACGAGGGACAAGTTATGAGCGGCA 600
TrRif5_SC58         ATGTTTTGCGGGGTGATGTGTATGTGGTTCGCTGGGACGAGGGACAAGTTATGAGCGGCA 600
                    ************************************************************

TrRif5_Choachí      A-TGTACGAGTAAACAAAGTGGTAGCAAGGATACTGGTGACGCACACCACGAAGAGTGGC 659
TrRif5_SC58         AATGTACGAGTAAACAAAGTGGTAGCAAGGATACTGGTGACGCACACCACGAAGAGTGGC 660
                    * **********************************************************

TrRif5_Choachí      CACCGCGGCTTGTGGTTCTTGACGCTGTCAAGTGTTTTTCGTCGCGTACCAAGAATGGCT 719
TrRif5_SC58         CACCGCGGCTTGTGGTTCTTGACGCTGTCAAGTGTTTTTCGTCGCGTACCAAGAATGGCT 720
                    ************************************************************

TrRif5_Choachí      TTGCGGCGCTGCGGTGGGCACAGAAGCGTCTTCTCTACTCCCGCGTTGAAGTCTGCATTC 779
TrRif5_SC58         TTGCGGCGCTGCGGTGGGCACAGAAGCGTCCTCTCTACTCCCGCGTTGAAGTCTGCATTC 780
                    ****************************** *****************************

TrRif5_Choachí      ACTCCACACACCCGTCGGAGAAGGACGTATCAAATAACACACAACTCTTCTCTTATGAGC 839
TrRif5_SC58         ACTCCACACACCCGTCGCAGAAGGACGTGTCAAATAACACACAACTCTTCTCTTATGAGC 840
                    ***************** ********** *******************************

TrRif5_Choachí      AATTGCGATGTGTGGGGTCAACTGTGTACGTGCTACCACGGGAATCTGGCCCGTGTGCGT 899
TrRif5_SC58         AATTGCGATGTGTGCGGTCAACTGTGTACGTGCTACCACGGGAATCTGGCCCGTGTGCGT 900
                    ************** *********************************************

TrRif5_Choachí      CTTCCACGAGGCAAGTCACCAATGTAGGTGTTGCCTTAATTTCAAAGGGTCTCGGACTTC 959
TrRif5_SC58         CTTCCACGAGGCAAGTCACCAATGTAGGTGTTGCCTTAATTTCAAAGGGTCTCGGACTTC 960
                    ************************************************************

TrRif5_Choachí      TTCGACAGGTTGTGGATACTGCGGTACCGCGTTCAAGTGATTTTCATGAACTTCTACGTG 1019
TrRif5_SC58         TTCGACAGGTTGTGGATGCTGCGGTACCGCGTTCAAGTGATTTTCATGAACTTCTACGTG 1020
                    ***************** ******************************************

TrRif5_Choachí      CTGCCAGGGACCGGTGTTGCGCAGAGGCAGACGCGACACTGAAGGATGCAAAACAGTCTT 1079
TrRif5_SC58         CTGCCAGGGACCGGTGTTGCGCAGAGGCAGACGCGACACTGAAGGATGCAAAACAGTCTT 1080
                    ************************************************************

TrRif5_Choachí      TATCATACGATTTGCCGATCTTCGATGATGGAGTCTCGTGCCCTGCAAATGTTTCCCTTG 1139
TrRif5_SC58         TACCATACGATT-GCCGATCTTCGACGATGGAGTCTCGTGCCCTGCAAATGTTTCCCTTG 1139
                    ** ********* ************ **********************************

TrRif5_Choachí      ATACGTGGTGCATCTCTTTCCCATCGCTGAAGACGCTGTGGCAGACCACAATGTGAGATG 1199
TrRif5_SC58         ATACGTGGTGCATCTCTTTCCCATCGCTGAAGACGCTGTGGCAAACCACAATGTGAGATG 1199
                    ******************************************* ****************

TrRif5_Choachí      GAGATGTTCATCGCAGTGCGCACGATTACCCCCAACGCATTCACAGTCTTCCACCACTGC 1259
TrRif5_SC58         GAGATGTTCATCGCAGTGCGCACGATTACCCCCAACGCATTCACAGTCTTCGGCCACTGC 1259
                    ***************************************************  *******

TrRif5_Choachí      GAAGCCTCTGTGATATGAGCACACTGATACGTGAATGTGAGACCTTTGAGGGCTTTATCA 1319
TrRif5_SC58         GACGCCTCTGTGATATGAGCACACTGATACGTGAAAGTGAGACCTTTGAGGGCTTTATCA 1319
                    ** ******************************* *************************

TrRif5_Choachí      ATTCCGTGGCGTTAGTGCGGAAGTCTCTTGGTGCGTCTTTAGAGCAGCAGCAATGTCGGC 1379
TrRif5_SC58         ATTCCGTGGCGTTAGTGCGGGAGTCTCTTGGTGCGTCTTTGGAGCAGCAGCAATGTCGGC 1379
                    ******************** ******************* *******************

TrRif5_Choachí      GTTGTCAGTTTCTTCGTGTGGAAATTAACGCTATTTTTGCTCTTGCGAGGATTCAAGATA 1439
TrRif5_SC58         GTCGTCAGTTTCTTCGTGTGGAAATTAACGCTATTTTTGCTCTTGCGAGGATTCAAGATA 1439
                    ** *********************************************************

TrRif5_Choachí      TCCTTGCCAGAGTTGAGGCAAACACCCTTGGATCTGTTTGTCTTTATCCCACTGAAAAGA 1499
TrRif5_SC58         TCCTTGCCAGAGTTGAGGCAAACACCCTTGGATCGGTTTGTCTTTATCCCACTGAAAAGA 1499
                    ********************************** *************************

TrRif5_Choachí      GTATCTTGGGCAACACTGTCGAAGTGACAGTGGTATACGACAGGTGGCCTAGCGTCATTC 1559
TrRif5_SC58         GTATCTTGGGCAACACTGTCGAGGTGACAGTGGTATACGACAGGTGGCCTGGCGTCATTC 1559
                    ********************** *************************** *********

TrRif5_Choachí      CTATTACAGAAACCGTTTTACTTTACGTTGCCGTCACCGGGTATGTGCCGCAGTCGTACG 1619
TrRif5_SC58         CTATTACAGAAACCGTTTTACTTTACGTTGCCGTCACCGGGTATGTGTCGCAGTCGTACG 1619
                    *********************************************** ************
```

```
TrRif5_Choachí      ACCCTACGAAACACATCCCGACGGATTCTCAGCACGAGCTGACCGTGAATAAGGAGCAAA 1679
TrRif5_SC58         ACCCTACGAAACACATCCCGACGGATTCTCAGCACGAGCTGACCGTGAATAAGGAACAAA 1679
                    ******************************************************* ****

TrRif5_Choachí      GGCATGGGACGAAGGTGATTAAGCTGAAACTTTCAGATGATGAGAATGAAATAATTTTTC 1739
TrRif5_SC58         GGCATGGGACGAAGGTGATTAAGCTGAAACGTTCAGATGATGAGAATGAAATAATTTTTG 1739
                    ****************************** ***************************** 

TrRif5_Choachí      TTGTTGATGTTAGGGTGGAATTTGTAAACCACACTGAGCAGCTGAATCATCGGTGTGTGT 1799
TrRif5_SC58         TTGTTGATGTTAGGGTGGAATTTGTAAACCGCACTGAGCAGCTGAATCATCGGTGTGTGT 1799
                    ****************************** *****************************

TrRif5_Choachí      ATTTTCTCACCAGAGCGGCACGGGGCTCTGGTACCTCAGGCACGTTGCTGTATGATGATG 1859
TrRif5_SC58         ATTTTCTCACCAGAGCGGCACGGGGCCCTGGTACCTCAGGCACGTTGCTGTATGATGATG 1859
                    ************************** *********************************

TrRif5_Choachí      ATGACCTTGTTAGTGTGGACGGTGGGGAAATCCACTCCGCGTGTGGTAGTGACAGTGAAG 1919
TrRif5_SC58         ATGACCTTGTTAGTGTGGACGGTGGGGGAACCCACTCCGCGTGTGGTGGTGACAGTGAAG 1919
                    *************************** ** **************** ************

TrRif5_Choachí      AGGAGTCTGACAGACAAAACGAGCAAAATCAAGGCGCGGAAGAGGCAGGGCCAGCAGGCA 1979
TrRif5_SC58         AGGAGTCTGACAGACAAAACGAGCAAAATCAAGGTGCGGAAGAGGCAGGGCCAGCAGGCA 1979
                    ********************************** *************************

TrRif5_Choachí      ATGATAATGAAACGACCAATATAAAAGAGGAAATGGAGTCGTCGGAGATGTTTTGTCGTT 2039
TrRif5_SC58         ATGATAATGAAACGGCCAAGATAAAAGAGGAAATGGAGTCGTCGGAGATGTTTTGTCATT 2039
                    ************** **** ************************************* **

TrRif5_Choachí      CGAGTGCTGATTTTTACTTTCAAGTGATGACTGACCAGGCGCATAAAGGGGGAGAATTCA 2099
TrRif5_SC58         CGAGTGTTGATTTTTACTTTCAAGTGATGACTGATCAGGCGCATAAAGGGGGAGAATTCA 2099
                    ****** *************************** *************************

TrRif5_Choachí      TGCGGAGCCTGCGCGACTGGAGCAGACGTCTCATGTACCGTGATGTGCGATGTGTGCCAA 2159
TrRif5_SC58         TGCGGAGCCTGCGCGATTGGAGCAGACGTCTCATGTACCGTGATGTGTGGTGTGTGCCAA 2159
                    **************** ****************************** * **********

TrRif5_Choachí      TGGGCATTGGCACTGTGAATGGAGCTCGTGGGTTTTTGGGAGATGTGCTGCTGATAAAGA 2219
TrRif5_SC58         TGGACATTGGCACTGTGAATGGAGCTCGTGGGTTTTTGGGAGATGTGCTGCTGATAAAGA 2219
                    *** ********************************************************

TrRif5_Choachí      CGCAGGAGGAGGAAGAAGAGGAGGGACACGTTGCTGCGGAGGACCAAAGTCTTTTTTTTT 2279
TrRif5_SC58         CGCAGGAGGAGGAAGAAGAGGAGGGACACGTTGCTGCGGAGGACCAAAGTCTTTTTTTTT 2279
                    ************************************************************

TrRif5_Choachí      TT---ACGGTGGAAAAGAAGTAGCGTCCGCCGGCTCATTCATCACGGTTAACAGGGGGGT 2336
TrRif5_SC58         TTTTTACGGTGGAAAAGAAGTAGCGTCTGCCGGCTCATTCATCACGGTTAACAGGGGGGT 2339
                    **   ********************** ********************************

TrRif5_Choachí      GATGCGTGGACGGACACGACTCAGCTATTTGAGCAACACCATCGCAGACGTTGCAATGTT 2396
TrRif5_SC58         GATGCGTGGACGGACACGACTCAGCTATTTGAGCAACACCATCGCAGACGTTGCAATGTT 2399
                    ************************************************************

TrRif5_Choachí      TTGTTTAAGCGGGAGTTTGAGTCACGGGACCTTCTGATAAAAGCTAGGAACGTGTTGATT 2456
TrRif5_SC58         TT----AAGCGGGAGTTTGAGTCACGGGACCTTCTGATTAAAGCTAGGAACGTGTTGATT 2455
                    **    ******************************** *********************

TrRif5_Choachí      TTCCGTATGCGTGGAAGGTTATCTAAAACGGTGGATATTTCAGTTATGGAAGGGAACATG 2516
TrRif5_SC58         TGCCGTATGCGTGGAAGGTTATCTAAAACGGTGGATATTTCAGTTATGGAAGGGAACATG 2515
                    * **********************************************************

TrRif5_Choachí      GTTTTGGATTCTGCGATATGTTTGGAGGAGTATGGTGCTTTTTTGGCTTGCGGCAAGCCA 2576
TrRif5_SC58         GTTTTGGATTCTGCGATATGTTTGGAGGAGTATGGTGCTTTTTTGGCTTGCGGCAAACCA 2575
                    ******************************************************** ***

TrRif5_Choachí      GTTGATGATGGCGGTGCCGTAATTTATGCCGAGGCGGGGAGAATGGAATGCTGGGACGAC 2636
TrRif5_SC58         GTTGATGATGGCGGTGCCGTAATTTATGCCGAGGCGGGGAGAATGGAATGCTGGTACGAC 2635
                    ****************************************************** *****

TrRif5_Choachí      TCTGACACTACTGTTTCCGCTGCGGGGCCTACGACGACTGCCGTAGCCGCCAACTTGCAG 2696
TrRif5_SC58         TCTGACACTACTGTTTCCGCTGCGGGGCCTACGACGACTGCCGTAGCCGCCAACTTGCAG 2695
                    ************************************************************

TrRif5_Choachí      ACTGTTGCGTTTCAAGGCCACGGCGCATCCTTTGGCTTCATCTCACCATCATTTTTTGCT 2756
TrRif5_SC58         ACTGTAGCGTTTCAAGGCCACGGCGCATCCTTTGGCTTCATCTCACCATCATTTTT-GCC 2754
                    ***** ************************************************** ** 

TrRif5_Choachí      GCCCAGGGCTCCTTTGTCTGCGTTGTCTATTTTTCCGCGTCAGCTTTTTTGCCGCGTACC 2816
TrRif5_SC58         GTCCAGGGCTCCTTTGTCTGCGTTGTCTATTTTTCCGCATCAGCCTTTTTGCCGCGTACC 2814
                    * ************************************ ***** ***************

TrRif5_Choachí      ACAAACAACATCGTGCGTTGGTGGCTAAGAACAACTTGGCCTTCATGAAGGAGGGTGGGG 2876
TrRif5_SC58         ACAAACAACATCGTGCGTTGGTGGCTAAGAACAACTTGGCCTTCATGAAGGAGGGTGGGA 2874
                    *********************************************************** 

TrRif5_Choachí      ATACTCACGTGGATGCGGCCTTTATTCGACTGCCCCATCGTTTTTTCCCCAATCTTTTTG 2936
TrRif5_SC58         ATATTCACGTGGATGCGGCCTTTATTCGACTGCCCCATCGTTTTTTGCCCAATCTTTTTG 2934
                    *** ****************************************** *************

TrRif5_Choachí      CCTCAAACTTTGAGCGCCTGCCAGCGGCTCTGCAGAATGTGGAGCGGGCCTTTTGTGAAT 2996
TrRif5_SC58         CCTCAAACTTTGAGCGCCTGCCAGCGGCTCTGCGGAATGTGGAGCGGGCCTTTTGTGAAT 2994
                    ********************************* **************************

TrRif5_Choachí      CGTTGCAACAAGGTGATGTGCCGCGTCCGCCCAACGCGCCGGAGGTCGTATATTACGCAA 3056
TrRif5_SC58         CGTTGCAACAAGGTGATGTGCCGCGTCCGCCCAACGCGCCGGAGGTCGTATATTACGCAA 3054
                    ************************************************************

TrRif5_Choachí      CACAAAAAGCTTTTGTTCCTATTTTATCTATGCTTTTGCTGCTTCAGCTGCGACGTGATA 3116
TrRif5_SC58         CACAAAAAGCTTTTGTTTCTATTTTATCTATGCTTTTGCTGCTTCAGCTGCGACGTGATA 3114
                    ***************** ******************************************

TrRif5_Choachí      ATTCGGGAAAGGAATCCAGTGCAGAAACCCACCCGCACACGTCGGTATACAAGTTACTGA 3176
TrRif5_SC58         ATTCGGGAAAGGAATCCAGTGCAGAAACCCACCCGCACACGTCGGTATACAAGTTGCTGA 3174
                    ******************************************************* ****

TrRif5_Choachí      GGGATAAGTATGGTGCCCTGTACGATGTGAAGATGTCCAGGTTTACTACTTCTCTGGTGC 3236
TrRif5_SC58         GGGATAAGTATGGTGCCCTGTACGATGTGAAGATGTCCAGGTTTACTACTTCTCTGGTGC 3234
                    ************************************************************
```

```
TrRif5_Choachí      TTTACAGGGTGGAGCCCAACTTAAACGCGAATGGGGATGGACAACAACGGCAGCCTGCAT 3296
TrRif5_SC58         TTTACAGGGTGGAGCCCAACCTAAACGCGAATGGGGGTGGACGACAACGGCAGCCTGCAT 3294
                    ******************** *************** ***** *****************

TrRif5_Choachí      TATTTCTTGGATCTCATGACACAAAAACGAGTGACGCATTTGAAGCCCCTGGCGACTTTA 3356
TrRif5_SC58         TATTTCTTGGATCTCATGACACAAAAACGAGTGACACATTTGAAGCCCCTGGCGACTTTA 3354
                    *********************************** ************************

TrRif5_Choachí      CGACATTGGGTAGAAAGTCCTTGTCTCGTGCGATTGAAGTGCTGGGGTTTGAATCACTTG 3416
TrRif5_SC58         CGACATTGGGTAGAAAGTCCTTGTCTCGTGCGATTGGAGTGCTGGGGTTTGAATCACTTG 3414
                    ************************************ ***********************

TrRif5_Choachí      TTGCGCCTACTCCGGCCTCGCCAGCCGACGATTTGACGAACAACGACGATGACACTGTTT 3476
TrRif5_SC58         TTGCGCCTACTCCGGCCTCGCCAGCCGACGATTTGACGAACAACGACGATGACACTGTTT 3474
                    ************************************************************

TrRif5_Choachí      TACTAACGGAGTTGAGTGATGAGGAATGTCATCCTATGACTTCCCGTGGTGTCGGGCCTT 3536
TrRif5_SC58         TACTTACGGAGTTGAGTGATGAGGAATGTCATCCCATGACTTCCCGTGGTGTCGGGCCTT 3534
                    **** ***************************** *************************

TrRif5_Choachí      GTCCGTTAGCAAGCAGCACCCCAAGGCTTTACCATCCATTTCCGTGGCTCACGCTTCTCG 3596
TrRif5_SC58         GTCCGTTAGCAAGCAGCACCCCAAGGCTTTACCATCCATTTCCGTGGCTCACGCTTCTCG 3594
                    ************************************************************

TrRif5_Choachí      GCCAACCGCTGCTTGCTCGAGATGAATACGACGCAGAATCGTTGGGCAATCACGAAATGG 3656
TrRif5_SC58         GCCAACCGCTGCTTGCTCGAGACGAATACGACGCAGAATCGTTGGGCAATCACGAAATGG 3654
                    ********************** *************************************

TrRif5_Choachí      TTGCTGAGGGAGGCCAACTCGTTCGACGGCACATTGCGGTTTCATACTCAACTGTTTGCT 3716
TrRif5_SC58         TTGCTGAGGGAGGCCAACTCGTTCGACGGCACATTGCGGTTTCATACTCAACTGTTTGCT 3714
                    ************************************************************

TrRif5_Choachí      TGAGTGAAAGAAAATCATATTATTCATTTTCCTATGGTCGAGTAGCGCTGTCGTTGATTT 3776
TrRif5_SC58         TGAGTGAAAGAAAATCATATTATTCATTTTCCTATGGTCGAGTAGCGATGTCGTTGATTT 3774
                    *********************************************** ************

TrRif5_Choachí      ATGGAATTGTGAAAAGACAAGCAGGGAAGGGCTCCGCTATCATGAACTATTTATCTCTTC 3836
TrRif5_SC58         ATGGAATTGTGAAAAGACAAGCAGGGAAGGGCTCCGCTATCATGAACTATTTATCTCGTC 3834
                    ********************************************************* **

TrRif5_Choachí      TGCTTCGTTCTGCAGGCACTAGAGCCGAGGGTGCCTCTCCGTTTGCAGATGGAATCTTGA 3896
TrRif5_SC58         TGCTTCGTTCTGCAGGCACTAGAGCCGAGGGTGCCTCTCCGTTTGCAGATGGAATCTTGA 3894
                    ************************************************************

TrRif5_Choachí      ATGTGTACGGCACGGCGAGTGAACCGTTGTACC---TATTCTATCAGTCTCGTCGACAAC 3953
TrRif5_SC58         ATGTGTACGGCACGGCGAGTGAACCGTTGTACCACCTATTCTATCAGTCTCGTCGACAAC 3954
                    *********************************   ************************

TrRif5_Choachí      TTCGCTTTCGACTTGGCATGCTTTTGCCTTCTCTTTTGAAGGGAGGGGAAGACACGACGT 4013
TrRif5_SC58         TTCGCTTTCGACTTGGCATGCTTTTGCCTTCTCTTTTGAAGGGAGGGGAAGACACGACGT 4014
                    ************************************************************

TrRif5_Choachí      CTCCGACGGGCGCTGACGTGAAAGCCGAAGAGAAATTCCTACAACAGTTGTCCGAGGCAG 4073
TrRif5_SC58         CTCCGACGGGCGCTGACGTGAAAGCCGAAGAGAAATTCCCACAACAGTTGTCCGAGGCAG 4074
                    *************************************** ********************

TrRif5_Choachí      GGAAGCAGCTTCTTGACTTGGTGGCGAAGCAGACAGCTGAACCATCGAGTTACGAGAGTG 4133
TrRif5_SC58         GGAAGCAGCTTATTGACTTGGTGGCGAAGCAGACAGCTGAACCATCGAGTTACGAGAGTG 4134
                    *********** ************************************************

TrRif5_Choachí      ACGATGGGAAACCTCCCGCACGCGTGTACCTTAAAATAAATCTTGAACTTGATTCCTTCG 4193
TrRif5_SC58         GCGATGGGAAACCTCCCGCACGCGTGTACCTTAAAATAAATCTTGAACTTGATTCCTTCG 4194
                     ***********************************************************

TrRif5_Choachí      ACGGCGCAGGCAGGTGTATGTGGGTCTCTGTGTACCCCATCAAGAATGAACCAGAAACCA 4253
TrRif5_SC58         ACGGCGCAGGCAGGTGTATGTGGGTCTCTGTGTACCCCATCAAGAATGAACCAGAAACCA 4254
                    ************************************************************

TrRif5_Choachí      AGGGATATGTTGAGGCAGTTGGAACGCGCGCCAATCCTCATCAGCAGTTTGACCTTGCGT 4313
TrRif5_SC58         AGGGATATGGCGAGGCAGTTGGAACGCGCGCCAATCCTCATCAGCGGTTTGACCTTGCGT 4314
                    *********  ********************************** **************

TrRif5_Choachí      CCAATGTGTGGTCTCGATTCCCGCAGCACACACTTCTCGTGAGGTATCGCCCCGAGCACG 4373
TrRif5_SC58         GCAGTGTGTGGTCTCGATTCCCGCAGCACACACTTCTCGTGAGGTATCGCCCCGAGCACG 4374
                     ** ********************************************************

TrRif5_Choachí      ACGAGTATGTTTTTGCGTGGTCGGAAGCTGAAAAACTGGCCCCAGTGGACGCTGAGGTGG 4433
TrRif5_SC58         ACGAGTATGTTTTTGCGTGGTCGGAAGCTGAGAAACTGGCCCCAGTGGACGCTGAGGTGG 4434
                    ******************************* ****************************

TrRif5_Choachí      CCA 4436
TrRif5_SC58         CCA 4437
                    ***
```
