# Supplementary material for: Genome of the Avirulent Human-Infective Trypanosome—Trypanosoma rangeli
Source: PLoS Negl Trop Dis. 2014 Sep 18;8(9):e3176. doi: 10.1371/journal.pntd.0003176 (PMC4169256; doi:10.1371/journal.pntd.0003176)
Supplement: Figure S5 — Conservation of DCL-2 in RNAi-positive trypanosomes and T. rangeli . Panel A shows a multiple alignment of potential DCL2 proteins from T. b. gambiense, T. b. brucei, T. congolense and T. rangeli generated by MultiAlin. Amino acids in red are conserved in all sequences. Panel B summarizes the identity shared by the potential DCL2 proteins. The lysine and glutamic acid residues highlighted in green are part of the RNaseIII domain of DICERs, which have been shown to be important for the catalytic activity of TbDCL2 [110]. (PDF) [file pntd.0003176.s005.pdf]

A)

```
                   1                                                                                        90
TrDCL2_Choachí                                                           MNVGKMARIHSELWSHMPLAKESRARGYATPDTDVF
TrDCL2_Sc58                                                              MNVGKMARIHSELWSHMPLAKESRARGYATPDTDVF
TcIL3000.3.480     MLSSEEGNAHLSESNRQHTSANLSKTPQESTVRCRRGRWVIKLALALFLSGKELHTEGKLAAFHGEIWSYELLGREALRRQYVPPDTRPK
TcIL3000.0.20050   MLSSEEGNAHLSESNRQHTSANLSKTPQESTVRCRRGRWVIKLALALFLSGKELHTEGKLAAFHGEIWSYELLGREALRRQYVPPDTRPK
Tbg972.3.980       MIFGEDIEPQDSVAVSCGSTNMVNNTPKEALVLQRRGRWVMKLAVALFLSGKDLQSEGELSIMHSEIWSYAALGNEAVRRGYVPLGTPVA
Tb427.03.1230      MXFGEDIEPQDSVAVSCGSTNMVNNTPKEALVLQRRGRWVMKLAVALFLSGKDLQSEGELSIMHSEIWSYAALGNEAVRRGYVPLGTPVA
Tb927.3.1230       MIFGEDIGPQDSVAVSCGSTNMVNDTPKEALVLQRRGRWVMKLAVALFLSGKDLQSEGELSIMHSEIWSYAALGNEAVRRGYVPLGTPVA
 Consensus         m...e......s............tp.e..v..rrgrwv.kla.alflsgk.l..eGk$a..HsEiWSy..Lg.Ea.rRgYvppdT.v.

                   91                                                                                      180
TrDCL2_Choachí     VTGSKWKQRLGIMWLKEGFGACVAELEPYFQREFLKLQEQTEPQEQVDITTAWTSDDPSAECRADIEACTTVASPLVSPLALVGFERQLA
TrDCL2_Sc58        VTGSKWKQRLGIMWLKEGFGACVAELEPYFQREFLKLQEQTEPQEQVDITTAWTSDDPSAECRADIEACTTVASPLVSPLALVGFERQLA
TcIL3000.3.480     AAQNVWKEKIGAMYVDGGFEACVRDLNPYFEREFNRIQKKAEEERQITADT----ANTVACHAQVADATHSVGGFSSGSTVDSALVKYFA
TcIL3000.0.20050   AAQNVWKEKIGAMYVDSGFEACVRDLNPYFEREFNRIQKKAGEERQITADT----ANTVACHAQVADATHSVGGFSSGSTVDSALVKYFA
Tbg972.3.980       GAQSVWKRKLGELWLEKGFEPCMIQLEPYFQQEFERLLAKGILKPQSGTEK----DSNCSAVRPV-DTPEPARLSSRNNGAVEEFAKPLA
Tb427.03.1230      GAQSVWKRKLGELWLEKGFEPCMIQLEPYFQQEFERLLAKGILKPQSGTEK----DSNCSAVRPV-DTPEPARLSSRNNGAVEEFAKPLA
Tb927.3.1230       GAQSVWKRKLGELWLEKGFEPCMIQLEPYFQQEFERLLAKGILKPQSGTEK----DSNCSAVRPV-DTPEPARLSSRNNGAVEEFAKPLA
 Consensus         .aqsvWK.klG.$wl..GFeaCv.#L#PYF#rEF.rlq.k.e...Q....t....d...a..r.v.#a...v...s....a...f.k.lA

                   181                                                                                     270
TrDCL2_Choachí     RLFVAVLTFTRCPR-NLKELKQGWFSATAILQHLQCNDYG-RVKSNDGEAAEHTEAWMPTTPEQLVSLLLRLNVLRRLQFCW--------
TrDCL2_Sc58        RLFVAVLTFTRCPR-NLKELKQGWFSATAILQHLQCNDYG-RVKSNDGEAAEHTEAWMPTTPEQLVSLLLRLNVLRRLQFCW--------
TcIL3000.3.480     KLYSDALRCVRGVKCNDGAE-GGWVAAGDLFASVSAHKAGYMMKASAIAEVERSTLPFPTTPSGLVNVLSKLDLLGRFQFRRLCGAESEP
TcIL3000.0.20050   KLYSDALRCVRGVKCNDGTD-GGWVAAGDLFASVSAHKAGYMMKASAIAEVERSSLPFPTTPSGLVKVLSKLDLLGRFQFRRLCGAESEP
Tbg972.3.980       SLYEASLRYLRSVGMKTTASTNGWVSAEELYSYLKDRKHSLIVKVPDDIVSECDVLP--TTPARLLVVLSQLDLIGRLQFRWE--SKVDG
Tb427.03.1230      SLYEASLRYLRSVGMKTTASTNGWVSAEELYSYLKDRKHSLIVKAPDDIVSECDVLP--TTPARLLVVLSQLDLIGRLQFRWERSSKVDG
Tb927.3.1230       SLYEASLRYLRNVGMKTTASTNGWVSAEELYSYLKDRKHSLIVKVPDDIVSECDVLP--TTPARLLVVLSQLDLIGRLQFRWERSSKVDG
 Consensus         .L%.a.Lr..R.v..n......GWvsA..l...l...k.g..vK..d....E...lp.pTTP..Lv.vLs.L#llgRlQFrw........

                   271                                                                                     360
TrDCL2_Choachí     ---GSRVKGAPAKTAGDVWVRAAWAHYARRIGDEVFESHPLLPLEQVLSQFSSAVEFYDYIEHVDFWPSQVKDHGGRTRSDFRPFLLLVS
TrDCL2_Sc58        ---GSRVKGAPAKTAGDVWVRAAWAHYARRIGDEVFESHPLLPLEQVLSQFSSAVEFYDYIEHVDFWPSQVKDHGGRTRSDFRPFLLLVS
TcIL3000.3.480     TGEANGGAAAEGLQEEKYSIRAAWAHKDSFMAQAVLSSHPMMTLKEAIDKFGRGKRLYDFVESLDRWRAELTKGG-RSVPVFRPFLLL-A
TcIL3000.0.20050   TGEANGGAAAEGLQEEKYSVRAAWAHKDFFMAQAVLSSHPMMTLKEAIDKFGRGKRLYDFVESLDRWKAELTKGG-RSVPVFRPFLLL-A
Tbg972.3.980       KRDDERMSLGDEQEECSVSVRAAWAHKDSSLASKVVSDHPKMALEKALEIFSPCREIYDFVESVERWEATAKLHG-REIPTFRPFLML-A
Tb427.03.1230      KRDDERMSLGDKQEECSVSVRAAWAHKDSSLASKVVSDHPKMALEKALEIFSPCREIYDFVESVERWEATAKLHG-REIPTFRPFLML-A
Tb927.3.1230       KRDGERMSLGDEQVECSVSVRAAWAHKDSSLASKVVSDHPKMALEKALEIFSPCREIYDFVESVERWEVAAKLHG-REIPTFRPFLML-A
 Consensus         .....r...a....e..vs!RAAWAHkd...a..V.ssHP.$.Le.al..Fs...e.YD%!Esv#rW.a..k.hG.R..p.FRPFL$L.a

                   361                                                                                     450
TrDCL2_Choachí     VSLLCQGYERCEGMAWDTVLAANKITSNSAFVRVRKTFINLHRQRRVRVVHP--------LPYSSAASGSPDEGVLLLLLSEMDSDVITR
TrDCL2_Sc58        VSLLCRGYERCEGMAWDTVLAANKITSNSAFVRVWKTFINLHRQRRVRVVHP--------LPYSSAASGSPDEGVLLLLLSEMDSDVITR
TcIL3000.3.480     VGDVIESRITGDCTQQAATLLSNKVVGGSAFLPIHEHFLNMHEECRVHVVHSHFVNGVGAVPFEAAPGGESCGVVFLLPVSEIPVGNVLK
TcIL3000.0.20050   VGDVIESRITGDCTQQAATLLSNKVVSGSAFLPIHEHFLNMHEECRVHVVHSHFVNGVGAVPFEAAPGGESCGVVFLLPVSEIPVGNVLK
Tbg972.3.980       VGDSIRARIPKDPVRRAAALLASKVTTKSAFLSVSRSFLGLHLQRPVCVVHSNIFEGGAMHLRQTAGHGQPPRGVLLLHLSDIVYNAISN
Tb427.03.1230      VGDSIRARIPKDPVRRAAALLASKVTTKSAFLSVSRSFLGLHLQRPVCVVHSNIFEGGAMHLRQTAGHGQPPRGVLLLHLSDIVYNAISN
Tb927.3.1230       VGDSIRARIPKDPVRRAAALLASKVTTKSAFLSVSRSFLGLHLQRPVCVVHSNIFEGGAMHLRQTAGHGQPPRGVLLLHLSDIVYNAISN
 Consensus         Vgd.i..ri..#....aa.LlanK!ts.SAFl.!...Fln$H.#rrV.VVHs....g....p...A..G.p..gVlLL.lS#i.....!..

                   451                                                                                     540
TrDCL2_Choachí     --SSKGTKYIRAEALYGDGVSTEITVEALRLLNQKECEGSACTDNNVKSDIGSSSSGDGAQLCRFTWQLTQLCGQARATELSSKDDEVKN
TrDCL2_Sc58        --SSKGTKYIRAEALYGDGVSTEITVEALRLLNQKECEGSACTDNNVKSDIGSSSSGDGAQLCRFTWQLTQLCGQARATELSSKDDEVKN
TcIL3000.3.480     NSENPQILYVKGEALY--GEA-QLRGVPLDALCLEAGEHSLGGD-------GASGGVGAVQSCRIAVKEVVLDLPVEPQVSGSPALVNSN
TcIL3000.0.20050   NTENPQILYVKGETLY--GEP-QLRGVPLDALCLEAGEHSLGGD-------GAGGGVGAVQSCRIAVKEVVLDLPVGPQVSGSPALVNSN
Tbg972.3.980       VDTKRGALFVKSEILYCGDAS-SVRSEPIKSLCLETEEPKTCTE-------PPS--TSALLFARLAEKSITFNVPAESPRVSSPTLANRI
Tb427.03.1230      VDTKRGALFVKSEILYCXDAS-SVRSEPIKSLCLETEEPKTCTE-------PPS--TSALLFARLAEKSITFNVPTESPRVSSPTLANRI
Tb927.3.1230       VDTKRGALFVKSEILYCGDAS-SVRSESIKSLCLETEEPKTCTE-------PPS--TSALLFARLAEKSITFNVPAESPRVSSPTLANRI
 Consensus         .....g.l%!k.EaLY..g.s...r.epl..Lcle..E.s.ct#.......g.s....a.q.cR.a.k...l..pa.....sSp.l.n.n

                   541                                                                                     630
TrDCL2_Choachí     GKTDKDNRLETQSLIYFDIEAALRCLRFVAAEASDAFAAYGEFDLGVVCSRWLAVGSSLPSCLANGNNLTATLSAVDAFAAPSLIWPCMR
TrDCL2_Sc58        GKTDKDNRLETQSLIYFDIEAALRCLRFVAAEASDAFAAYGEFDLGVVCSRWLAVGSSLPSCLANGNNLTATLSAVDAFAAPSLIWPCMR
TcIL3000.3.480     VFDEKTDTELDPAVWLQHLRYISQRLLLVTKESF--CKEWLEGIFDSYK--EDKSCEDVSKMITN----ALSGSFEYAHSSLSLLVPRGG
TcIL3000.0.20050   VFDEKSDTELDPAVWLQHLRYISQRLLLVTKESF--CKEWLEGIFDSYK--EDKSCEDVSKMITN----VLSGSFEYAHSSLSLLVPRGR
Tbg972.3.980       LTLKRPTPTTNATEFLCGLRSLSHGMGNFAREAF--FKKWIEDILALLRARNNRNGSDRSNSCRTPTEKVLSVTPDCVYTDFSLIIAGKQ
Tb427.03.1230      LTLKRPTPTTNATEFLCGLRSLSHGMGNFAREAF--FKKWIEDILALLRARNNRNGSDRSNSCRTPTEKVLSVTPDCVYTDFSLIIAGKQ
Tb927.3.1230       LTLKRPTPTTNATEFLCGLRSLSHGMGNFAREAF--FKKWIEDILALLRARNNRNGSDRSNSCRTPTEKVLSITPDCVYTDFSLIIAGKQ
 Consensus         ....k.........l..lr..s..$..va.Eaf..fk.w.E.il.....r....gsd.s....n.....ls.s...a....SLi.p..r
```

A) continued

```
                   631                                                                                      720
TrDCL2_Choachí     CSDGAVPYGRQVAMKELRVELRAGTFVPLYVSSQRQKQPFTVYFAQRTLAGVNGGAPLATTPMVLLRPQRTVDVRQTAPVSESEKAIPRS
TrDCL2_Sc58        CSDGAVPYGRQVAMKELRVELRAGTFVPLYVSSQRQKQPFTVYFAQRTLAGVNGGAPLATTPMVLLRPQRTVDVRQTAPVSESEKAIPRP
TcIL3000.3.480     TGMRLSQKGQPPL-ETLTNAAGRGECDPLYISANRQRQ-VVVYFHADSPWGAD-QETVQQTVLLFLSSSQWEPPGADEE-SGAKLSVDGR
TcIL3000.0.20050   TGVRLSQKGRPPL-ETLTNAAGRGECDPLYISANRQRQ-VVVYFHADSPWGAD-QETVQQTVLLFLSSSQWEPPGADEE-GGAKLLVDGR
Tbg972.3.980       KKDNSRPPGLRSLSKELINGIAEGGCDPLYIKSKQEGT-IVVYFQSTVSDERDVTEFFFSSPLLLLRSSYTVGDNEGKQ-TEEETSVANH
Tb427.03.1230      KKDNSRPPGLRSLSKELINGIAEGGCDPLYIKSKQEGT-IVVYFQSTVSDERDVTEFFFSSPLLLLRSSYTVGDNEGKQ-TEEETSVANH
Tb927.3.1230       KKDNSRPPGLRSLSKELINGIAEGGCDPLYIKSKQEGT-IVVYFQSTVSDERDVTEIFFSSPLLLLRSSYTVGDNEGKQ-TEEETSVANH
 Consensus         ..d...p.Gr..l.keL.n....G.cdPLY!ss.r#.q..vVYF......g.#..e....tp$llLrss.tv........se.e..!...

                   721                                                                                      810
TrDCL2_Choachí     RAALVLSYISLQDDECGDGAVFIATELSEEDEARYRGDRDYEDIHPDEDNHSDGEGGALRWSAVIVDEWELEAAKPIAEVVEREFMLPLE
TrDCL2_Sc58        RAALVLSYISLQDDECGDGAVFIATELSEEDEARYRGDRDYEDIHPDEDNHSDGEGGALRWSAVIVDEWELEAAKPIAEVVEREFMLPLE
TcIL3000.3.480     TAVLLLP-SKAQRKNGAGGDFFRTRTAVELSVAQ---QSDAEAMYGI-----CGSTGSLRSVELLVDELAFEVLTPITDIVDTMLLAPVK
TcIL3000.0.20050   TAVLLLP-SKAQRKNGAGGDFFRTRTAVELSVAQ---QSDAEAMCGI-----CGSTGSLRSVELLVDELAFEVLTPITDIVDTMLLAPVK
Tbg972.3.980       RSALFLR-VDKSSDSFAEGDTFRSLTVVELDREQ---KKVAEALRSD-----CNTEVTLRSVELCVDEWCLEVLVPLEKVVEECIFAPIK
Tb427.03.1230      RSALFLR-VDKSSDSFAEGDTFRSLTVVELDREQ---KKVAEALRSD-----CNTEVTLRSVELCVDEWCLEVLVPLEKVVEECIFAPIK
Tb927.3.1230       RSALFLR-VDKSSDSFAEGDTFRSLTVVELDREQ---KKVAEALRSD-----CNTEVTLRSVELCVDEWCLEVLVPLEKVVEEYIFAPIK
 Consensus         raaL.L.....q.d..a.Gd.Fr..t.vEld.aq.....daEa...d.....cg..g.LRsvel.VDEw.lEvl.Pi..!V#....aP.k

                   811                                                                                      900
TrDCL2_Choachí     RSLCT-PNPACRFHNFPVEVQDALLQSLRMQLLLAVTPQLNEKLEYVGDAILDFVVAQDKLSIWTAGRIVSESSNRRFVSYLPEVITHHL
TrDCL2_Sc58        RSLCT-PNPACRFHNFPVEVQDALLQSLRMQLLLAVTPQLNEKLEYVGDAILDFVVAQDKLSIWTAGRIVSESSNRRFVSYLPEVITHHL
TcIL3000.3.480     RSLSSGKDATLPFLSLPVEARATIIQSLRQYLLMAVQPLINETLEFLGDAVLDFVVAQETLNTWGGGPVAEATSNIQLGRSLPLPLIRYY
TcIL3000.0.20050   RSLSSGKDATLPFLSLPVEARATIIQSLRQYLLMAVQPLINETLEFLGDAVLDFVVAQETLNTWGGGPVAEATSNIQLGRSLPLPLIRYY
Tbg972.3.980       QCLYGRGNSESPFLLLSEADQAKLEQSLMEQLLLAVTPVKNEALEYLGDAVLDFVVAQEKLNSWTGGPIVDATCNASLAKCLFPQLRNYF
Tb427.03.1230      QCLYGRGNSESPFLLLSEADQAKLEQSLMEQLLLAVTPVKNEALEYLGDAVLDFVVAQEKLNSWTGGPIVDATCNASLAKCLFPQLRNYF
Tb927.3.1230       QCLYGRGNSESPFLLLSEADQAKLEQSLMEQLLLAVTPVKNEALEYLGDAVLDFVVAQEKLNSWTGGPIVDATCNASLAKCLFPQLRNYF
 Consensus         rsL....#...pFl.lpve.qa.l.QSLr.qLL$AVtP..NE.LE%lGDA!LDFVVAQ#kLn.WtgGp!v.atsN..l...Lp..l..y.

                   901                                                                                    989
TrDCL2_Choachí     RLVWHIGNEKRKADVVEALFGAVVMALWVIPYSGVARGPLS-------------AAGRLPFTCVLQVVRELMSVLQVVVT
TrDCL2_Sc58        RLVWHIGNEKRKADVVEALFGAVVMALWVIPYSGVARGPLS-------------AAGRLPFTCVLQVVRELMSVLHVVVT
TcIL3000.3.480     ELKFKITNKKRHADTVEAIFGALAMVLWVIPFRDEGKHTAD---------------GPLPFSVLQCVTRDLMTALGICAQAETEPSDQP
TcIL3000.0.20050   ELKFKITNKKRHADTVEAIFGALAMVLWVIPFRGEGKHTAD---------------GPLPFSVLQCVTRDLMTALGICAQAETEPSGQP
Tbg972.3.980       EMKLGISNQKRQADMVEAIFGAVAMALWVIPRRKVLESSTDTTVTGSGGGETPAAGGLLPFAALLSVTKALMDALNITTQ
Tb427.03.1230      EMKLGISNQKRQADMVEAIFGAVAMALWVIPRRKVLESSTDTTVTGSGGGETPAAGGLLPFAALLSVTKALMDALNITTQ
Tb927.3.1230       EMKLGISNQKRQADMVEAIFGAVAMALWVIPRRKVLESSTDTTVTGGGGGETPAAGGLLPFAALLSVTKALMDALNITTQ
 Consensus         e$k..I.N.KR.AD.VEAiFGAvaMaLWVIP.rgv.....d.............a.G.LPF..ll.Vtr.LM.aL.!..q.........
```

B)

Organism		*T. rangeli*		*T. congolense*		*T. b. gambiense*	*T. b. brucei*	
		Sc-58	Choachí	TcIL3000.3.480	TcIL3000.0.20050	Tbg972.3.980	Tb927.3.1230	Tb427.03.1230
T. rangeli	Sc-58	100						
	Choachí	99	100					
T. congolense	TcIL3000.3.480	26	26	100				
	TcIL3000.0.20050	25	25	97	100			
T. b. gambiense	Tbg972.3.980	29	29	36	36	100		
T. b. brucei	Tb927.3.1230	29	29	36	36	99	100	
	Tb427.03.1230	29	29	36	36	98	98	100
