# Supplementary material for: Genome of the Avirulent Human-Infective Trypanosome—Trypanosoma rangeli
Source: PLoS Negl Trop Dis. 2014 Sep 18;8(9):e3176. doi: 10.1371/journal.pntd.0003176 (PMC4169256; doi:10.1371/journal.pntd.0003176)
Supplement: Table S1 — Comparison of satellite DNA found in T. rangeli strain SC-58 genomic and transcriptomic libraries with the T. cruzi haploid genome (CL Brener strain). (DOCX) [file pntd.0003176.s006.docx]

**Supplementary Table 1 –** Comparison of satellite DNA found in *Trypanosoma rangeli* strain SC-58 genomic and transcriptomic libraries to *T. cruzi* haploid genome (CL Brener strain).

|  | ***T. rangeli*  (transcriptome)** | ***T. rangeli* (genome)** | | | | | ***T. cruzi* (genome)** | | | | |
| --- | --- | --- | --- | --- | --- | --- | --- | --- | --- | --- | --- |
| **Total bases analyzed (Mb)** | 2.45 | 20.39 | | | | | 32.5 | | | | |
|  | **Microsatellites (1-6 bp)** | **Microsatellites (1-6 bp)** | **Unclassified  (7-11 bp)** | **Minisatellites  (12-100 bp)** | **Satellites (>100 bp)** | **Total** | **Microsatellites  (1-6 bp)** | **Unclassified  (7-11 bp)** | **Minisatellites  (12-100 bp)** | **Satellites (>100 bp)** | **Total** |
| **Total number of repeat *loci*** | 1,997 | 42,459 | 15,804 | 5,517 | 21 | 63,801 | 52,037 | 23,087 | 8,481 | 112 | 83,717 |
| **Total number of classes** | 198 | 400 | - | - | - | - |  | - | - | - | - |
| **Non Redundant repeat bases (bp)** | 22,349 | 788,637 | 311,707 | 168,718 | 1,166 | 1,270,228 | 1,012,615 | 473,185 | 284,544 | 14,230 | 1,784,574 |
| **Relative abundance (%)** | 0.91 | 3.87 | 1.53 | 0.83 | 0.01 | 6.23 | 3.12 | 1.46 | 0.88 | 0.04 | 5.49 |
| **Density^a^ (pb / Mb)** | 9,133 | 38,678 | 15,287 | 8,275 | 57 | 62,297 | 31,157 | 14,560 | 8,755 | 438 | 54,910 |
| **Frequency^b^**  **(1 / *n*Kb)** | 1/1.22 | 1/0.48 | 1/1.29 | 1/3.70 | 1/970 | 1/0.32 | 1/0.62 | 1/1.41 | 1/3.83 | 1/290 | 1/0.39 |

a – Number of bases in repeat sequences / total bases analyzed;

b – 1 repeat / *n*Kb analyzed;
