# Supplementary material for: Genome of the Avirulent Human-Infective Trypanosome—Trypanosoma rangeli
Source: PLoS Negl Trop Dis. 2014 Sep 18;8(9):e3176. doi: 10.1371/journal.pntd.0003176 (PMC4169256; doi:10.1371/journal.pntd.0003176)
Supplement: Table S3 — Comparative number of translation process-related proteins from distinct kinetoplastid species. (DOC) [file pntd.0003176.s008.doc]

**Supplementary Table 3– Comparative number of translation process-related proteins from distinct kinetoplastid species.**

| **Protein** | ***T. rangeli*** | ***T. cruzi*** | ***T. brucei*** | ***T. gambiense*** | ***T. vivax*** | ***L. braziliensis*** | ***L. donovani*** | ***L. infantum*** | ***L. major*** | ***L. mexicana*** |
| --- | --- | --- | --- | --- | --- | --- | --- | --- | --- | --- |
| **tRNA-synthetases** | | | | | | | | | | |
| arginyl-tRNA synthetase | 1 | 1 | 1 | 1 | 1 | 1 | 1 | 1 | 1 | 1 |
| glutaminyl-tRNA synthetase | 3 | 3 | 1 | 1 | 1 | 1 | 1 | 1 | 1 | 1 |
| leucyl-tRNA synthetase | 2 | 2 | 1 | 1 | 1 | 1 | 1 | 1 | 1 | 1 |
| histidyl-tRNA synthetase | 1 | 1 | 1 | 1 | 1 | 1 | 1 | 1 | 1 | 1 |
| aspartyl-tRNA synthetase | 3 | 6 | 2 | 2 | 1 | 2 | 2 | 2 | 2 | 2 |
| glycyl-tRNA synthetase | 1 | 3 | 1 | 1 | 1 | 1 | 1 | 1 | 1 | 1 |
| lysyl-tRNA synthetase | 2 | 3 | 2 | 2 | 2 | 2 | 2 | 2 | 2 | 2 |
| prolyl-tRNA synthetase | 1 | 2 | 1 | 1 | 1 | 1 | 2 | 2 | 1 | 2 |
| threonyl-tRNA synthetase | 1 | 2 | 1 | 1 | 1 | 1 | 1 | 1 | 1 | 1 |
| isoleucyl-tRNA synthetase | 1 | 2 | 1 | 1 | 1 | 1 | 1 | 1 | 1 | 1 |
| alanyl-tRNA synthetase | 1 | 2 | 1 | 1 | 1 | 1 | 1 | 1 | 1 | 1 |
| phenylalanyl-tRNA synthetase | 1 | 2 | 1 | 1 | 1 | 1 | 1 | 1 | 1 | 1 |
| phenylalanyl-tRNA synthetase alpha chain | 1 | 1 | 1 | 1 | 1 | 1 | 1 | 1 | 1 | 1 |
| valyl-tRNA synthetase | 2 | 3 | 1 | 1 | 1 | 1 | 1 | 1 | 1 | 1 |
| glutamyl-tRNA synthetase | 1 | 2 | 1 | 1 | 1 | 1 | 1 | 1 | 1 | 1 |
| cysteinyl-tRNA synthetase | 1 | 2 | 1 | 1 | 1 | 1 | 1 | 1 | 1 | 1 |
| tyrosyl-tRNA synthetase | 1 | 1 | 2 | 2 | 2 | 1 | 1 | 1 | 1 | 1 |
| tyrosyl or methionyl-tRNA synthetase | 1 | 2 | 1 | 1 | 1 | 1 | 1 | 1 | 1 | 1 |
| asparaginyl-tRNA synthetase | 1 | 1 | 1 | 1 | 1 | 1 | 1 | 1 | 1 | 1 |
| tryptophanyl-tRNA synthetase | 2 | 10 | 2 | 1 | 2 | 2 | 2 | 2 | 2 | 2 |
| seryl-tRNA synthetase | 2 | 2 | 1 | 1 | 1 | 1 | 1 | 1 | 1 | 1 |
| methionyl-tRNA synthetase | 1 | 1 | 1 | 1 | 4 | 1 | 1 | 1 | 1 | 1 |
| **Translation factors** | | | | | | | | | | |
| eukaryotic translation initiation factor 1A | 1 | 2 | 1 | 1 | 1 | 1 | 1 | 1 | 1 | 1 |
| initiation factor IF-2 | 3 | 6 | 3 | 3 | 3 | 3 | 3 | 3 | 3 | 3 |
| initiation factor eIF-2 alpha subunit | 1 | 2 | 1 | 1 | 1 | 1 | 1 | 1 | 1 | 1 |
| initiation factor eIF-2 beta subunit | 1 | 2 | 1 | 1 | 1 | 1 | 1 | 1 | 1 | 1 |
| initiation factor eIF-2 gamma subunit | 1 | 2 | 1 | 1 | 1 | 1 | 1 | 1 | 1 | 1 |
| translation initiation factor eIF-2B alpha subunit | 1 | 2 | 1 | 1 | 1 | 1 | 1 | 1 | 1 | 1 |
| translation initiation factor eIF-2B beta subunit | 1 | 2 | 1 | 1 | 1 | - | 1 | 1 | 1 | 1 |
| translation initiation factor eIF2B delta subunit | 1 | 2 | 1 | 1 | 1 | 1 | 1 | 1 | 1 | 1 |
| translation initiation factor eIF2B gamma subunit | - | - | - | - | - | 1 | 1 | 1 | 1 | 1 |
| translation initiation factor eIF2B epsilon subunit | 1 | 2 | 1 | 1 | 1 | 1 | 1 | 1 | 1 | 1 |
| eukaryotic translation initiation factor 3 subunit I (or subunit 2) | 1 | 2 | 1 | 1 | 3 | 1 | 1 | 1 | 1 | 1 |
| eukaryotic translation initiation factor 3 subunit E (or subunit 6) | 1 | 2 | 1 | 1 | 1 | 1 | 1 | 1 | 1 | 1 |
| eukaryotic translation initiation factor 3 subunit D (or subunit 7) | 1 | 2 | 1 | 1 | 1 | 1 | 1 | 1 | 1 | 1 |
| eukaryotic translation initiation factor 3 subunit L (or subunit 6-interacting protein) | 1 | 2 | 1 | 1 | 1 | 1 | 1 | 1 | 1 | 1 |
| eukaryotic translation initiation factor 3 subunit C (or subunit 8) | 1 | 2 | 2 | 1 | 1 | 1 | 1 | 1 | 1 | 1 |
| eukaryotic translation initiation factor 3 subunit B (or subunit 9) | 2 | 1 | 1 | 1 | 1 | 1 | 1 | 1 | 1 | 1 |
| eukaryotic translation initiation factor 4E | 4 | 9 | 4 | 4 | 3 | 4 | 3 | 3 | 4 | 3 |
| eukaryotic translation initiation factor 5 | 1 | 2 | 1 | 1 | 1 | 1 | 1 | 1 | 1 | 1 |
| eukaryotic initiation factor 5a | 3 | 2 | 1 | 1 | 1 | 4 | 3 | 3 | 2 | 2 |
| eukaryotic translation initiation factor 6 (eIF-6) | 1 | 1 | 1 | 1 | 1 | 1 | 1 | 1 | 1 | 1 |
| elongation factor 1-alpha (EF-1-alpha) | 8 | 11 | 4 | 4 | 5 | 2 | 3 | 8 | 8 | 8 |
| elongation factor 1-beta | 1 | 3 | 3 | 3 | 3 | 5 | 3 | 4 | 4 | 4 |
| elongation factor 1-gamma (EF-1-gamma) | 5 | 35 | 2 | 2 | 2 | 2 | 3 | 3 | 2 | 2 |
| translation elongation factor EF-2 | 4 | 8 | 3 | 2 | 3 | 4 | 3 | 4 | 3 | 2 |
| mitochondrial elongation factor G | 1 | 3 | 1 | 1 | 1 | 1 | 1 | 1 | 1 | 1 |
| elongation factor G2-like protein | 1 | 4 | 1 | 1 | 1 | 1 | 1 | 1 | 1 | 1 |
| elongation factor Tu | 1 | 2 | 1 | 1 | 1 | 1 | 1 | 1 | 1 | 1 |
| selenocysteine-tRNA-specific elongation factor | 1 | 2 | 1 | 1 | 1 | 1 | 1 | 1 | 1 | 1 |
| eukaryotic peptide chain release factor subunit 1 | 2 | 3 | 2 | 2 | 2 | 2 | 1 | 2 | 2 | 1 |
| eukaryotic peptide chain release factor 3 | 1 | 2 | 1 | 1 | 2 | 2 | 1 | 1 | 1 | 1 |
| peptide chain release factor 1 | 2 | 3 | 1 | 1 | 1 | 1 | 1 | 1 | 1 | 1 |

*Sequences from other organisms were retrieved from GeneDB annotations (http://www.genedb.org/Homepage). Some sequences were retrieved through *Blast* searches in the database.
