# Supplementary material for: Genome of the Avirulent Human-Infective Trypanosome—Trypanosoma rangeli
Source: PLoS Negl Trop Dis. 2014 Sep 18;8(9):e3176. doi: 10.1371/journal.pntd.0003176 (PMC4169256; doi:10.1371/journal.pntd.0003176)
Supplement: Table S4 — Trypanosoma rangeli ePKs with predicted transmembrane domains. (DOCX) [file pntd.0003176.s009.docx]

**Supplementary Table 4:** *Trypanosoma rangeli* ePKs with predicted transmembrane domains.

| **gene ID** | **ePK Group** | **TMD annotation*** |
| --- | --- | --- |
| AUPL00006967 | STE/STE 11 | TM |
| AUPL00003247 | OTHER/Uni1 | TM |
| AUPL00003518 | STE/SBK1 | TM |
| AUPL00003639 | CAMK | TM |
| AUPL00004000 | OTHER/NEK | **2xTM |
| AUPL00004023 | OTHER/ULK/ULK | 4xTM |
| AUPL00004079 | CAMK | SP |
| AUPL00004570 | OTHER/PEK/PKR | SP TM |
| AUPL00004767 | AGC | SP |
| AUPL00005624 | STE/STE11 | 3xTM |
| AUPL00006473 | OTHER/NEK | SP |
| AUPL00006494 | OTHER/NEK | SP |
| AUPL00003247 | OTHER/Uni1 | TM |
| * Annotation on InterProScan and SMART; TM transmembrane domain; SP, signal peptide;**number of occurrences | | |
