# Supplementary material for: Genome of the Avirulent Human-Infective Trypanosome—Trypanosoma rangeli
Source: PLoS Negl Trop Dis. 2014 Sep 18;8(9):e3176. doi: 10.1371/journal.pntd.0003176 (PMC4169256; doi:10.1371/journal.pntd.0003176)
Supplement: Table S5 — Phosphatidylinositol and related kinase proteins identified from the predicted proteomes of T. rangeli and T. cruzi . (DOCX) [file pntd.0003176.s010.docx]

**Supplementary Table 5 -** Phosphatidylinositol and related kinase proteins identified from the predicted proteomes of *T. rangeli* and *T. cruzi*.

| Model/PIK class | *T. rangeli* gene | *T. cruzi* gene | Main Domains and Modular Architecture | Classification* |
| --- | --- | --- | --- | --- |
| 1/PI3 Class III | AUPL00004184 | Tc00.1047053511903.160 | 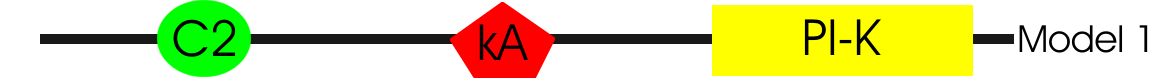 |  |
| 2/PI3 Class I | AUPL00003334 | Tc00.1047053508859.90 | 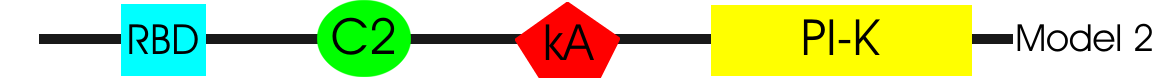 | Conventional PIK |
| 3/PI4 IIIβ | AUPL00004997 | Tc00.1047053508695.20 | 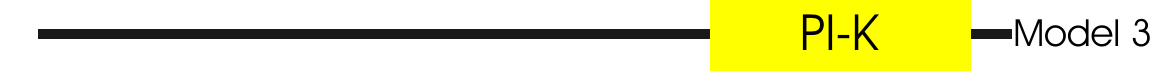 |  |
| 4/PI4 IIIα | AUPL00002444 | Tc00.1047053510003.30 | 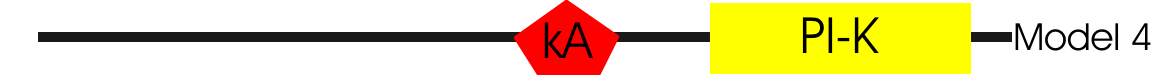 |  |
|  |  |  |  |  |
|  | AUPL00006561**^a^** | Tc00.1047053508231.30**^a^** |  |  |
|  | AUPL00006093**^b^** | Tc00.1047053508257.230**^b^** |  |  |
| 5* | AUPL00006676**^c^** | Tc00.1047053510689.40**^c^** | 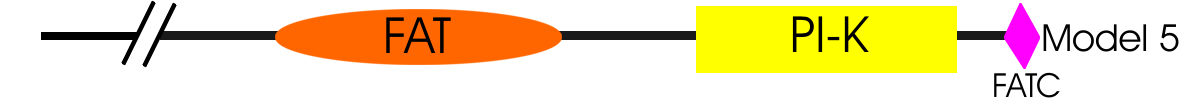 | PIK-related |
|  | AUPL00006527**^d^** | Tc00.1047053509395.20**^d^** |  |  |
|  | AUPL00006491**^e^** | Tc00.1047053506223.120**^e^** |  |  |

*****according [86, 122] **^a^** Tor 1, **^b^**Tor-like1, **^c^**Tor 2, **^d^**ATM, **^e^**AT
