# Supplementary material for: Genome of the Avirulent Human-Infective Trypanosome—Trypanosoma rangeli
Source: PLoS Negl Trop Dis. 2014 Sep 18;8(9):e3176. doi: 10.1371/journal.pntd.0003176 (PMC4169256; doi:10.1371/journal.pntd.0003176)
Supplement: Table S6 — Accessory domains present in PIK-related proteins in T. rangeli and T. cruzi (Model 5). (DOCX) [file pntd.0003176.s011.docx]

**Supplementary Table 6:** Accessory domains present in PIK-related proteins in *Trypanosoma rangeli* and *Trypanosoma cruzi* (Model 5)

|  | Gene |  |  | Accessory Domain | | | | | |
| --- | --- | --- | --- | --- | --- | --- | --- | --- | --- |
|  |  |  | FRAP | | HEAT | PDZ | DUF3385 | ATM | UME |
| *T. cruzi* | TcCLB.508231.30 |  | + | | * |  | + |  |  |
|  | TcCLB.508257.230 | | + | | * | + | + |  |  |
|  | TcCLB.510689.40 |  | + | | * |  | + |  |  |
|  | TcCLB.509395.20 |  |  | |  |  |  | **Δ** |  |
|  | TcCLB.506223.120 | |  | |  |  |  |  | * |
|  |  |  |  | |  |  |  |  |  |
| *T. rangeli* |  |  |  | |  |  |  |  |  |
|  | AUPL00006561 |  | + | | * |  | + |  | * |
|  | AUPL00006093 |  | + | | * | + | + |  | * |
|  | AUPL00006676 |  | + | | * |  | + |  | * |
|  | AUPL00006527 |  |  | |  |  |  | **Δ** |  |
|  | AUPL00006491 |  |  | | * |  |  |  | * |
|  |  |  |  | |  |  |  |  |  |

* E-value > 10^-2^

**Δ** domain only found by using Interproscan database

**+** domain found by all databases used (Pfam, Interproscan, Motif Scan, SMART)

**FRAP** (FKBP12-rapamycin-associated protein, FKBP12-rapamycin-binding) (PF08064)**; HEAT** (PF02985); **PDZ** (PF00595, PF00595); **ATM**, Ataxia-Telangiectasia Mutated (IPR015519); **UME domain** (IPR012993, PF08064); **DUF3385**(PF11865)
