# Supplementary material for: Genome of the Avirulent Human-Infective Trypanosome—Trypanosoma rangeli
Source: PLoS Negl Trop Dis. 2014 Sep 18;8(9):e3176. doi: 10.1371/journal.pntd.0003176 (PMC4169256; doi:10.1371/journal.pntd.0003176)
Supplement: Table S7 — Antioxidant enzymes of trypanosomatids. (DOCX) [file pntd.0003176.s012.docx]

|  | *T. rangeli* | *T. cruzi* | *L. major* | *T. brucei* |
| --- | --- | --- | --- | --- |
| **Endoplasmatic reticulum** |  |  |  |  |
| GSH-dependent peroxidase (GPXII) | AUPL00001013 | ✓ | ✓ | ✓ |
| Ascorbate-dependent hemoperoxidase (APX) | X | ✓ | ✓ | X |
| **Mitochondria** |  |  |  |  |
| Fe-containing form of superoxide dismutase (FeSOD-A) | AUPL00000796 | ✓ | ✓ | ✓ |
| Mitochondrial peroxiredoxin (MPX) | AUPL00004568 | ✓ | ✓ | ✓ |
| **Cytosol** |  |  |  |  |
| Cytosolic peroxiredoxin (CPX) | AUPL00002563 | ✓ | ✓ | ✓ |
| Fe-containing form of superoxide dismutase B(FeSOD-B) | AUPL00005576 | ✓ | ✓ | ✓ |
| GSH-dependent peroxidase (GPXI) | AUPL00007183 | ✓ | ✓ | ✓ |
| **Spermidine Synthesis** |  |  |  |  |
| Ornithine Descarboxylase | X | X | ✓ | ✓ |
| **Trypanothione Synthesis** |  |  |  |  |
| Trypanothione synthetase (TS) | AUPL000[03866](http://ligeirinha.lncc.br/tr-final-bin/annotation.cgi?id=0&gene=TR03866)  AUPL000[02894](http://ligeirinha.lncc.br/tr-final-bin/annotation.cgi?id=0&gene=TR02894)  AUPL000[01995](http://ligeirinha.lncc.br/tr-final-bin/annotation.cgi?id=0&gene=TR01995) | ✓ | ✓ | ✓ |
| Trypanothione reductase (TR) | AUPL00002517 | ✓ | ✓ | ✓ |

**Supplementary table 7** - Antioxidant enzymes in trypanosomatids.
