# Supplementary material for: Genome of the Avirulent Human-Infective Trypanosome—Trypanosoma rangeli
Source: PLoS Negl Trop Dis. 2014 Sep 18;8(9):e3176. doi: 10.1371/journal.pntd.0003176 (PMC4169256; doi:10.1371/journal.pntd.0003176)
Supplement: Table S8 — The stress response proteins of T. rangeli . (DOC) [file pntd.0003176.s013.doc]

**Supplemetary Table 8 -** The stress response proteins of *T. rangeli*.

| Heat stress | Genes | ORF id |
| --- | --- | --- |
| HSP10 | 2 | AUPL00001781, AUPL00001955 |
| HSP100 | 1 | AUPL00006000 |
| HSP20 | 1 | AUPL00004685 |
| HSP40 | 24 | AUPL00000466, AUPL00000469, AUPL00000494, AUPL00001015, AUPL00001140, AUPL00001429, AUPL00002002, AUPL00002132, AUPL00002336, AUPL00002672, AUPL00002745, AUPL00002941, AUPL00003302, AUPL00003346, AUPL00003359, AUPL00003674, AUPL00003676, AUPL00003846, AUPL00003898, AUPL00003944, AUPL00004291, AUPL00004415, AUPL00005025, AUPL00005645 |
| HSP60 | 5 | AUPL00001390, AUPL00004016, AUPL00004835, AUPL00005283, AUPL00006127 |
| TRiC | 5 | AUPL00001153, AUPL00001581, AUPL00001870, AUPL00003738, AUPL00000631 |
| HSP70 | 16 | AUPL00000557, AUPL00007033, AUPL00007144, AUPL00007159, AUPL00006812, AUPL00007016, AUPL00007074, AUPL00003283, AUPL0003366, AUPL00005103, AUPL00006801, AUPL00000658, AUPL00001604, AUPL00001124, AUPL00003943, AUPL00003941 |
| HSP78 | 1 | AUPL00001212 |
| HSP85 | 1 | AUPL00002514 |
|  |  |  |
| Nutritional stress |  |  |
| Autophagin | 0 |  |
| DEAD box helicase DHH1 | 0 |  |
| STI1 | 1 | AUPL00002091 |
|  |  |  |
| Osmotic stress |  |  |
| ENA-type ATPase | 0 |  |
| Acidocalcisomal exopolyphosphatase | 1 | AUPL00006338 |
| Phosphatidylinositol 3-kinase | 5 | AUPL00003334, AUPL00004184, AUPL00006439, AUPL00000335, AUPL00001804 |
|  |  |  |
